# Covalent Organic Frameworks for Photocatalysis

**DOI:** 10.1002/adma.202413118

**Published:** 2024-12-09

**Authors:** Bikash Mishra, Akhtar Alam, Avanti Chakraborty, Bidhan Kumbhakar, Samrat Ghosh, Pradip Pachfule, Arne Thomas

**Affiliations:** ^1^ Department of Chemical and Biological Sciences S. N. Bose National Centre for Basic Sciences Kolkata 700106 India; ^2^ Inorganic and Physical Chemistry Laboratory Council of Scientific and Industrial Research (CSIR) Central Leather Research Institute (CLRI) Chennai 600020 India; ^3^ Technical Research Centre (TRC) S. N. Bose National Centre for Basic Sciences Kolkata 700106 India; ^4^ Functional Materials Institute of Chemistry Technische Universität Berlin Hardenbergstr. 40 10623 Berlin Germany

**Keywords:** CO_2_ reduction, COFs, microporous materials, Photocatalysis, water splitting

## Abstract

The global energy crisis and environmental concerns are driving research into renewable energy and sustainable energy conversion and storage technologies. Solar energy, as an ideal sustainable resource, has significant potential to contribute to the goal of net‐zero carbon emissions if effectively harnessed and converted into a reliable and storable form of energy. Photocatalysts have the potential to convert sunlight into chemical energy carriers. In this respect, covalent organic frameworks (COFs) have shown great promise due to their tunable structure on different length scales, high surface areas, and beneficial optical properties such as broad visible light absorption. This review offers a comprehensive overview of the key developments in COF‐based photocatalysts for various applications, including water splitting, hydrogen peroxide generation, organic transformations, and carbon dioxide and nitrogen reduction. The underlying mechanisms, essential principles for material design, and structure‐function relationships of COFs in various photocatalytic applications are discussed. The challenges faced by COF‐based photocatalysts are also summarized and various strategies to enhance their performance are explained, such as improving crystallinity, regulating molecular structures, tailoring linkages, and incorporating cocatalysts. Finally, critical strategies are proposed for the utilization of photocatalytically generated chemicals into value‐added products.

## Graphical abstract

This review provides an overview of recent advances in covalent organic frameworks (COFs) for photocatalysis, focusing on sustainable energy applications like water splitting, hydrogen peroxide generation, and CO_2_ and N_2_ reduction. It discusses design principles, structure‐function relationships, challenges in COF photocatalysis, and strategies to enhance performance and convert products into value‐added compounds.

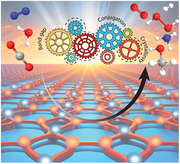

## 1 Introduction

As the world's population, economy, and standard of living have grown, the demand for energy has increased exponentially, resulting in the rapid depletion of fossil fuels, increase of pollution, and global warming.^[^ ^1^, ^2^ ^]^ As a direct result, environmental disasters such as extreme weather events, loss of biodiversity, and accelerated melting of glaciers leading to rising sea levels are becoming more frequent. The main contributor to global warming is carbon dioxide (CO_2_) emissions, which also affect marine life through the acidification of seawater. In this alarming situation, there is an urgent need to tackle CO_2_ emissions by utilizing green energy resources and capturing the CO_2_ present in the atmosphere. At the same time, sustainable energy resources with minimal or no environmental impact in the long term must be explored to address the energy crisis. Solar energy has been the ideal sustainable energy resource since the formation of the Earth and will be available for at least the next few billion years. The amount of solar energy that reaches the Earth every day far exceeds the energy needs of humanity. In figures, the entire surface of the Earth receives an average of 173000 terawatts, which is 10000 times the current energy demand.^[^ ^3^, ^4^ ^]^ The current challenge is to harvest sunlight and store it efficiently in a sustainable and cost‐effective manner. While solar cells are an efficient technology for converting sunlight into electrical energy, they are still not cost‐effective, and storing this energy requires an enormous number of batteries with the existing technologies.^[^ ^5^ ^]^ Alternatively, solar‐generated electricity can be stored as a fuel through the electrochemical process of chemical transformation.^[^ ^6^, ^7^, ^8^, ^9^ ^]^ However, if we consider sustainable pathways, the electrochemical process involves at least two steps: converting solar energy into electricity and then using the electricity generated to produce fuel.^[^ ^10^, ^11^ ^]^ Because it's a two‐step process, overall solar‐to‐fuel efficiency suffers, and it requires expensive infrastructure. An alternative approach to improving solar‐to‐fuel efficiency is to use solar energy directly to produce chemicals that can be used either as fuel or as commodities. This process is known as “photocatalysis” and requires a photocatalyst that can absorb light and simultaneously perform chemical transformations.^[^ ^12^ ^]^ Photocatalysis holds great promise for addressing the energy crisis and climate change. Sunlight‐driven photocatalysis offers the potential to produce green fuels such as hydrogen (H_2_), ammonia (NH_3_), and methanol (CH_3_OH) using abundant resources such as water, nitrogen (N_2_), and CO_2_.^[^ ^13^, ^14^, ^15^ ^]^ It also makes it possible to break down harmful substances that pollute water and air, transforming them into harmless products.^[^ ^16^, ^17^ ^]^

**Figure 1:**
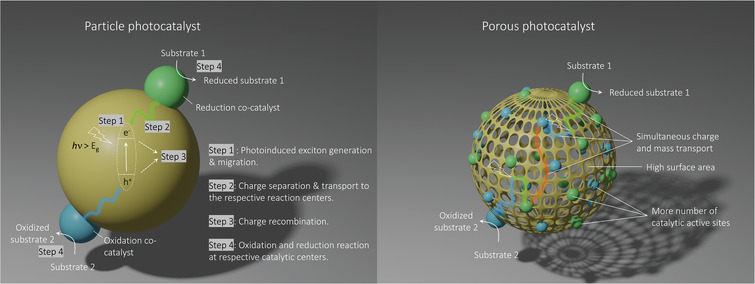
Schematic illustration of the basic photocatalytic processes in the particle photocatalyst (left) and the advantages of the porous photocatalyst (right).

The earliest instance of photocatalysis was reported in the literature in 1911, when Ebner used ZnO as a photocatalyst to bleach Prussian blue dye in the presence of light. Later, in 1921, Baur and Rebmann demonstrated photo‐assisted water splitting using an AgCl/TiCl system.^[^ ^18^ ^]^ However, a major breakthrough came in 1972 when Fujishima and Honda demonstrated photoelectrochemical splitting of water into oxygen (O_2_) and H_2_ at a titanium dioxide (TiO_2_) electrode under visible light with no external voltage applied.^[^ ^19^ ^]^ Since then, many efforts have been made to develop a single photocatalyst for simultaneous reduction and oxidation, as shown in **Figure** **1**. In general, photocatalysts are wide bandgap semiconducting materials that absorb light with energy equal to or greater than the bandgap of the semiconductor. Ideally, the bandgap should be optimal to not only harvest the maximum amount of sunlight, but also have sufficient activation energy (overpotential) to drive the desired redox reaction. Mechanistically, upon light excitation, electrons move from the valence band to the conduction band, leaving a hole in the valence band bound by Coulombic attraction, known as an exciton.^[^ ^20^ ^]^ These excitons then either separate into free carriers or recombine via radiative or non‐radiative pathways. The photogenerated free carriers migrate to their respective catalytic sites for reduction and oxidation. To enable different types of photocatalytic chemical transformations with high selectivity, researchers have developed a plethora of photocatalysts using almost every single element of the periodic table. To date, inorganic metal oxides and nanoparticles have been used as photocatalysts, some of which have shown promising photocatalytic activity for water splitting,^[^ ^21^, ^22^ ^]^ pollutant degradation,^[^ ^23^, ^24^ ^]^ oxygen (O_2_) reduction reactions,^[^ ^25^ ^]^ organic transformations,^[^ ^26^, ^27^ ^]^ CO_2_ reduction,^[^ ^28^, ^29^, ^30^ ^]^ and NH_3_ synthesis.^[^ ^31^ ^]^ However, these inorganic materials are subject to common limitations, including scarcity of components, low tunability of band gap and band position, use of rare and expensive metals, and surface deactivation during the course of the reaction. For such photocatalysts, catalytic reactions mostly take place on the outer surface of the photocatalyst, which significantly affects the overall photocatalytic efficiency or quantum yield of the reaction due to surface deactivation, either by accumulation of the product as the reaction progresses or by surface oxidation of the catalytic active center. In addition, most of the photogenerated carriers recombine before reaching the catalytic site present on the surface.

Alternatively, inspired by natural photosynthesis, researchers have turned their attention to developing organic photocatalysts using abundant and inexpensive elements such as carbon (C), nitrogen (N), oxygen (O), silicon (Si), phosphorus (P) and sulfur (S).^[^ ^32^ ^]^ By covalently linking these elements using organic chemistry principles, it is theoretically possible to create an infinite number of organic semiconductors with different molecular structures, dimensionality, and tuneable optical and electronic properties, suitable for desired photocatalytic applications.^[^ ^33^ ^]^ The band gap and band position of organic semiconductors are relatively easy to modulate precisely by covalently linking π‐conjugated electron‐rich and electron‐deficient units in various permutations and combinations. Initially, various conjugated small molecules, oligomers, and polymeric organic semiconductors were used as photocatalysts for water splitting, pollutant degradation, organic transformations, and O_2_, CO_2_, and N_2_ reductions.^[^ ^34^ ^]^ Notably, polymeric carbon nitride (*g*‐CN), a 2D metal‐free photocatalyst, has shown promising photocatalytic activity for several chemical transformations.^[^ ^35^, ^36^ ^]^ The 2D planar and layered structure of *g*‐CN promotes light absorption, charge separation, and exciton/charge migration (Figure 1). However, challenges remain in tuning the band gap, increasing the surface area, and chemical functionalization of *g*‐CN, especially as these materials have to be prepared by thermal condensation of nitrogen‐rich molecules at high temperatures. Nevertheless, key findings from the studies on *g*‐CN have led to a number of insights and design principles for the development of next‐generation organic semiconductors as photocatalysts, which should combine the following features:

- A π‐conjugated molecular network for maximum light harvesting while maintaining the band gap and band position to provide sufficient overpotential for the desired chemical transformation.
- A dipolar chemical structure that can facilitate exciton dissociation and extended conjugation for charge migration to the respective catalytic sites.
- A highly accessible surface, which increases the catalytically active surface area and reduces the distance between the photosensitizer and the catalytic center, allowing the use of photogenerated charge carriers.
- A porous structure, in the best case, combining larger and smaller pores (hierarchical porosity) for simultaneous charge and mass diffusion.
- Covalent bonding to achieve conjugation and high photo‐ and chemical stability under the desired catalytic conditions.

Recognizing the importance of porous materials for photocatalysis, two general types of porous organic semiconductors have been developed in the last decade, conjugated microporous polymers (CMPs) and covalent organic frameworks (COFs). CMPs are covalently bonded amorphous polymers, whereas COFs are covalently bonded crystalline 2D or 3D materials.^[^ ^37^, ^38^ ^]^ Both can be prepared from pre‐designed building blocks and exhibit permanent porosity with high surface area. In general, CMPs are synthesized via metal‐catalyzed C─C coupling between the building blocks, whereas COFs are synthesized using dynamic covalent chemistry (DCC), which helps in error correction, leading to the formation of crystalline‐ordered frameworks.^[^ ^39^ ^]^ Due to the metal‐catalyzed synthesis of CMPs, residual metals can remain trapped in the polymer, which can even promote photocatalysis.^[^ ^40^ ^]^ More problematic, CMPs contain numerous defect or termination sites due to incomplete polymerization, which can act as traps for the photogenerated charge carriers. As a result, it is not always straightforward to establish a structure–property correlation for CMPs. In principle, COFs possess crystalline frameworks with minimal defects, and their topology and pore size can be tailored, which is a high asset to gain insights into structure‐photocatalytic performance relationships. It should be noted, that by far not all reported COFs are “highly” ordered and thus nearly defect‐free, but rather there is a fluid transition from crystalline frameworks (narrow reflections in XRD) to networks that just show a certain amount of order (broad reflections). To date, COFs are mainly used for photocatalytic H_2_ evolution^[^ ^41^ ^]^ along with organic transformations,^[^ ^42^, ^43^, ^44^ ^]^ dye degradation, hydrogen peroxide production,^[^ ^45^, ^46^ ^]^ CO_2_ reduction,^[^ ^47^ ^]^ O_2_ reduction,^[^ ^25^ ^]^ and N_2_ reduction.^[^ ^48^ ^]^

**Figure 2:**
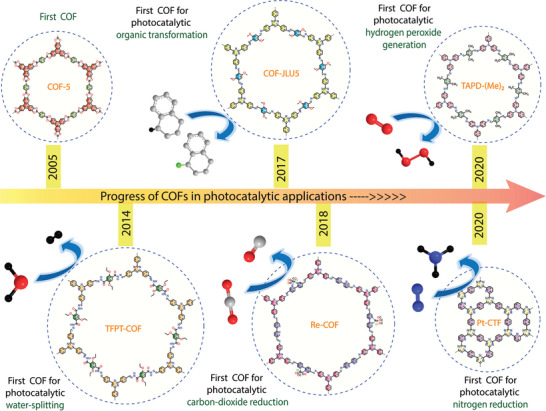
Progress of covalent organic frameworks in various photocatalytic applications, including water splitting, organic transformations, CO_2_ reduction, H_2_O_2_ generation, and nitrogen reduction.

While several review articles summarise the catalytic activity of COFs,^[^ ^49^, ^50^ ^]^ a critical perspective on the major advances made with COFs in photocatalysis is missing. In this review, we aim to provide a concise overview of the significant developments of COF photocatalysts in various applications while proposing unified design principles. We focus exclusively on the photocatalytic applications of COFs, including water splitting, organic transformations, CO_2_ reduction, H_2_O_2_ generation, and nitrogen reduction (**Figure** **2**). We also highlight the critical features of COFs that contribute to their photocatalytic performance. Finally, we provide insights into possible future developments of COF‐based heterogeneous catalysts for photocatalytic applications.

## 2 Advantages of COFs for Photocatalysis

COFs have gained significant attention in photocatalysis due to their crystallinity, permanent porosity, and efficient light absorption. The first COF was reported by Yaghi and co‐workers in 2005;^[^ ^51^ ^]^ however, it was not until 2014 that COFs were explored for photocatalytic applications. A significant breakthrough occurred in 2014 when Lotsch and co‐workers demonstrated photocatalytic hydrogen generation through proton reduction using a hydrazone‐based COF.^[^ ^52^ ^]^ Following this discovery, COFs have been extensively studied for various photocatalytic applications, including organic transformations, water oxidation, carbon dioxide reduction, hydrogen peroxide generation, and nitrogen reduction (Figure 2).

**Figure 3:**
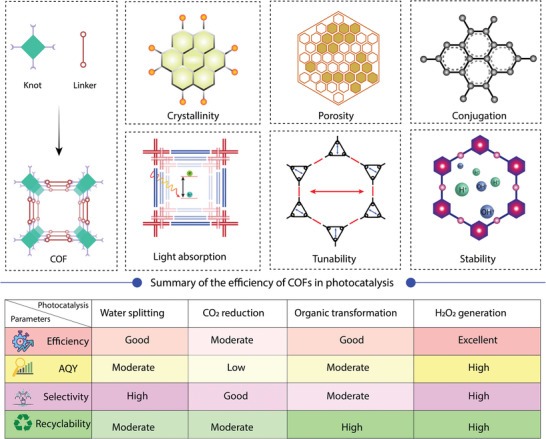
The key properties of COFs for photocatalytic applications and a summary of the efficiency of COFs in different types of photocatalysis.

There are a number of critical features of COFs that control their photocatalytic performance (**Figure** **3**), which are summarized as follows:

- **Crystalline framework**: A key property of COFs is their crystallinity, which distinguishes them from amorphous porous organic polymers (POPs). The crystalline framework allows the arrangement of the π‐conjugated building blocks in a periodic manner, where topology and stacking can be controlled through the molecular design. Stacking distance and the packing strongly affect the optical and electronic properties due to the change in orbital overlap in the ground and excited states. Unlike other organic polymers, predesigned bottom‐up synthesis of COFs helps to arrange the electron donor (D) and acceptor (A) in a periodic fashion, which facilitates the charge separation after photoexcitation and reduces charge recombination.^[^ ^53^ ^]^ Also, the long‐range periodicity of COFs in planar COF structures allows easy transport of photogenerated excitons or free charge carriers across the COF framework, minimizing charge recombination. In addition, the crystalline nature of COFs increases the kinetic energy and prolongs the lifetime of the charge carriers, which is critical in photocatalytic processes.^[^ ^54^ ^]^ COFs with a high degree of crystallinity can be constructed by carefully selecting the building blocks and optimizing the reaction conditions such as solvent, catalyst, temperature, and reaction time.^[^ ^55^ ^]^
- **Surface area**: The modular structure of COFs with tunable topology, symmetry, pore size, and connectivity can be designed by selecting different organic building blocks with different symmetry and molecular lengths.^[^ ^56^, ^57^ ^]^ Crystalline COFs have a high surface area with uniform pore size, which is essential to provide a large number of photoactive sites, sufficient space to accommodate active substrates, and efficient mass transport between catalytic sites, substrates, and sacrificial agents. The high surface area with a defined backbone structure also provides an ideal platform to immobilize and apply molecular catalysts or photosensitizers in heterogeneous photocatalysis.
- **Light absorption**: Light harvesting is an intrinsic property of COFs and is attributed to the extended π‐conjugation through the COF backbones. Various types of molecular photosensitizers can be incorporated in the COF as the building blocks, which can exhibit distinct optical properties compared to molecular analogs due to the extended conjugation and solid‐state packing. When a COF is exposed to light, electrons and holes can be generated and transferred to the valence and conduction bands, a crucial step for efficient photocatalysis.^[^ ^58^, ^59^ ^]^ COFs, consisting of repeating D‐A motifs, can modulate the optical properties due to charge transfer (CT) between the motifs with a profound effect on the light absorption capacity and band position.
- **Band position**: A photochemical reaction is possible when the energy of the absorbed photon is equal to or greater than the band gap of the photocatalyst. The photocatalytic activity of COF materials is directly influenced by their optical band position, which should generally be around 1.80 eV for the effective utilization of solar energy. In general, the optical band gap of COFs ranges from 1.50 eV to 3.0 eV, mostly centered near 2.20 eV due to the extended π‐conjugation.^[^ ^60^ ^]^ The optical energy levels of COFs can be tuned by incorporating extended aromatic units, donor‐acceptor units, and heteroatoms into the molecular building blocks or by changing the linkage and post‐synthetic modification of the COF backbone.^[^ ^61^ ^]^ A fundamental requirement for photocatalytic reactions is not only the band gap but also the band positions of the photocatalysts. The band gap and band position of the COFs can be altered by functionalizing the COFs and changing the linkages, thereby enhancing the photoexcitation and charge separation process.
- **Charge separation and transport**: Charge separation and transport in COFs are crucial for increasing their photocatalytic efficiency. The exciton binding energy in COFs determines how easily excitons can be separated into free charge carriers. Efficient exciton migration within the COF structure ensures that these excitons reach the active sites before recombination occurs. Effective charge separation and reduced recombination are essential to maximize the generation of free charges for catalytic reactions. Charge transport within COFs allows the movement of these charges to the reaction sites where they participate in photocatalytic processes.^[^ ^62^, ^63^ ^]^ In this respect, the formation of heterojunctions, protonation, or the presence of an internal electric field within COFs can significantly enhance charge separation. Heterojunctions create an interface between materials with different band orientations, promoting the separation of electrons and holes. Protonation modifies the electronic structure and increases charge mobility, while an internal electric field drives the charge carrier in opposite directions, reducing recombination and improving overall photocatalytic performance.
- **Photochemical stability**: Another important property to be considered in the strategic design of photocatalysts is photostability and chemical stability. From a reusability perspective, long‐term stability and tolerance to various agents, including acids, bases, oxidants, cocatalysts, and sacrificial agents, are essential for potential catalysts. The stability of COFs can be achieved through the selection of building blocks and linkages that ensure sustained catalytic performance, making them potential candidates for various photocatalytic reactions.^[^ ^64^, ^65^ ^]^ In addition, COFs provide inherent stability against photobleaching and photodegradation. Sometimes, protonation or post‐synthetic modification of COFs has also been shown to improve their photochemical stability.

Indeed, the photocatalytic performance is controlled by multiple effects rather than a single variable. Therefore, in order to improve the photocatalytic performance of COFs, several strategies, such as precise selection of building blocks,^[^ ^66^ ^]^ variation of linkages in the backbone of the COF,^[^ ^67^ ^]^ structural transformation of COFs by post‐synthetic modification,^[^ ^68^ ^]^ integration of photosensitizing units, immobilization of cocatalyst,^[^ ^69^ ^]^ modification of reaction conditions of COF synthesis (by changing solvent, catalyst, reaction temperature and time) have been adopted to regulate the structure of COFs.^[^ ^54^ ^]^ However, it must also be stated that it is almost impossible to change just one variable in a COF to analyze its influence on catalytic performance. For example, if you change just one building block of the COF, you may not only change its electron–donor or acceptor strength, but also influence pore structure and size, the surface area, the polarity, and probably many factors, which influence the COF properties and performance.

## 3 General Mechanisms of Photocatalysis using COFs as Photocatalysts

A photocatalyst accelerates a chemical reaction when exposed to light by decreasing the activation energy, the process is known as photocatalysis. Inspired by the natural process of photosynthesis, researchers are on a quest for the development of efficient photocatalysts to mimic the photosynthetic process, that is, to convert solar energy into chemical energy. Interestingly, the band gap, the conduction band, and the valence band of COF‐based catalysts can be precisely tuned to suit a variety of photocatalytic applications. The well‐established solid‐state band theory can explain the fundamental photocatalytic mechanism of COFs. This theory divides the process into three key steps:

- Upon irradiation with light exceeding the band gap energy, an electron in the valence band of COFs absorbs a photon and becomes excited. This excitation process promotes the electron to the conduction band, leaving behind a hole in the valence band.
- The photogenerated electron and hole then migrate across the material to the surface of the catalyst. However, during this diffusion process, a portion of these electron‐hole pairs recombine, reducing their overall efficiency.
- The capture and reaction of the photogenerated electrons and holes by the catalyst or cocatalysts to facilitate the redox reactions.

**Figure 4:**
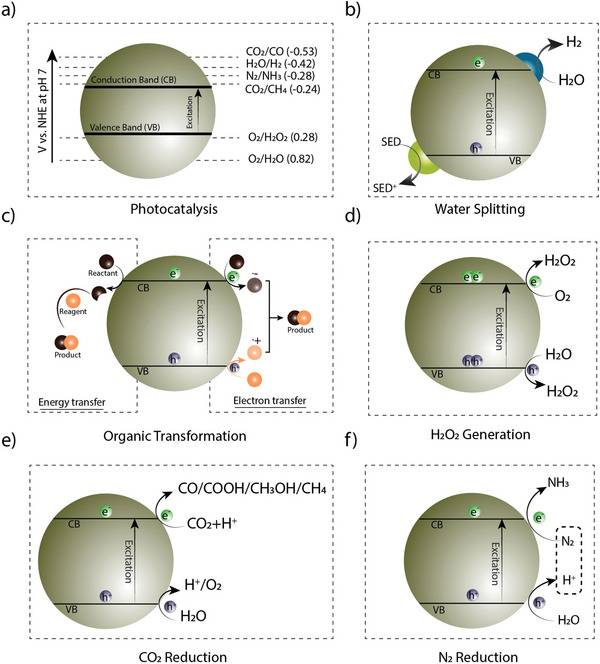
Schematic representation of the general mechanism of the different photocatalytic reactions such as water splitting, organic transformation, hydrogen peroxide generation, carbon dioxide reduction, and nitrogen reduction.

In photocatalysis using COFs, photogenerated free carriers (electrons and holes) migrate to catalytic sites where reduction and oxidation reactions by electron and hole, respectively, can take place. The efficiency of these processes depends on the ability of the COFs to photogenerate free charge carriers and transport them to the appropriate catalytic site. Several factors, such as band position, pH, and catalytic sites, are primarily responsible for the regulation of oxidation and reduction reactions in COFs. For reduction reactions, the conduction band of the COF must be positioned lower (more negative) than the reduction potential of the target molecule to ensure that the photogenerated electrons have sufficient energy to drive the reduction. In the case of an oxidation reaction, the valence band must be positioned higher (more positive) than the oxidation potential of the target molecule, indicating that the photogenerated holes have sufficient oxidative power to drive the oxidation process. The rate‐limiting steps of photocatalysis in different applications are summarised in Table 1 and **Figure** **4**a.

**Table 1:** The summary of rate‐limiting steps of photocatalysis in different applications.

Photocatalysis	Key redox process	Rate‐limiting step
Hydrogen generation	Reduction of protons: 2H⁺ + 2e⁻ → H₂	Reduction of protons to hydrogen molecules via a two‐electron process
Oxygen evolution	Oxidation of water: 2H₂O → O₂ + 4H⁺ + 4e⁻	Oxygen evolution reaction via a four‐electron process
Overall water splitting	Oxidation water: 2H₂O → O₂ + 4H⁺ + 4e⁻	Oxygen evolution reaction via a four‐electron process
CO₂ reduction	Reduction of CO_2_: CO₂ + 2H⁺ + 2e⁻ → CO (or other products like CH₄, CH₃OH, etc.)	CO₂ activation and multi‐electron reduction
Organic transformations	Selective oxidation/reduction	Hole or electron transfer to organic substrates, intermediate stability
H_2_O_2_ generation	Reduction of oxygen: O_2_ + 2H^+^ + 2e^‐^ → H_2_O_2_ Oxidation of water: H_2_O → H_2_O_2_ + 2H^+^ + 2e^–^	ORR and WOR via a two‐electron process
Nitrogen reduction	Reduction of nitrogen: N_2_ + 6H^+^ + 6e^‐^ → 2NH_3_	N_2_ activation and selective proton/electron transfer via six‐electron process.

For instance, in photocatalytic water splitting, an oxidative catalyst can promote the oxidation of water molecules (H_2_O) at its active sites, generating O_2_ molecules and protons (H^+^ ions).^[^ ^70^ ^]^ Conversely, the reduction of H^+^ to H_2_ gas can be driven by a reductive catalyst (Figure 4b). In general, the process of overall water splitting (OWS) is suppressed due to an unwanted reverse reaction. Heterojunction photocatalysts, constructed by combining COFs with other materials, offer a solution to this challenge. These composites exhibit enhanced photocatalytic efficiency due to the formation of internal electric fields at the junctions between the COF and the other components, which facilitate the exciton separation and transport of free charge carriers to respective catalytic sites. These fields effectively separate and direct photogenerated charge carriers toward the interface, promoting the desired hydrogen evolution and oxygen evolution reactions. Several well‐established heterojunction configurations exist, including Type‐II, Z‐scheme, and S‐scheme. Among these, Type‐II and Z‐scheme photocatalysts have shown exceptional promise in enhancing the activity of the hydrogen evolution reaction.^[^ ^71^ ^]^

In the indirect energy‐mediated pathway of photocatalytic organic conversion reactions, the substrate is then converted to a more reactive species that subsequently reacts with another substrate. On the other hand, in the direct electron transfer pathway, electrons are transferred between the electrophile, nucleophile, and metal substrates (Figure 4c). Photocatalysis can be also used to reduce molecular oxygen to the superoxide radical anion (^•^O_2_¯) by single electron transfer (SET). The ^•^O_2_¯, in turn, can oxidize the substrates to the radical cation, leading to its subsequent functionalization to form the desired products.^[^ ^72^ ^]^ Remarkably, sunlight can initiate the electron transitions necessary to overcome energy barriers in these redox processes without the need for thermal energy. Consequently, light‐driven organic transformations can be achieved under milder reaction conditions than conventional catalytic methods.

Typically, H_2_O_2_ can be produced via oxygen reduction reaction (ORR) and water oxidation reaction (WOR) pathways,^[^ ^45^ ^]^ often involving different reaction pathways described by the number of electrons/holes transferred (Figure 4d). In the ORR pathway, H_2_O_2_ can be produced either via a direct 2e^−^ reduction or a step‐wise one‐electron 1e^−^ oxygen reduction. In the direct ORR process, O_2_ directly undergoes a 2e^−^ photoreduction by reacting with 2H^+^ to produce H_2_O_2_. In the step‐wise ORR route, O_2_ is first reduced by 1e^−^ to generate superoxide radical (O_2_^•−^), which reacts with H^+^ to form the HO_2_^•−^ radical. Further, the reduction of the HO_2_^•−^ radical by another electron generates the HO_2_^−^ anion, which finally reacts with H^+^ ions to produce the desired product of H_2_O_2_. On the other hand, in the WOR path, H_2_O_2_ can also be produced via a direct or step‐wise oxidation process. In the direct‐step WOR path, the H_2_O molecule is directedly oxidized by two photogenerated holes (h^+^) to produce H_2_O_2_. Meanwhile, in the step‐wise WOR path, H_2_O is first oxidized by 1h^+^ to form hydroxyl radical (^•^OH) intermediates, which are immediately coupled with another ^•^OH of opposite spin to generate the H_2_O_2_. In addition, the four‐electron (4e^−^) WOR pathway, commonly observed in photocatalytic overall water splitting, produces oxygen (H_2_O/O_2_; +1.23 V vs NHE at pH 0), instead of hydrogen peroxide (H_2_O/H_2_O_2_; +1.76 V vs NHE, pH 0).^[^ ^73^ ^]^ Hence, the formation of O_2_ via the 4e^−^ WOR process is relatively straightforward compared to the formation of H_2_O_2_ via the 2e^−^ WOR process. In the WOR pathway towards H_2_O_2_ generation, the generated O_2_ molecules can participate in the production of H_2_O_2_ via the 4e^−^–2e^−^ cascaded process (H_2_O→O_2_→H_2_O_2_), which could be more thermodynamically favorable and also can avoid the catalyst poisoning. It is worth noting that photocatalytic H_2_O_2_ generation by COF‐based photocatalysts has often occurred via the oxygen reduction reaction process — a more favorable pathway for H_2_O_2_ generation.

Effective CO₂ reduction requires a COF photocatalyst with active sites that selectively reduce CO₂ over protons or the reaction environment should minimize competing reactions such as hydrogen evolution. The mechanism of CO₂ reduction to value‐added products using COFs as photocatalysts involves several key steps (Figure 4e):^[^ ^28^, ^74^ ^]^

- **Light absorption and exciton separation**: When exposed to light, the COF photocatalyst absorbs photons and generates excitons (electron‐hole pairs) within its π‐conjugated structure, which are separated into free electrons and holes.
- **CO₂ adsorption**: CO₂ molecules are adsorbed onto the active sites of the COF, typically onto metal centers or functional groups designed for CO₂ capture.
- **Electron transfer**: The photogenerated electrons are transferred to the adsorbed CO₂ molecules, reducing CO₂ to various value‐added products such as CO, CH₄, CH₃OH, or formic acid, depending on the reaction conditions and the specific COF structure.

CO_2_ reduction has a reduction potential similar to that of proton reduction (hydrogen evolution); therefore, to avoid competing proton reduction consuming the photogenerated electrons and reducing the efficiency of CO₂ reduction, strategies such as designing with specific active sites or cocatalysts that preferentially bind CO₂ over protons are used. In addition, reaction conditions such as pH and solvent environment can be optimized to suppress proton reduction and favor CO₂ reduction. Finally, the incorporation of cocatalysts or the formation of heterojunctions within the COF has been found to facilitate selective CO₂ reduction by creating an energy barrier or kinetic preference against proton reduction. Compared to thermo‐catalysis or electrocatalysis, photocatalysis offers a more convenient, cost‐effective, and eco‐friendly approach to chemical conversion under atmospheric conditions. Similarly, in photocatalytic N_2_ reduction, photogenerated holes then oxidize the water to form O_2_, while the photogenerated electrons reduce the N_2_ to form NH_3_ (Figure 4f).

## 4 Photocatalytic Applications of COFs

### 4.1 Photocatalytic Water Splitting

Water splitting is crucial for the sustainable production of hydrogen and oxygen as a clean energy source and is essential for several industrial processes. Current technologies face limitations such as high energy consumption, slow reaction kinetics and the need for expensive catalysts. COFs offer a promising alternative as these materials often exhibit band gaps and positions that are principally suited for photocatalytic water splitting. The following section summarizes the applications of COFs for both half‐reactions of water splitting, namely hydrogen generation and oxygen evolution as well as overall water splitting:

#### 4.1.1 Photocatalytic H_2_ Generation

**Figure 5:**
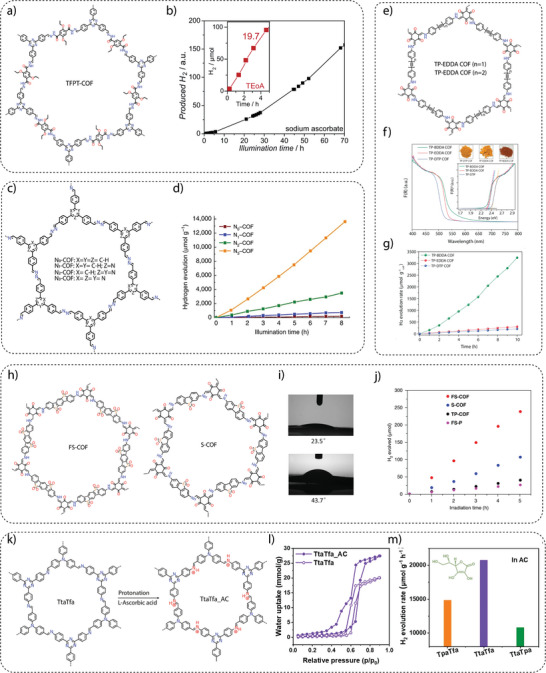
a) Chemical structure of TFPT‐COF, and b) photocatalytic hydrogen generation using TFPT‐COF. Reproduced with permission.^[^ ^52^ ^]^ Copyright 2014, Royal Society of Chemistry. c) Structures of N_X_‐COFs, and d) hydrogen generation performance of N_X_‐COFs with varying degrees of nitrogen atom substitution. Reproduced with permission.^[^ ^75^ ^]^ Copyright 2015, Springer Nature. e) Synthesis of acetylene‐based TP‐EDDA (*n* = 1) and diacetylene‐based TP‐BDDA (*n* = 2) COFs. f) Band gap measurement and g) hydrogen evolution performance of TP‐EDDA and TP‐BDDA COFs. Reproduced with permission.^[^ ^66^ ^]^ Copyright 2018, American Chemical Society. h) The structures of sulfone‐based FS‐COF (fused sulfone) and S‐COF. i) Contact angle measurement of FS‐COF (top) and S‐COF (bottom). j) Hydrogen evolution performance of FS‐COF and S‐COF. Reproduced with permission.^[^ ^81^ ^]^ Copyright 2018, Springer Nature. k) The scheme of protonation of TtaTfa_AC COF. l) Water sorption analyses of TtaTfa_AC COF show better water wettability behavior than that of pristine TtaTfa COF. m) Hydrogen generation performance using l‐ascorbic acid as a sacrificial agent. Reproduced with permission.^[^ ^83^ ^]^ Copyright 2021, Wiley‐VCH.

Hydrogen generation from water involves a reductant, also called a sacrificial electron donor (SED), which drives the overall reaction instead of water oxidation. In 2014, Lotsch and co‐workers achieved a breakthrough in photocatalytic hydrogen generation using a hydrazone‐based COF (TFPT‐COF),^[^ ^52^ ^]^ synthesized via polycondensation between 4,4′,4″‐tri(p‐formyl phenyl)‐1,3,5‐triazine (TFPT) and 2,5‐diethoxyterephthalohydrazide (DETH) (**Figure** **5**a). The resulting TFPT‐COF exhibited a high surface area and suitable band gap to promote photocatalytic water splitting. Indeed, the COF demonstrated good activity for the hydrogen evolution reaction rate (HER) up to 1970 µmol g^−1^ h^−1^ in the presence of triethanolamine as a SED (Figure 5b). Building upon their pioneering work, the same group delved deeper into the design and engineering of COFs for photocatalytic applications. Their approach involved the use of hydrazine and central triphenylene aldehydes with varying degrees of nitrogen atom substitution (N_x_‐COF where X = 0, 1, 2, 3) (Figure 5c).^[^ ^75^ ^]^ Nitrogen atoms substituting carbon, moving from benzene (N_0_) to triazine (N_3_) functionalities, lead to a higher planarity of the molecules. This planarity improvement enhances the conjugation and thus affects the light absorption and charge transport properties of the COFs and, ultimately, their photocatalytic performance. N_0_‐COF, without nitrogen substitution, showed the lowest activity with a hydrogen evolution rate of 23 µmol h^−1^ g^−1^, whereas N_3_‐COF, with the highest nitrogen content, showed a remarkable 74‐fold increase in activity (1703 µmol h^−1^ g^−1^) (Figure 5d). After this encouraging work, several groups investigated further strategies to increase hydrogen generation and solar‐to‐chemical energy conversion of COFs,^[^ ^76^ ^]^ which are summarized below:

a) **Conjugation**: The extended π‐conjugation within the COF framework plays a decisive role in various properties, such as light absorption, band gap, charge transfer, conductivity, etc. In recent years, the synthesis of COFs using a variety of building units, including phenyl, biphenyl, naphthalene, anthracene, pyrene, and heteronuclear molecular functionalities (triazine‐ or heptazine‐based moieties), has been extensively explored. To investigate the impact of conjugation on water‐splitting activity without introducing heteronuclear molecular functionalities, acetylene (−C≡C−)‐ and diacetylene (−C≡C−C≡C−)‐linked COFs were synthesized through an acid‐catalyzed solvothermal reaction between 1,3,5‐triformylphloroglucinol (TP) and either 4,4′‐(ethyne‐1,2‐diyl)dianiline (EDDA) or 4,4′‐(buta‐1,3‐diyne‐1,4‐diyl)dianiline (BDDA) (Figure 5e).^[^ ^66^ ^]^ To compare the effect of linear conjugation on photocatalytic activity, TP‐DTP COF (DTP: 4,4″‐diamino‐p‐terphenyl), a reference isoreticular COF, was synthesized. Diffuse reflectance ultraviolet‐visible (UV‐vis) spectra show a significant red shift in absorbance for diacetylene‐containing TP‐BDDA as compared to acetylene‐linked TP‐EDDA and terphenyl‐based TP‐DTP COFs (Figure 5f). In photocatalytic hydrogen evolution, TP‐BDDA, with its comparatively better conjugation, achieved a hydrogen evolution rate of 324 ± 10 µmol g^−1^ h^−1^ over 10 h. In contrast, TP‐EDDA and TP‐DTP showed a lower hydrogen evolution rate of 30 ± 5 µmol g^−1^ h^−1^ and 20 ± 5 µmol g^−1^ h^−1^, respectively (Figure 5g). These results indicate that the conjugated diacetylene moiety is key to improving photocatalytic hydrogen generation performance. In addition to lowering the band gap, the diacetylene moieties are responsible for high charge career mobility, i.e., easy migration of photogenerated excitons to the surface of the photocatalyst.

A similar observation was made using a series of π‐conjugated COFs containing the same donor‐acceptor structure but linked via either cyanovinylene‐ or imine‐moieties, respectively.^[^ ^77^ ^]^ The cyanovinylene‐linked COF exhibited even overall photocatalytic water splitting performance, reaching an apparent quantum efficiency of 2.53% (420 nm), while a similar imine‐linked COF didn't show any activity in water splitting, indicating the role of conjugation in modulating the band structure and promoting charge separation in COFs.

b) **Hydrophilicity**: The primary mechanism governing photocatalytic reactions involves the adsorption of reactant molecules onto the surface of the photocatalyst.^[^ ^78^, ^79^, ^80^ ^]^ Engineering the catalyst surface is thus a critical strategy for controlling catalytic activity, selectivity, and stability, as the catalyst surface serves as the interface where reactant adsorption, intermediate formation, and product desorption occur. A hydrophilic surface attracts hydrophilic reactants to the active site, while a hydrophobic surface facilitates the accumulation of hydrophobic reactants. In this context, the incorporation of sulfone moieties into the COF structure has been shown to result in a remarkable improvement in hydrogen generation efficiency, which is likely due to the improved wettability and favorable interactions with water molecules, facilitating their adsorption onto the surface of COFs.

For example, two COFs were synthesized using 1,3,5‐triformylphloroglucinol and either 3,7‐diaminodibenzo[*b*,*d*]thiophene sulfone (S‐COF) or 3,9‐diamino‐benzo[1,2‐*b*:4,5‐*b*′]bis[1]benzothiophene sulfone (FS‐COF), respectively (Figure 5h).^[^ ^81^ ^]^ The contact angle measurements showed that the FS‐COF has a higher water wettability in comparison to S‐COF (Figure 5i). FS‐COF demonstrates impressive photocatalytic hydrogen generation activity (10100 µmol g^−1^ h^−1^) compared to S‐COF (4440 µmol g^−1^ h^−1^) (Figure 5j). This remarkable difference in performance is attributed to the enhanced water wettability of FS‐COF due to the presence of two sulfone units. Also, the photocatalytic hydrogen evolution activity of the S‐COF and FS‐COF significantly surpassed their semicrystalline polymer analogs, S‐COF‐P7 and FS‐COF‐P10, respectively, which was attributed to the superior charge carrier transport facilitated by the long‐range periodicity of the crystalline COFs. Recently, zwitterionic COFs have been presented, characterized by the presence of both cationic and anionic groups. ZVCOF‐1 showed enhanced exciton generation and hydrophilicity, resulting in an impressive hydrogen evolution rate of 2052 µmol h^−1^ when 20 mg of catalyst are applied and an outstanding apparent quantum yield (AQY) of 47.1% at 420 nm.^[^ ^82^ ^]^ These results demonstrate the role of wettability in enhancing photocatalytic hydrogen generation from water.

c) **Protonation**: In traditional photocatalytic hydrogen generation, triethanolamine (TEOA) is commonly used as a SED. However, recent studies have shown that ascorbic acid can exhibit higher hydrogen evolution rates (HER) when used as a SED, especially when imine‐linked COFs are tested. Critical analyses have shown that imine bonds within the COF structure are susceptible to protonation in ascorbic acid, which leads to significant changes in charge and optical properties. Protonation increases the hydrophilicity, which improves water accessibility in the COF backbone, and planarity, which leads to better π‐conjugation, resulting in lower band gaps, extended light absorption, and improved carrier mobility and separation efficiency. A representative example is the imine‐based protonated TtaTfa COF, which is constructed using the acceptor  2,4,6‐Tris(4‐aminophenyl)triazine (Tta) and donor Tris(4‐formyl phenyl)amine (Tfa) moieties (Figure 5k), which displayed an impressive photocatalytic hydrogen evolution reaction (20700 µmol g^−1^ h^−1^) performance when ascorbic acid has been used as a sacrificial electron donor (Figure 5m).^[^ ^83^ ^]^ Upon treatment with ascorbic acid (AC), the pristine COFs undergo a dramatic color change from bright yellow to deep red. This transition was accompanied by increased planarity (theoretically proven), leading to a higher degree of conjugation. UV–Vis spectroscopy confirmed the change, revealing an absorption onset shift from 507 nm to 688 nm after protonation. The protonated COF also showed a reduced band gap, enhanced light absorption, and improved charge carrier mobility and separation efficiency. Furthermore, the increased water accessibility within the COF pores contributes to improved performance as a water‐splitting photocatalyst (Figure 5l).

In similar studies, CTF‐1, synthesized by heating a mixture of 1,4‐dicyanobenzene (DCB) and trifluoromethanesulfonic acid at 250 °C, was first exfoliated and then protonated by sequential treatments with ammonium persulfate and phosphoric acid.^[^ ^84^ ^]^ Photocatalytic hydrogen evolution studies revealed that the protonated CTFs achieved a hydrogen evolution rate of 6595 µmol g^−1^ h^−1^, which is 7.14 times higher than that of the pristine CTFs. This enhancement could be attributed to protonation, which improves the interaction between the CTF, water molecules, and the Pt cocatalyst, increasing the water concentration around the active catalytic sites and facilitating the transfer of photogenerated electrons from the Pt cocatalyst to the water. These results highlight the important role of protonation in enhancing the performance of photocatalytic hydrogen evolution.

**Figure 6:**
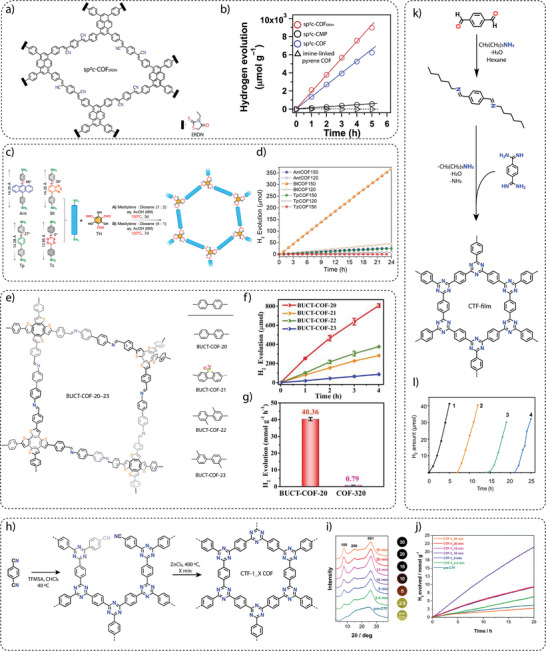
a) Vinylene linked sp^2^‐COF_ERDN_, and b) comparison of hydrogen evolution rates between sp^2^‐COF_ERDN_ and pristine sp^2^‐COF. Reproduced with permission**.^[^** ^86^ **^]^** Copyright 2019, Elsevier. c) Scheme for the synthesis of different COFs with comparable lengths but different torsional angles prepared under different solvothermal reaction conditions. d) Photocatalytic hydrogen generation analyses showing better activity for BtCOF150. Reproduced with permission.^[^ ^91^ ^]^ Copyright 2020, American Chemical Society. e) The structures of BUCT‐COFs, and f) their hydrogen evolution performance. g) The photocatalytic hydrogen generation performance of conjugated BUCT‐20 as compared to non‐conjugated three‐dimensional COF‐320. Reproduced with permission.^[^ ^92^ ^]^ Copyright 2024, Wiley‐VCH. h) Synthesis of CTF‐ 1_X COF (X = 2.5, 5, 10, 20, 30 min). i) PXRD pattern showing the crystallinity of COFs. j) Hydrogen generation using CTF‐1_X COFs. Reproduced with permission.^[^ ^94^ ^]^ Copyright 2017, Royal Society of Chemistry. k) Synthesis of CTF film using interfacial method. l) Hydrogen evolution performance using CTF film over time. Reproduced with permission.^[^ ^95^ ^]^ Copyright 2021, Spring Nature.

d) **Molecular heterojunction**: Defects in COF structures can act as sites for charge recombination. To mitigate these problems, end‐capping can be used to introduce terminal moieties at the edges of COF fragments (crystallites), which can regulate the optoelectronic properties of COFs and improve the activity for photocatalytic hydrogen generation. To demonstrate the effect of end‐capping on photocatalytic hydrogen generation, a vinylene‐linked sp^2^c COF was prepared by polycondensation of 1,3,6,8‐tetrakis(4‐formylphenyl)pyrene (TFPPy) and 1,4‐phenylenediacetonitrile (PDAN) linkers under solvothermal conditions.^[^ ^85^ ^]^ For comparison, the synthesis of end‐capped sp^2^c‐COF_ERDN_ was achieved by a three‐component polycondensation of TFPPy, PDAN and 3‐ethylrhodanine (ERDN) in a molar ratio of 1/1.95/0.05 (**Figure** **6**a).^[^ ^86^ ^]^ The integration of electron‐deficient ERDN units at the edges of the COF significantly affected its electronic properties, in particular the frontier orbitals, the band structure and the interface between the COF and other materials. sp^2^c‐COF_ERDN_ showed a photocatalytic activity of 2120 µmol g^−1^ h^−1^, which was 1.6 times higher compared to the non‐functionalized sp^2^c‐COF (1360 µmol h^−1^ g^−1^) (Figure 6b), demonstrating the suitability of molecular heterojunction for the development of various photocatalysts. In addition to purely organic heterojunction strategies, the formation of COF‐based heterojunctions using MOFs and inorganic and organic materials has been attempted to improve photocatalytic hydrogen generation performance.^[^ ^87^, ^88^, ^89^, ^90^ ^]^

e) **Charge separation and transport**: Efficient charge separation and transport is an important parameter for photocatalysis. To validate the charge separation and transport properties of COFs, a series of isoreticular two‐dimensional (2D) COFs was synthesized by carefully engineering the building blocks to systematically vary factors such as torsion angles, molecular lengths, donor‐acceptor conjugation and reaction conditions. The synthesis of *β*‐ketoenamine‐linked isoreticular COFs, namely TpCOF, AntCOF, BtCOF and TzCOF, was achieved by the reaction of 1,3,5‐triformylphloroglucinol with [1,1′: 4′,1″‐terphenyl]‐4,4″‐diamine (Tp), 4,4′‐(anthracene‐9,10‐diyl)dianiline (Ant), 4,4′‐(benzo[c][1,2,5]thiadiazole‐4,7‐diyl)dianiline (Bt) and 4, 4′‐(1,2,4,5‐tetrazine‐3,6‐diyl)dianiline (Tz) linkers, respectively, under solvothermal conditions (Figure 6c).^[^ ^91^ ^]^

In particular, the electron‐deficient benzothiadiazole (Bt)‐based COF, BtCOF150, exhibited a promising hydrogen evolution rate of 750 ± 25 µmol g^−1^ h^−1^ under visible light irradiation. On the other hand, the tetrazine (Tz)‐based COF, TzCOF, showed no hydrogen evolution activity despite its broad light absorption, large surface area, and high crystallinity (Figure 6d). This suggests that the electronic structure of the Tz unit might be less favorable for facilitating the necessary charge transfer processes. It was also observed that AA′‐stacked COFs exhibited higher photocatalytic hydrogen generation activity than AB and ABC‐stacked COFs, which was attributed to the significantly improved exciton migration and charge transport properties in the AA′‐stacked COFs. The systematic investigation showed that the interplay between structure, properties, and activity are the main factors influencing the photocatalytic performance of COFs.

To date, mostly 2D COFs have been used for photocatalytic water splitting due to their crystallinity, porosity, conjugated structures, and light absorption in the visible region. In contrast, photocatalytic hydrogen generation using three‐dimensional (3D) COFs is scarcely reported as they are often not fully π‐conjugated frameworks due to sp^3^ carbon‐based tetrahedral building blocks. On the other hand, 3D COFs might offer several advantages over 2D COFs, such as increased surface area, more accessible active sites, and an interconnected porous network in three dimensions, which facilitates charge separation and transport.

Therefore, a series of fully conjugated three‐dimensional COFs have been developed, designated BUCT‐COF‐20, ‐21, ‐22 and ‐23, using a saddle‐shaped cyclooctatetrathiophene derivative COThP‐CHO as building block connected into a framework via benzidine (BD), 3,7‐diaminodibenzo[b,d]thiophene‐5,5‐dioxide (SA), 2,2′‐dimethy[1,1′‐biphenyl]‐4,4′‐diamine (MBD) and 3,3′‐dimethylbiphenyl‐4,4′‐diamine (DMB), respectively (Figure 6e).^[^ ^92^ ^]^ Among these series of COFs, non‐substituted benzidine‐based BUCT‐COF‐20 exhibited the highest hydrogen evolution rate of 40360 µmol g^−1^ h^−1^ under visible light irradiation in 0.1 M ascorbic acid and in the presence of Pt cocatalyst (Figure 6f). The photocurrent intensity and average fluorescence lifetimes showed that BUCT‐COF‐20 exhibited better photoresponse and photogenerated carrier availability, followed by BUCT‐COF‐22, BUCT‐COF‐21, and BUCT‐COF‐23. This superiority in hydrogen generation is attributed to their π‐extended three‐dimensional structures and ordered donor‐acceptor (D‐A) configurations, which effectively facilitate charge transfer and separation. In contrast, a non‐conjugated three‐dimensional COF‐320 synthesized through the solvothermal reaction of sp^3^ carbon‐based tetrahedral linker 4,4′,4″,4‴methanetetrayltetraaniline and benzidine exhibited a significantly lower hydrogen evolution rate (790 µmol g^−1^ h^−1^) (Figure 6g). By providing efficient channels for charge transport, these results highlight the importance of fully π‐conjugated three‐dimensional COFs in photocatalysis.

Although various COFs synthesized with different linker‐linkage combinations have been investigated for photocatalytic hydrogen generation, covalent triazine frameworks (CTFs) – a class of porous organic materials formed by the trimerization of nitriles – have attracted considerable attention. This is due to their high surface area, tunable pore structure, high nitrogen content, and exceptional chemical stability. The initial approach for synthesizing CTFs required a high temperature (400 °C) and resulted in low crystallinity.^[^ ^93^ ^]^ However, the perfect balance of temperature and time could help to increase the crystallinity of CTFs, resulting in the over‐carbonization of the material. To address the challenges of using CTFs for photocatalysis, we attempted to synthesize CTFs by combining two steps. In the first step, a pre‐CTF was prepared by reacting 1,4‐dicyanobenzene in CHCl_3_ and trifluoromethanesulfonic acid at 40 °C. In the second step, the pre‐CTF was thoroughly mixed with ZnCl_2_ in a molar ratio of 1:0.8 and heated in a preheated oven held at 400 °C for 2.5, 5, 10, 15, 20, and 30 min to prepare the CTF‐1_X materials (where X is the reaction time in minutes) by reducing the reaction time from 40 h to as little as 30 min (Figure 6h).^[^ ^94^ ^]^ In particular, CTF‐1_10, synthesized with a shortened reaction time of only 10 minutes, showed the optimum balance of crystallinity and catalytic activity for photocatalytic hydrogen evolution under visible light irradiation with a HER of 1072 µmol g^−1^ h^−1^ (Figure 6i,j). These results demonstrated the utility of a fast and facile route using CTFs for photocatalytic hydrogen production.

Compared to their suspended counterparts, films made of CTFs offer several advantages, such as improved light harvesting, simplified recovery, and scalability. Recently, a practical and efficient method for the preparation of free‐standing semicrystalline CTF films with impressive lateral dimensions of up to 250 cm^2^ and controllable thickness (30 to 500 nm) has been demonstrated.^[^ ^95^ ^]^ Typically, CTF films have been prepared by first synthesizing an imine precursor by reacting aldehyde and *n*‐hexylamine in hexane. Due to the weak polarity of the long carbon chain, the imine precursor floats on the surface of the DMSO layer to form an interface. In the second step, amidine monomer and Cs_2_CO_3_ were dissolved in DMSO, and then imine precursor was added dropwise on the surface of the DMSO layer while keeping the temperature at 60 °C for 30 min to evaporate hexane, followed by heating at 100 °C for 72 h (Figure 6k). The photocatalytic hydrogen evolution performance of CTF film investigated under visible light (>420 nm) with Pt NPs as cocatalyst and triethanolamine as a sacrificial agent showed that the CTF films immobilized on glass support exhibited promising activity with a hydrogen evolution rate of 5400 µmol h^−1^ m^−2^(Figure 6l). Interestingly, the photocatalytic experiment showed that the film exhibited a stable hydrogen evolution rate of more than 60 h, demonstrating high recyclability. This report represents a significant development in the overcoming of the processability of COF and CTF films for use in photocatalysis.

#### 4.1.2 Photocatalytic Oxygen Evolution

The oxygen evolution reaction (OER) is the rate‐determining step in the overall photocatalytic water splitting due to the complex four‐electron reaction (2H₂O → O₂ + 4H⁺ + 4e⁻). Pristine, COFs typically don't show overall water splitting due to their limited ability to effectively separate and transport photogenerated charge carriers (electrons and holes). Without metal cocatalysts, which often facilitate critical redox reactions, these COFs struggle to efficiently catalyze both the hydrogen and oxygen evolution reactions. Hence, the incorporation of molecular cocatalysts into COFs is known to be a promising strategy for enhancing photocatalytic water splitting.

**Figure 7:**
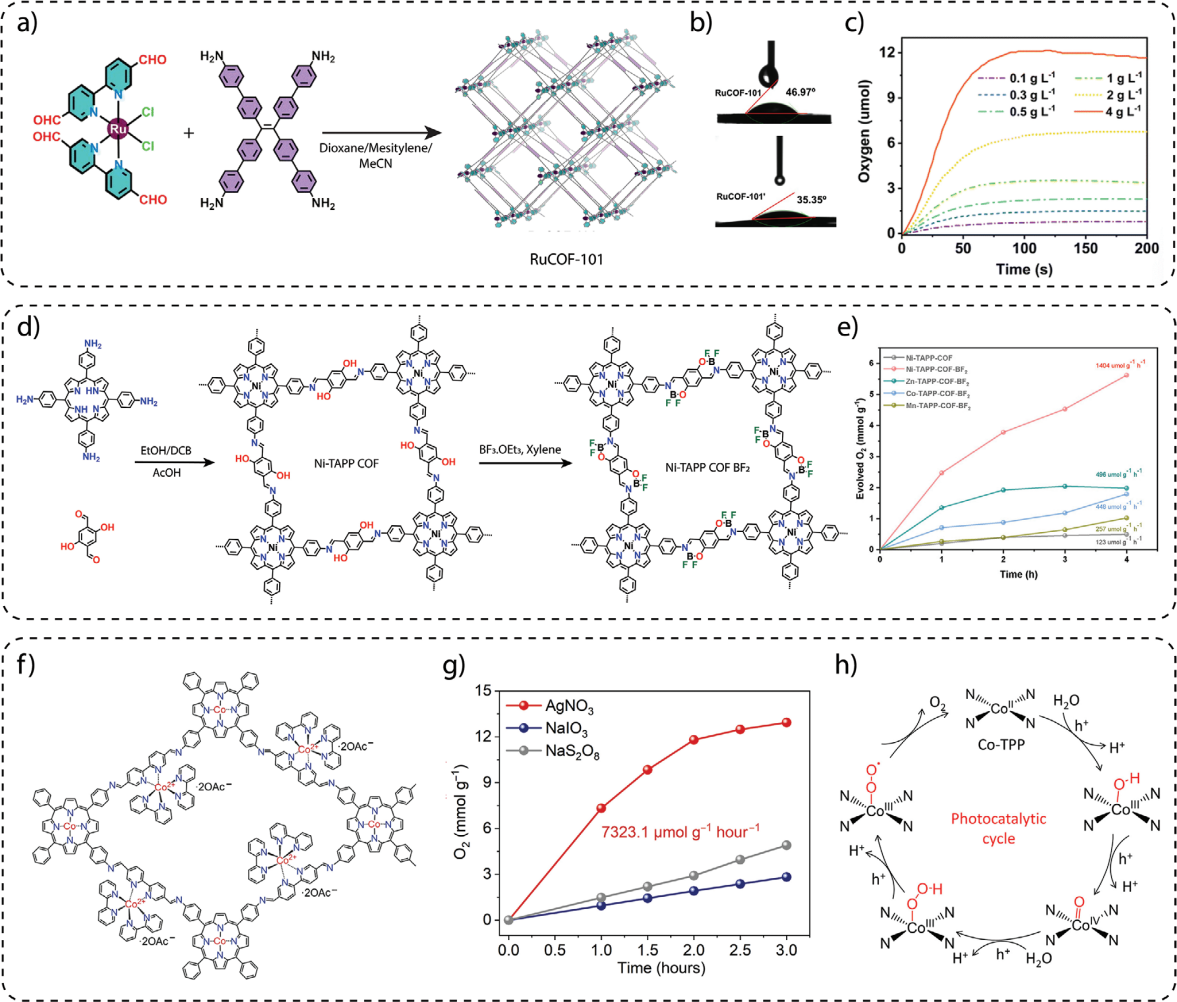
a) Synthesis of three‐dimensional RuCOF‐101. b) Contact angle measurement after ligand exchange indicating an increase in the hydrophilicity of RuCOF‐101′. c) Oxygen evolution performance of RuCOF‐101′ over time. Reproduce with permission.^[^ ^96^ ^]^ Copyright 2023. Royal Society of Chemistry. d) Synthesis of Ni‐TAPP COF and its post‐synthetic modified to Ni‐TAPP COF BF_2_. e) Oxygen evolution performance of M‐TAPP‐COF‐BF_2_ (M = Ni, Zn, Co, and Mn). Reproduce with permission.^[^ ^97^ ^]^ Copyright 2024. Wiley‐VCH. f) Schematic representation of CoTPP‐CoBpy_3_ COF. g) Photocatalytic oxygen evolution performance of CoTPP‐CoBpy_3_ COF in the presence of different sacrificial agents. h) Mechanistic pathway for photocatalytic oxygen evolution from CoTPP‐CoBpy_3_ COF. Reproduce with permission.^[^ ^98^ ^]^ Copyright 2024. Science.

Exploiting this advantage, Gu and co‐workers used a molecular ruthenium complex as a water oxidation catalyst (WOC) to construct a series of three‐dimensional metal covalent organic frameworks (3D MCOFs) (**Figure** **7**a). In a typical solvothermal synthesis, an aldehyde‐functionalized Ru(ii) complex [Ru(bpy‐CHO)_2_Cl_2_] was reacted with 4,4′,4″,4‴‐(ethene‐1,1,2,2‐tetrayl)tetraaniline (ETTA) and 4′,4‴,4″‴,4′‴‴‐(ethene‐1,1,2,2‐tetrayl)tetrakis(([1,1′‐biphenyl]‐4‐amine)) (ETTBA), respectively, to form RuCOF‐100 and RuCOF‐101.^[^ ^96^ ^]^ However, initial photocatalytic water oxidation measurements showed negligible activity for RuCOF‐100 and RuCOF‐101 due to the presence of coordinated chloride (Cl¯) ligands hindering water accessibility to the ruthenium (Ru) center. To overcome this challenge, a ligand exchange strategy using Ag⁺/H₂O was employed to remove the Ru‐coordinated Cl¯ ligands, which resulted in the formation of RuCOF‐100′ and RuCOF‐101′. Replacing the Cl¯ ligands increased the hydrophilicity of the COFs, improving their dispersibility in water, a crucial parameter for efficient water oxidation catalysis (Figure 7b). Interestingly, photocatalytic measurements in the presence of Ce(IV) oxidant showed a significant increase in oxygen evolution activity for RuCOF‐100′ and RuCOF‐101′ up to 640 µmol g^−1^ h^−1^ and 786 µmol g^−1^ h^−1^, respectively, (Figure 7c) due to the presence of periodically arranged Ru sites in the crystalline, porous and hydrophilic COF matrix.

Wang and co‐workers demonstrated photocatalytic water oxidation using metalloporphyrin‐based donor‐acceptor COFs with a boranil moiety (Figure 7d). The synthesis of Ni‐TAPP‐COF was achieved through the solvothermal reaction of 5, 10, 15, 20‐tetrakis (4‐aminophenyl) Ni[II] (Ni‐TAPP) and 2,5‐dihydroxy terephthalaldehyde (2,5‐DhA). To extend the π‐conjugation and photocatalytic properties of pristine Ni‐TAPP‐COF, a post‐synthetic modification strategy was applied. The synthesis of boranil‐functionalized Ni‐TAPP‐COF‐BF_2_ was achieved via a reaction of Ni‐TAPP‐COF with an excess of BF_2_.Et_2_O in dry *o*‐xylene at 130 °C for 12 h.^[^ ^97^ ^]^ The photocatalytic oxygen evolution studies performed under visible light irradiation (300 W Xe AM 1.5G) in a 100 mL aqueous solution containing 5 mg of the photocatalyst and 50 mM AgNO_3_ as the sacrificial electron acceptor showed an oxygen evolution rate of 1404 µmol g^−1^ h^−1^, which was about 11‐fold higher than the unmodified Ni‐TAPP‐COF (123 µmol g^−1^ h^−1^) (Figure 7e). The catalytic efficiency of Ni‐TAPP‐CO‐BF_2_ is attributed to the synergistic effect of intramolecular Ni‐porphyrin (donor) and BF_2_ (acceptor) functionalization, in addition to the enhanced light‐harvesting property, degree of π‐conjugation and tailored band structure.

To further improve the photocatalytic water oxidation performance, Wang and co‐workers successfully synthesized an ionic COF (CoTPP‐CoBpy_3_) through a one‐pot condensation reaction of 5,10,15,20‐Tetrakis(4‐aminophenyl)‐21H,23H‐porphine, 2,2′‐bipyridyl‐5,5′‐dialdehyde, 2,2′‐bipyridine and Co(OAc)_2_·4H_2_O in *o*‐dichlorobenzene (DCB)/*n*‐BuOH (2 ml/2 ml) and 6 M acetic acid at 120 °C for 3 days (Figure 7f).^[^ ^98^ ^]^ The CoTPP‐CoBpy_3_ COF exhibited excellent dispersibility in various solvents, including water, ethanol, and methanol, superhydrophilicity with a contact angle of ≈13.8° and spreading within 1 s. When photocatalytic oxygen evolution analyses were performed using AgNO_3_ as a sacrificial agent, CoTPP‐CoBpy_3_ showed superior photocatalytic oxygen evolution with a rate of 7300 µmol g^−1^ h^−1^, which was 24.3 times higher than that of the CoTPP‐Bpy under simulated sunlight (Figure 7g). This significant performance of CoTPP‐CoBpy_3_ was attributed to the dispersibility, suitable band structure, and synergistic electron‐intermediate cascade. For the mechanism of photocatalytic oxygen evolution, as verified by in situ attenuated total reflectance infrared spectrum (ATR‐ IR) and density functional theory (DFT) calculations, it was found that water molecules get adsorbed onto the Co‐centres of Co‐TPP COF, which are then deprotonated to form hydroxyl groups (OH^¯^). These OH^¯^ groups subsequently dissociate to form oxygen intermediates (*O). The formation of Co‐OOH species follows as the *O intermediates couple with additional water molecules. Under the influence of photogenerated holes, Co‐OOH undergoes dehydrogenation, producing an end‐on superoxide radical (Co–O₂˙) bound to the Co metal center. Finally, this Co–O₂˙ species dissociates, releasing oxygen molecules (O₂) and completing the water oxidation cycle (Figure 7h). The study has demonstrated a pathway to achieve an efficient photocatalytic performance for water oxidation using the COF photocatalyst.

#### 4.1.3 Photocatalytic Overall Water Splitting for H_2_ and O_2_ Generation

**Figure 8:**
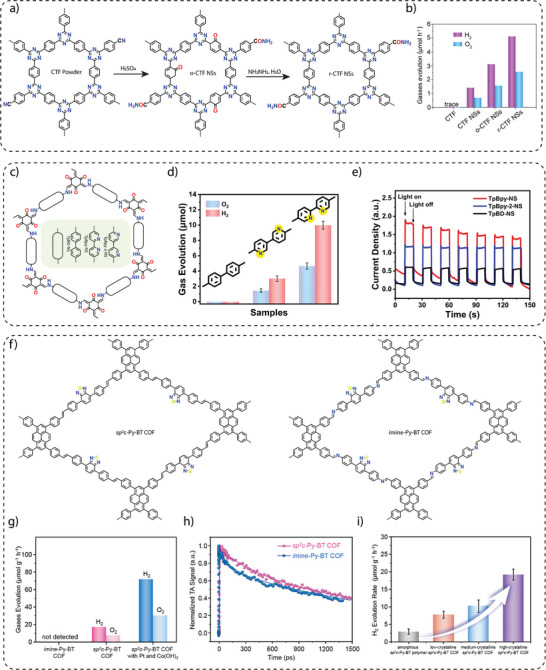
a) A redox strategy for the synthesis of o‐CTF NSs and r‐CTF NSs for water splitting. b) Hydrogen and oxygen evolution performance of CTF, CTF NSs, o‐CTF NSs, and r‐CTF NSs. Reproduce with permission.^[^ ^99^ ^]^ Copyright 2021. Wiley‐VCH. c) Synthesis of *β*‐ketoenamine‐based COFs varying the position of N sites. d) Comparison of OWS performance with varying the position of N sites in COFs. e) Photocurrent measurement indicating higher photo‐response of TpBpy‐NS compared to other analogs. Reproduce with permission.^[^ ^100^ ^]^ Copyright 2023. Springer Nature. f) Schematic representation of sp^2^c‐Py‐BT COF and imine‐Py‐BT COF. g) Comparison of overall water splitting performance for sp^2^c‐Py‐BT COF and imine‐Py‐BT COF. h) Time‐correlated single photon counting for sp^2^c‐Py‐BT COF and imine‐Py‐BT COF. i) H_2_ evolution performance with respect to the crystallinity of sp^2^c‐Py‐BT COF. Reproduce with permission.^[^ ^77^ ^]^ Copyright 2024. Wiley‐VCH.

Photocatalytic overall water splitting (OWS) for the simultaneous production of hydrogen and oxygen is an exciting process as it offers a clean and sustainable method of producing hydrogen and oxygen using sunlight, with the potential for large‐scale renewable energy production. However, it faces severe challenges, such as low efficiency due to poor charge separation, slow reaction kinetics, and the need for stable, cost‐effective photocatalysts that can operate under visible light and practical conditions. To achieve photocatalytic OWS, Xu and co‐workers fabricated ultrathin crystalline amide‐functionalized CTF nanosheets (CTF NSs) using a redox strategy that involved treatment of the layered CTF powder prepared by reacting terephthalonitrile by concentrated sulfuric acid followed by hydrazine hydrate (**Figure** **8**a).^[^ ^99^ ^]^ The photocatalytic hydrogen evolution rates were found to be 30.4, 155.5, 310.5, and 512.3 µmol g^−1^ h^−1^ for the pristine CTF, CTF NSs, oxidized o‐CTF NSs, and reduced r‐CTF NSs, respectively (Figure 8b). The superior photocatalytic performance of the exfoliated CTFs was attributed to the increased valence band (VB), narrow band gap and improved light absorption due to exfoliation. Notably, the reduced CTF NSs (r‐CTF NSs) NSs also showed overall photocatalytic performances with stoichiometric H_2_ (102.6 µmol h^−1^ g^−1^) and O_2_ (50.6 µmol h^−1^ g^−1^) evolution rates under visible light irradiation.

Lan and co‐workers synthesized a series of *β*‐ketamine COFs by varying the N‐sites in the bipyridyl units. Typically, the synthesis of ultra‐small Pt nanoparticle‐immobilized COFs, Pt(X%)@TpBpy, Pt(X%)@TpBpy‐2 and Pt(X%)@TpBD COFs was achieved by a solvothermal reaction of 1,3,5‐triformylphloroglucinol (Tp) with 2, 2′‐bipyridine‐5,5′‐diamine (Bpy), 3,3′‐bipyridine‐6,6′‐diamine (Bpy‐2) and benzidine (BD), respectively, in the presence of o‐dichlorobenzene, N,N‐dimethylacetamide (DMAc), Pt‐DMAc dispersion and aqueous acetic acid.^[^ ^100^ ^]^ Furthermore, the synthesis of COFs‐NS or Pt@COFs‐NS was achieved by ultrasonication followed by centrifugation to collect the nanosheets (Figure 8c). The total water splitting experiment, performed at 10 °C using deoxygenated water and a 300 W Xe lamp with a cut‐off filter (>420 nm), showed that both bipyridine‐based Pt@TpBpy‐NS and Pt@TpBpy‐2‐NS COFs simultaneously generate H_2_ and O_2_, whereas non‐bipyridine‐based Pt@TpBD‐NS showed negligible activity (Figure 8d). Pt@TpBpy‐NS demonstrated the optimal H_2_ and O_2_ generation of 9.9 and 4.8 µmol, respectively, in 5 hours, while Pt@TpBpy‐2‐NS achieved only 3.1 and 1.4 µmol of H_2_ and O_2_, respectively. These findings clearly showed the significant impact of the position of the N‐sites in the COF backbone, which facilitated electron transfer efficiencies and reduced the reaction potential barriers (Figure 8e).

Recent studies highlight that the covalent linkage between photoactive units is a key factor in determining the photocatalytic activity of COFs. As the imine linkages (─C═N─) exhibit high polarization and lesser π‐delocalization, fully conjugated sp^2^ carbon‐linked (─C═C─) COFs have been explored for their efficient π‐delocalization throughout the framework, enhancing carrier mobility and facilitating charge transfer. Xu and co‐workers explored the role of linkages in overall water splitting by constructing two types of π‐conjugated COFs: cyanovinylene‐linked sp^2^c‐Py‐BT COF and imine‐bridged imine‐Py‐BT COF synthesized from electron‐donating pyrene (Py) and electron‐accepting benzothiadiazole (BT) units via Knoevenagel condensation and Schiff‐base reactions, respectively (Figure 8f).^[^ ^77^ ^]^ Under visible light irradiation (λ > 420 nm, 300 W Xe lamp), sp^2^c‐Py‐BT COF achieved an average H_2_ and O_2_ production rate of 17.2 and 8.1 µmol h^−1^ g^−1^, respectively, notably without metal cocatalysts. In the presence of Pt and Co(OH)_2_ cocatalyst, the photocatalytic performance of the sp^2^c‐Py‐BT COF improved, and H_2_ and O_2_ evolution rates can increase to 71.3 and 30.8 µmol h^−1^ g^−1^, respectively (Figure 8g). In contrast, no gaseous products were detected with imine‐Py‐BT COF, confirming its inactivity for overall water splitting. A lower exciton binding energy for the sp^2^c‐Py‐BT COF than for the imine‐Py‐BT COF and subsequent promotion of charge separation was found to be the main reason for the enhanced activity for sp^2^‐carbon‐linked COFs (Figure 8h). Furthermore, optimizing the crystallinity of the sp^2^c‐Py‐BT COF showed that larger crystalline domains improve the photocatalytic performance (Figure 8i), demonstrating the crucial role of crystallinity in photocatalysis.

In summary, reports on total water splitting using COFs are still scarce, although the study of the half‐reactions of the water splitting reaction using sacrificial electron donors or acceptors can only be a first step to investigating possible and promising materials for OWS, the only economically viable method for photocatalytic hydrogen production. Even though these studies have made remarkable progress in identifying crucial parameters to enhance the photocatalytic activity of COFs, it is still not possible to predict if a COF is also suitable for overall water splitting. Indeed, the few reported COFs for OWS do not possess significant features regarding structure or optical properties, which would differentiate them from COFs, which are highly active in HER but function exclusively when using a SED. The fact that the reports described here for COFs for OWS are using nanosheets could mean that, in addition to all the factors mentioned, more attention should be paid to the shape and morphology of the COFs. Finding the “secret ingredient” that turns a COF from a good HER to an efficient OWS photocatalyst is certainly one of the most exciting research questions in our field.

### 4.2 Photocatalytic Carbon Dioxide (CO_2_) Reduction

Solar‐driven CO_2_ conversion is a promising approach to combat global warming and the energy crisis. This process mimics natural photosynthesis to produce valuable fuels and chemicals from CO_2_ such as methane, methanol, ethanol, acetic acid, carbon monoxide, and formic acid. Halmann and Inoue first demonstrated the photocatalytic conversion of CO_2_ to hydrocarbons using inorganic semiconductors.^[^ ^101^ ^]^ To date, the most reported photocatalysts for CO_2_ reduction are metal‐containing semiconductors such as metal oxides, metal sulfides, or zeolites. COFs are considered a potential catalyst for CO_2_ reduction as they combine the structural tunability and high surface area of organic frameworks with the stability and reusability advantages over inorganic semiconductors and homogeneous complexes, enabling more efficient and selective catalytic performance.^[^ ^102^, ^103^ ^]^ The applications of COFs for CO_2_ reduction are summarized in the following sections:

#### 4.2.1 Metal‐Free COFs for CO_2_ Reduction

**Figure 9:**
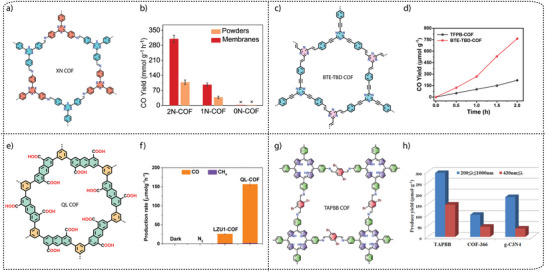
Schematic representation of the covalent organic frameworks (COFs) and their application in carbon dioxide reduction. a) Structure of XN COF. b) Photocatalytic applications of XN COF. Reproduced with permission.^[^ ^104^ ^]^ Copyright 2023. American Chemical Society. c) Structure of BTE‐TBD COF. d) Photocatalytic applications of BTE‐TBD COF. Reproduced with permission.^[^ ^105^ ^]^ Copyright 2023. Wiley‐VCH. e) Structure of QL COF. f) Photocatalytic applications of QL COF. Reproduced with permission.^[^ ^106^ ^]^ Copyright 2021. Royal Society of Chemistry. g) Structure of TAPBB COF. h) Photocatalytic applications of TAPBB COF. Reproduced with permission.^[^ ^107^ ^]^ Copyright 2020. Wiley‐VCH.

Materials such as graphitic carbon nitride (*g*‐C_3_N_4_), CMPs, covalent organic polymers (COPs), and linear conjugated polymers have demonstrated activity in photocatalytic CO_2_ reduction. In addition, metal‐free COFs constituted from light elements offer a sustainable and versatile alternative, providing tailor‐made crystallinity, porosity, and the possibilities of the integration of various functional moieties — ideal for CO_2_ reduction. The various strategies for improving the CO_2_ reduction performances of COFs can be summarized as follows:

- **Electronic property modulation**: It has been demonstrated that the incorporation of donor‐acceptor (D‐A) moieties into COFs enhances photocatalytic performance by improving light absorption, charge separation, and transport. In addition, nitrogen‐rich moieties such as triazine and heptazine interact with CO_2_ molecules to further promote the CO_2_ reduction reaction. Given the advantages of donor‐acceptor materials, several advances have been made by incorporating different moieties into the COF backbone. For example, Wang and co‐workers synthesized a series of COFs, namely 0N‐COF, 1N‐COF, and 2N‐COF, by varying the number of nitrogen atoms in a central phenyl unit (X = 0 to 2) (**Figure** **9**a).^[^ ^104^ ^]^ Membranes of 0N‐COF, 1N‐COF, and 2N‐COF were prepared by a one‐step synthesis at the ionic liquid‐H_2_O interface of different amines. The crystalline and porous COF membranes exhibited light absorption in the visible region. CO_2_ photoreduction experiments performed in water under gas‐solid conditions under visible light irradiation (λ ≥ 420 nm) without the addition of any metal, photosensitizer, or sacrificial agent showed that 2N‐COF membranes exhibited a maximum CO_2_ to CO conversion rate of 310 µmol h^−1^ g^−1^. The conversion efficiency of 2N‐COF was found to be 3.2 times higher than that of 2N‐COF powders (98 µmol h^−1^ g^−1^) (Figure 9b). The apparent quantum efficiency (AQE) for 2N‐COF was found to be 0.36% in the visible light region (420 nm) and recyclability studies showed long‐term catalytic stability of the 2N‐COF membrane, with the CO production yield decreasing only slightly to 287 µmol g^−1^ h^−1^ after 16 cyclic runs.
- **Band gap and band edge position engineering**: Incorporating suitable molecular building blocks in the COF backbone is a key strategy for enhancing the photocatalytic performance for CO_2_ reduction. The choice of building blocks that promotes planarity, π–π conjugation, and stacking of the layers can lead to improved charge separation, reduced charge recombination, and increased photo absorption. For instance, Yang and co‐workers employed ethyne moieties in the conjugation of a triazine ring and formed two new COFs, TFPB‐COF and BTE‐TBD‐COF using base‐promoted aldol condensation between triazine derivative 2,4,6‐trimethyl‐s‐triazine (TMT) and aromatic aldehydes monomers 2,4,6‐tris(4‐formylphenyl)‐1,3,5‐benzene (TFPB) or 4,4″,4″’‐(benzene‐1,3,5‐ triyltris(ethyne‐2,1‐diyl))tribenzaldehyde (BTE‐TBD), respectively (Figure 9c).^[^ ^105^ ^]^ The valence band position and conduction band position of BTE‐TBD COF were found to be located at 1.92 eV and −0.77 eV vs NHE, which are larger than the corresponding band positions of TFPB‐COF. The conduction band position favored the reduction of CO_2_ to CO, while the valence band position could easily oxidize water, thus BTE‐TBD‐COF achieved a CO production rate of 382.03 µmol h^−1^ g^−1^, which is three times higher than TFPB‐COF (109.8 µmol h^−1^ g^−1^) (Figure 9d).
- **Introduction of hydrophilicity in the COF backbone**: The integration of hydrophilic groups into a COF system can significantly enhance the photocatalytic CO₂ reduction activity, particularly when water is used as the electron donor. Considering this, Jin and co‐workers synthesized two distinct COFs, LZU1‐COF and QL‐COF, which differ considerably in terms of hydrophilicity. LZU1‐COF was synthesized through a Schiff‐base condensation reaction between 1,3,5‐triformylbenzene and 1,4‐diaminobenzene. On the contrary, QL‐COF was constructed using a one‐pot Doebner reaction involving 1,3,5‐triformylbenzene, 1,4‐diaminobenzene and pyruvic acid, which resulted in the incorporation of 4‐carboxylquinoline linkages — forming a network of hydrophilic carboxylic acid groups on the pore surface (Figure 9e).^[^ ^106^ ^]^ It was observed that QL‐COF achieved a CO production rate of 156 µmol g⁻¹ h⁻¹ with a selectivity of 99.3%, which is six times higher than the CO production rate of LZU1‐COF (25 µmol g⁻¹ h⁻¹) (Figure 9f). Additionally, density functional theory (DFT) calculations suggested that QL‐COF has more negative binding energies for water (−12.5 kcal) and CO₂ (−5.8 kcal) compared to LZU1‐COF (−7.7 kcal for H₂O and −4.7 kcal for CO₂). This indicates a stronger adsorption affinity for both water and CO₂ molecules on the surface of QL‐COF, favoring its higher catalytic efficiency. Furthermore, the 4‐carboxylquinoline linkage in QL‐COF improved its light‐harvesting ability and enhanced charge separation and transfer, which further boosted its performance in CO₂ photoreduction.
- **Functionalization of COFs**: In addition to regulating pore size, the introduction of electron‐rich building blocks into COFs is also beneficial for the catalytic process. In particular, porphyrin with an electron‐rich conjugated structure can transfer electrons to the catalytic moiety through conjugation, forming an elongated conjugated system, making it suitable for electrocatalysis and photocatalysis. The electronic properties of organic ligands can also be altered by the introduction of halogen groups such as bromine. To take advantage of these properties, Su and co‐workers synthesized a porphyrin‐based TAPBB‐COF through a solvothermal reaction of 5,10,15,20‐tetrakis(4‐aminophenyl)porphyrin] and bromine‐functionalized 2,5‐dibromo‐1,4‐benzenedialdehyde (Figure 9g).^[^ ^107^ ^]^ For comparison, a bromine‐free non‐functionalized COF‐366 was also prepared by a similar reaction of 5,10,15,20‐tetrakis(4‐aminophenyl)porphyrin] with terephthaldehyde. Under simulated sunlight (200≤λ≤1000 nm), TAPBB‐COF was found to exhibit photocatalytic CO_2_ reduction to CO at a rate of 24.6 µmol g^−1^ h^−1^ in the absence of metals and sacrificial agents. The incorporation of bromine groups into the COF backbone enhanced CO production by a factor of 3 compared to COF‐366 (8.5 µmol h^−1^ g^−1^) due to the change in energy levels, thereby improving charge separation and CO_2_ activation (Figure 9h). It is anticipated that nitrogen atoms in the porphyrin ring and the Schiff‐base contribute to CO_2_ activation, while bromine enhances water adsorption. TAPBB‐COF also showed good stability over multiple cycles, demonstrating the promise of functional group design for efficient photocatalytic CO_2_ reduction with water.

Fan and co‐workers demonstrated the effect of different functional groups on the photocatalytic activity of two‐dimensional *β*‐ketoenamine COFs (TpBD‐X).^[^ ^108^ ^]^ To analyze the effect of different substituents such as –H_2_, –(CH_3_)_2_, –(OCH_3_)_2_, and –(NO_2_)_2_ present in the COF framework on photocatalytic CO_2_ reduction, a series of COFs were prepared solvothermally by reacting 1,3,5‐triformylphloroglucinol with benzidine linkers functionalized with different functional groups. Photocatalytic experiments showed that electron‐donating groups have a positive effect on CO_2_ reduction by improving photogenerated charge transfer and light harvesting, resulting in higher photocatalytic formic acid (HCOOH) formation for TpBD‐(OCH_3_)_2_ (108.3 µmol g^−1^ h^−1^) and TpBD‐(CH_3_)_2_) (86.3 µmol g^−1^ h^−1^) compared to TpBD‐H_2_. TpBD‐(NO_2_)_2_ also showed a lower rate of 22.2 µmol g^−1^ h^−1^. The superior performance of TpBD‐(OCH_3_)_2_ and TpBD‐(CH_3_)_2_ is attributed to reduced charge transfer resistance and improved charge separation efficiency, as the methoxy and methyl groups have electron donating properties that facilitate electron transfer to CO_2_ for HCOOH formation.

#### 4.2.2 Metallated COFs for CO_2_ Reduction

##### Reduction of CO_2_ to CO

**Figure 10:**
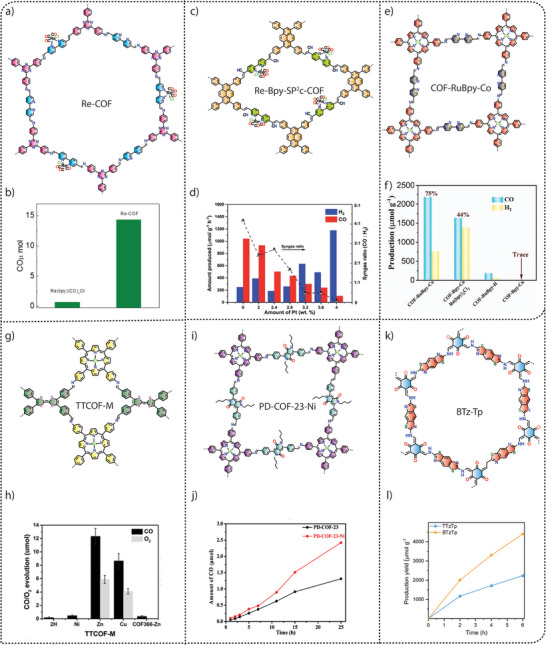
a) Schematic representation of the metal‐incorporated covalent organic frameworks (COFs) and their application in carbon dioxide reduction. a) Structure of Re‐COF. b) Photocatalytic applications of Re‐COF for CO_2_ reduction. Reproduced with permission.^[^ ^109^ ^]^ Copyright 2018. American Chemical Society. c) Structure of Re‐Bpy‐sp^2^‐c COF. d) Photocatalytic applications of Re‐Bpy‐sp^2^‐c COF for CO_2_ reduction. Reproduced with permission.^[^ ^110^ ^]^ Copyright 2018. Royal Society of Chemistry. e) Structure of RuBpy‐Co COF. f) Photocatalytic applications of RuBpy‐Co COF for CO_2_ reduction. Reproduced with permission.^[^ ^111^ ^]^ Copyright 2023. Elsevier. g) Structure of TTCOF‐M. h) Photocatalytic applications of TTCOF‐M for CO_2_ reduction. Reproduced with permission.^[^ ^112^ ^]^ Copyright 2019. Wiley‐VCH. i) Structure of PD‐COF‐23‐Ni. j) Photocatalytic applications of PD‐COF‐23‐Ni for CO_2_ reduction. Reproduced with permission.^[^ ^113^ ^]^ Copyright 2020. Royal Society of Chemistry. k) structure of BTz‐Tp COF. l) Photocatalytic applications of BTz‐Tp COF for CO_2_ reduction. Reproduced with permission.^[^ ^114^ ^]^ Copyright 2021. American Chemical Society.

Reducing CO_2_ to CO is advantageous because CO is a valuable feedstock for producing fuels and chemicals through processes such as Fischer‐Tropsch synthesis. It is also a more energy‐efficient route than further reduction to hydrocarbons, requiring fewer electrons and protons. In addition, the conversion of CO_2_ to CO helps to reduce greenhouse gas emissions, contributing to environmental sustainability and enabling the storage of renewable energy in chemical bonds. The CO_2_ reduction to CO achieved by the COF photocatalyst is summarized in the following sections:

i) Metal‐bipyridine COFs for CO_2_ reduction: Taking advantage of COFs as heterogeneous systems, in 2018, Huang and co‐workers first investigated CO_2_ photoreduction using a two‐dimensional COF incorporated with the rhenium (Re) complex, demonstrating the effective conversion of CO_2_ to CO under visible light irradiation.^[^ ^109^ ^]^ In this work, a rhenium‐COF hybrid photocatalytic system was constructed by post‐synthetic modification of BPDA‐TTA‐COF, which was synthesized via a Schiff‐base condensation of 2,2′‐bipyridyl‐5,5′‐dialdehyde (BPDA) and 4,4′,4′'‐(1,3,5‐triazine‐2,4,6‐triyl)trianiline (TTA) under solvothermal conditions. The Re‐moiety was introduced via a reaction between the bipyridine ligand from COF and Re(CO)_5_Cl to form Re‐COF (**Figure** **10**a). The resulting Re‐COF exhibited significant photocatalytic activity when TEOA was used as the sacrificial donor and a Xe lamp (cut‐off wavelength = 420 nm) as the light source, producing carbon monoxide (CO) at a rate of 15 µmol g^−1^ with 98% selectivity over 20 h, outperforming the original complex by 22‐fold (Figure 10b). Mechanical studies revealed that the efficient intramolecular charge transfer from the excited COF to the Re moiety reduces charge recombination and increases the efficiency of CO_2_ photoreduction.

Furthermore, the incorporation of Re‐metal complexes into fully conjugated sp^2^‐carbon‐conjugated (─C═C─) COFs was expected to significantly enhance catalytic activity through improved π‐delocalization and charge carrier mobility. In this regard, Cooper and his group synthesized Re‐centered Re‐Bpy‐sp^2^c‐COFs by embedding pyrene and bipyridine units in the framework and introducing Re(CO)_5_ complexes (Figure 10c).^[^ ^110^ ^]^ A two‐dimensional Bpy‐sp^2^‐c‐COF was synthesized via the Knoevenagel condensation of 1,3,6,8‐tetrakis(4‐formylphenyl)pyrene (TFPPy) and 5,5′‐bis(cyanoethyl)‐2,2′‐bipyridine. The bipyridine sites in Bpy‐sp^2^c‐COF were used to ligate [Re(CO)_5_Cl] to yield Re‐Bpy‐sp^2^c‐COF. Although the re‐doping reduced the BET surface area, Re‐Bpy‐sp^2^c‐COF exhibited enhanced CO_2_ adsorption compared to pristine Bpy‐sp^2^c‐COF. The photocatalytic CO_2_ reduction analyses under 1 atmosphere of CO_2_ in a mixture of acetonitrile and triethanolamine and under visible light illumination (λ > 420 nm, 300 W Xe light source) showed that Re‐Bpy‐sp^2^c‐COF exhibited a remarkable CO generation rate of 1040 µmol g^−1^ h^−1^ with 81% selectivity over HER, with a TON of 18.7 (Figure 10d). The active Re‐sites and donor‐acceptor pair of pyrene and bipyridine facilitated efficient charge transfer, resulting in superior CO_2_ photocatalysis performance. These results indicate that the attachment of metal complexes in a planar and fully conjugated framework significantly improves the efficiency of CO_2_ reduction by stabilizing intermediates and facilitating electron transfer. This enhanced interaction allows for more effective activation and conversion of CO_2_ into valuable products.

ii) **Metal‐porphyrin COFs for CO_2_ reduction**: Porphyrins are advantageous for CO_2_ reduction due to their strong light absorption, tunable electronic properties, and ability to stabilize CO_2_ intermediates. Their conjugated structure and coordination with metals allow effective electron transfer, increasing the efficiency and selectivity of the catalytic process. Considering these advantages, Cao and co‐workers developed a porphyrin‐based photocatalyst, COF‐RuBpy‐Co, by reacting Cobalt porphyrin and 2,2′‐bipyridyl‐5,5′‐dialdehyde (Figure 10e), featuring [Ru(bpy)_3_]^2+^ as photosensitizer units and metalloporphyrin as a catalytic site for CO_2_ photoreduction.^[^ ^111^ ^]^ The COF‐RuBpy‐Co showed significantly enhanced CO_2_ capture as compared to pristine materials. COF‐RuBpy‐Co showed a CO production rate of 547 µmol g^−1^ h^−1^, 1.4 times greater than a physical mixture of the same COF and photosensitizer (Figure 10f). DFT studies revealed high molecular orbital overlap enhancing charge migration, while in situ XPS confirmed efficient electron transfer from [Ru(bpy)_3_]^2+^ to cobalt sites, underscoring the importance of covalently linking photosensitizer and porphyrin moieties to boost photocatalytic activity.

Donor‐acceptor materials are advantageous for CO_2_ reduction because these materials facilitate efficient charge separation and transfer, thereby reducing recombination rates. This improves the generation of active sites for CO_2_ conversion, leading to higher catalytic efficiency and selectivity in the reduction process. In this context, the synthesis of a series of two‐dimensional rigid porphyrin‐tetrathiafulvalene COFs (TTCOF‐M, M = 2H, Zn, Ni, Cu) for CO_2_ reduction (Figure 10g) was demonstrated via a solvothermal reaction between metalloporphyrins (MTAPP, M = 2H, Zn, Ni, Cu) and 2,3,6,7‐tetra(4‐formylphenyl)‐tetrathiafulvalene (TTF).^[^ ^112^ ^]^ CO_2_ adsorption isotherms for TTCOF‐M (M = 2H, Zn, Ni, Cu) showed adsorption capacities of 28, 52, 42 and 38 cm^3^ µmol g^−1^ h^−1^, respectively, reflecting different CO_2_ affinities based on the metal ions in the COFs. The photocatalytic CO_2_ reduction analyses, carried out using CO_2_‐bubbled deionized water and under irradiation with a 300 W xenon arc lamp (intensity: 400 mW cm^−2^) with a cut filter (range: 420 to 800 nm), showed that TTCOF‐Zn is the most efficient for selective CO_2_ photoreduction, producing 12.33 µmol of CO compared to TTCOF‐Cu (8.62 µmol) and TTCOF‐Ni (0.462 µmol) (Figure 10h). The combination of electron‐deficient metalloporphyrin and electron‐rich tetrathiafulvalene efficiently transfers and separates visible light‐driven electrons from TTF to TAPP, allowing reduction and oxidation reactions. Photophysical analyses showed that the conduction band maximum of TTCOF‐Zn is more negative than the standard reduction potential of CO/CO_2_ and its valence band minimum is more positive than the oxidation potential of O_2_/H_2_O, suggesting its ability to integrate CO_2_ reduction with H_2_O oxidation reactions compared to another TTCOF‐M COFs. In similar attempts, Zhu and co‐workers reported two donor‐acceptor porphyrin‐based COFs, PD‐COF‐23 and PD‐COF‐23‐Ni, synthesized by an acid‐catalyzed Schiff‐base condensation reaction of 2,5‐dibutyl‐3,6‐bis(4‐formylphenyl)‐pyrrolo[3,4‐c]pyrrole‐1, 4‐dione (DPP‐CHO) with 5,10,15,20‐tetrakis(4‐aminophenyl)porphyrin (TAPP) or meso‐5,10,15,20‐tetra‐(4‐aminophenyl)porphyrinato Ni(II) (TAPP‐Ni), respectively (Figure 10i).^[^ ^113^ ^]^ The photocurrent response of PD‐COF‐23‐Ni was found to be higher than that of PD‐COF‐23, indicating efficient photogenerated hole‐electron pair separation and greater availability of electrons on the surface. The photocatalytic CO_2_ reduction analyses performed using PD‐COF‐23 or PD‐COF‐23‐Ni as the photocatalyst and triethanolamine (TEOA) as the sacrificial electron donor showed that PD‐COF‐23‐Ni exhibited a CO_2_ production rate of 40.0 µmol g^−1^ h^−1^ in 25 h, outperforming PD‐COF‐23 which produced 20.9 µmol g^−1^ h^−1^ CO in the same time (Figure 10j). The selectivity for CO production reached 99%, demonstrating the promise of porphyrin COFs for photocatalytic CO_2_ reduction.

iii) Immobilization of metal complexes in COFs for CO_2_ reduction: Since CO_2_ reduction involves three primary processes such as photoabsorption, carrier separation, and CO_2_ reduction. Photocatalysts for CO_2_ reduction require a sufficient number of optically active centers to generate photoinduced charges and efficient channels for the transfer of these carriers to a catalytically active center with high selectivity. In these regards, it has been shown that photocatalytic efficiency can be regulated by tuning the pore size and surface area – the inherent properties of COFs. To investigate the role of these properties, Beak and co‐workers prepared TTzTp and BTzTp COFs by a solvothermal reaction combining triformylglucinol (Tp) and tris‐benzothiazole triamine (TTz) or bis‐benzothiazole diamine (BTz) building blocks (Figure 10k).^[^ ^114^ ^]^ BTzTp COF showed a higher surface area (1920 m^2^ g^−1^) than TTzTp COF (985 m^2^ g^−1^), while UV‐vis diffuse reflectance spectroscopy analyses showed that TTzTp had a lower optical band gap (≈1.8 eV) than BTzTp (≈2.1 eV). The isosteric heat adsorption (Q_st_) of TTzTp, which is proportional to the CO_2_ affinity, was found to be higher than that of BTzTp due to the polarity effect arising from the more densely present heteroatoms in the benzothiazole core. The photocatalytic CO_2_ reduction experiments were carried out using Re(CO)_5_Cl as a co‐catalyst and triethanolamine as a sacrificial donor under an Xe lamp (cut‐off wavelength 400 nm). Despite a narrower light absorption range due to the conjugated structure of the building units, BTzTp showed a higher photocatalytic CO production rate (1002 µmol g^−1^ h^−1^) than TTzTp (586 µmol g^−1^ h^−1^) with a higher apparent quantum efficiency and selectivity (∼98%) (Figure 10l). This study demonstrated the influence of crystallinity and surface area, which are the driving forces for the higher photocurrent and lower charge transfer resistance, thereby enhancing the photocatalytic CO_2_ reduction of value‐added products.

**Figure 11:**
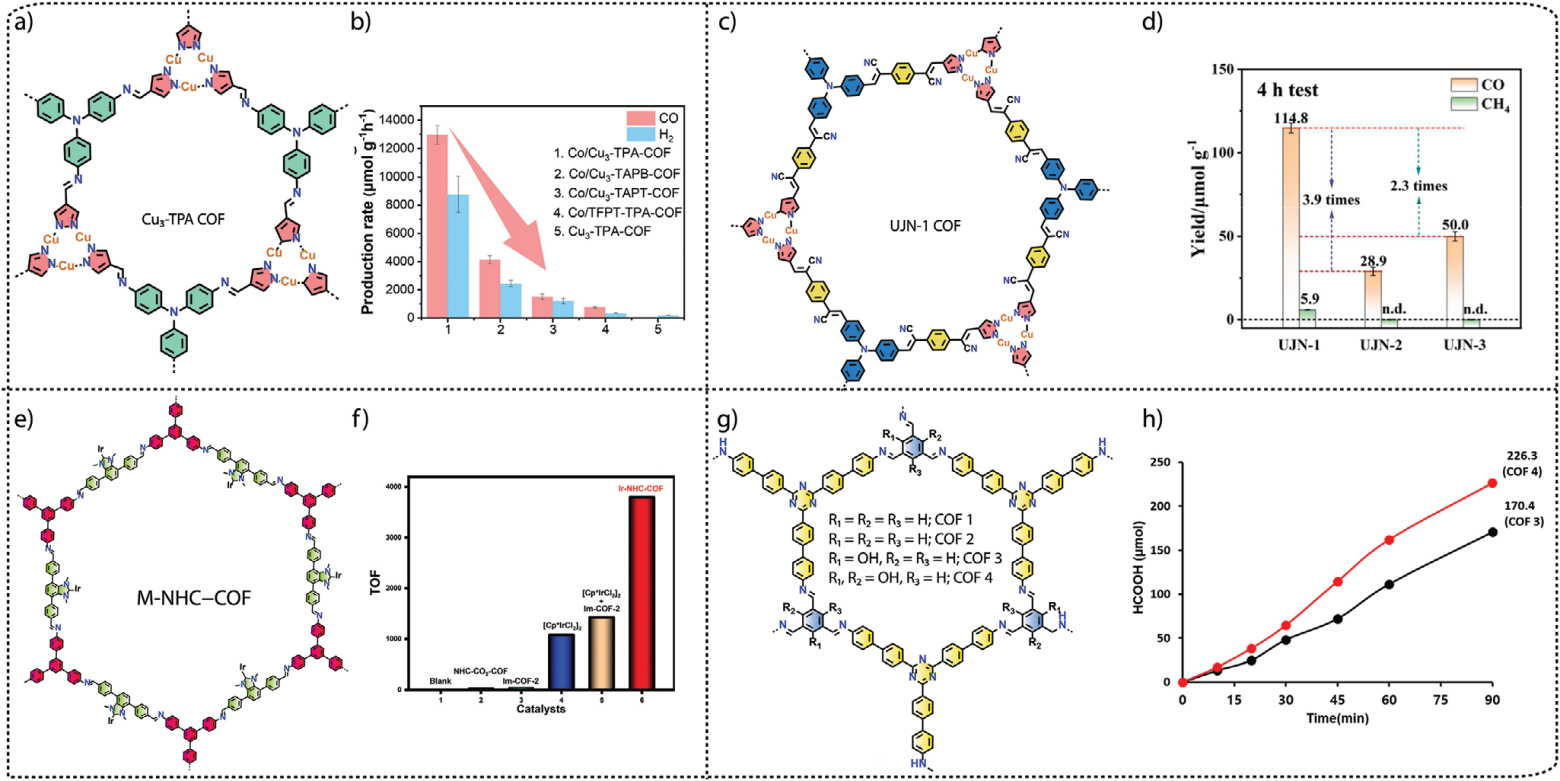
Schematic representation of the metal‐incorporated covalent organic frameworks and their application in carbon dioxide reduction. a) Structure of Cu_3_‐TPA COF. b) Photocatalytic applications of Cu_3_‐TPA COF for CO_2_ reduction to CO. Reproduced with permission.^[^ ^115^ ^]^ Copyright 2024. Wiley‐VCH. c) Structure of UJN‐1 COF. d) Photocatalytic applications of UJN‐1 COF for CO_2_ reduction to CO. Reproduced with permission.^[^ ^116^ ^]^ Copyright 2024. Wiley‐VCH. e) Structure of M‐NHC COF. f) Photocatalytic applications M‐NHC COF for CO_2_ reduction to formic acid. Reproduced with permission.^[^ ^118^ ^]^ Copyright 2024. American Chemical Society. g) Structure of COF (1–4). h) Photocatalytic applications of COF (1‐4) for CO_2_ reduction to formic acid. Reproduced with permission.^[^ ^61^ ^]^ Copyright 2021. American Chemical Society.

The catalytic performance of pristine COFs is often hampered by the absence of catalytic active sites and inefficient charge separation. To address these issues, Chen and co‐workers synthesized three isostructural COFs using a Donor1‐Acceptor‐Donor2 (D1‐A‐D2) motif through Schiff‐base condensation reactions. The reactions were performed using Cu_3_Py_3_ and amine linkers such as 2,4,6‐tris(4‐aminophenyl)‐1,3,5‐triazine (TAPT), 1,3,5‐tris(4‐aminophenyl)benzene (TAPB) or 4,4,4‐triaminotriphenylamine (TPA) (**Figure** **11**a).^[^ ^115^ ^]^ This strategy led to the development of COFs with distinct architectures and tunable local charge distributions, namely, Cu_3_‐TPA‐COF, Cu_3_‐TAPB‐COF, and Cu_3_‐TAPT‐COF. For comparison, a new COF structure incorporating single cobalt sites coordinated with imine nitrogen atoms was also synthesized. This configuration facilitated efficient charge transfer and stabilized charge separation in the excited state, resulting in directional electron flow from the two donor sites to the intercalated cobalt center. As a result, the catalytic center becomes highly active for CO_2_ activation and reduction. Experimental findings revealed that the Co/Cu_3_‐TPA‐COF demonstrated superior photocatalytic activity for CO_2_ reduction to syngas (13 028 µmol g⁻¹ h⁻¹), which was approximately 3.2, 8.4, and 17.8 times higher compared to Co/Cu_3_‐TAPB‐COF, Co/Cu_3_‐TAPT‐COF, and Co/TFPT‐TPA‐COF, respectively (Figure 11b). In addition, the incorporation of various bipyridine additives allowed precise control of the CO/H_2_ ratio, making the system adaptable to specific syngas production requirements.

In similar attempts, Yu and co‐workers developed a novel metal‐covalent organic framework (MCOF) named UJN‐1 to enhance the photocatalytic efficiency for CO_2_ reduction through a donor‐π‐acceptor (D‐π‐A) configuration – addressing the sluggish charge separation kinetics typically limiting COF performance. UJN‐1 was synthesized via Knoevenagel condensation involving tris(4‐formylphenyl)amine (TFPA), a copper cluster [Cu_3_L_3_], and 1,4‐phenyldiacetonitrile (PDAN). To investigate the effect of the vinylene linkage on charge separation, an imine‐linked MCOF, UJN‐2, was also prepared using TFPA, the Cu_3_L_3_ cluster, and 1,4‐phenylenediamine (PDA). In addition, to investigate the effect of metal on CO_2_ reduction, UJN‐3 MCOF was prepared, with a similar structure to UJN‐1, but the Cu_3_L_3_ cluster was replaced by the metal‐free precursor 1,3,5‐tris(4‐formylphenyl)benzene (TFPB) (Figure 11c).^[^ ^116^ ^]^ This design effectively connected electron‐rich triphenylamine units to electron‐deficient cyclic trinuclear copper units, facilitating better intramolecular charge separation and enhancing light‐harvesting capabilities. Under visible light, UJN‐1 demonstrated a CO formation rate of 114 µmol g⁻¹ and a selectivity of 95%, significantly outperforming other COF‐based photocatalysts (Figure 11d). The effective donor‐acceptor interactions of the framework stabilized the generated charges, prevented recombination, and improved the CO_2_ reduction efficiency. The inclusion of vinylene linkers is crucial to maintaining strong π‐conjugation, optimizing the electronic environment around the metal centers, and enhancing CO_2_ adsorption and activation. The results establish UJN‐1 as a benchmark for future COF designs aimed at high‐performance CO_2_ reduction, providing valuable insights into the development of functional MCOFs for broader applications in sustainable catalysis.

Compared to single‐atom catalysts with single catalytic sites, dual‐atom catalysts with bimetallic centers have the potential to combine atom‐specific properties and more complex functionalities between neighboring active sites. To achieve the synthesis of dual‐atom catalysts, the immobilization of metal complexes in the COF matrix has been demonstrated for efficient CO_2_ reduction to CO. The synthesis of dual‐atom catalysts with bimetallic centers (LaNi‐Phen/COF‐5) has been achieved by the reaction of LaCl_3_.6H_2_O, NiCl_2_.6H_2_O, and 1,10‐phenanthroline by adsorption and chelation with COF‐5 colloid, which was solvothermally synthesized by the reaction of 2,3,6,7,10,11‐hexahydroxytriphenylene and 1,4‐phenylenediboronic acid.^[^ ^117^ ^]^ The La‐Ni coordinated phenanthroline is incorporated into the COF‐5 colloid by electrostatically driven self‐assembly in which the La and Ni atoms are captured by B atoms from COF‐5 and chelated by the phenanthroline ligands. The crystalline and porous COF‐5 immobilized with bimetallic centers, LaNi‐Phen/COF‐5, exhibited a red shift in light absorption compared to Ni‐Phen/COF‐5, La‐Phen/COF‐5, and pristine COF‐5, indicating a more favorable energy level alignment. The photocatalytic CO_2_ reduction experiments were performed using 1,3‐dimethyl‐2‐phenyl‐2,3‐dihydro‐1H‐benzo[d]imidazole (BIH) as reductant and water‐acetonitrile mixture in the presence of ∼80 kPa of high purity CO_2_ irradiated with a 300 W xenon lamp (≈100 mW cm^−2^). These analyses showed that LaNi‐Phen/COF‐5 exhibited the highest catalytic activity of 608 µmol g^−1^ h^−1^ for CO_2_ reduction to CO and a selectivity of 98.2% (CO over H_2_), which was 15.2, 3.1 and 2.7 times higher, respectively, compared to COF‐5, La‐Phen/COF‐5 and Ni‐Phen/COF‐5. A physical mixture of La‐Phen/COF‐5 and Ni‐Phen/COF‐5 exhibited significantly lower catalytic activity for CO production (115.9 µmol g^−1^ h^−1^), indicating the role of Ni and La sites as predominant optical and catalytic centers, respectively, to promote light absorption and facilitate electron transfer.

##### Reduction of CO_2_ to Formic Acid

CO_2_ reduction to formic acid is beneficial because it is a valuable intermediate for chemical synthesis and can be used directly as a hydrogen storage material or fuel. In addition, formate has applications in various industries, including pharmaceuticals and agriculture, and its production from CO_2_ supports carbon capture and utilization strategies, contributing to environmental sustainability. *N*‐heterocyclic carbene (NHC) complexes are organometallic compounds in which a nitrogen‐containing heterocycle stabilizes a nucleophilic carbene carbon that binds strongly to metal centers. These complexes are widely used in catalysis, organometallic chemistry, and materials science due to their stability, tunable electronic properties, and versatility in facilitating various chemical reactions. NHC complexes are particularly effective in catalyzing the conversion of CO_2_ into fuels and chemicals, making them promising candidates for carbon capture and utilization in the production of value‐added chemicals. Incorporating NHC complexes into the COF backbone has been challenging, but Huang and co‐workers developed a novel method to create an NHC‐based COF catalyst capable of efficiently converting CO_2_ into formic acid.^[^ ^118^ ^]^ For the synthesis of NHC‐based COF, the synthesis of imidazole‐functionalized Im‐COF‐2 was achieved through the acid‐catalyzed solvothermal reaction between 4,7‐bis(4‐formylbenzyl)‐1‐methyl‐1H‐benzimidazole (BFMBIm) and 1,3,5‐tris(4‐aminophenyl)benzene (TAPB). To obtain the NHC─CO_2_ decorated Im‐COF‐2, i.e., *N*‐heterocyclic carbene‐based NHC─CO_2_‐COF, the Im‐COF‐2 was further treated with dimethyl carbonate at 120 °C for 24 h. The subsequent reaction of NHC─CO_2_‐COF with [Cp*IrCl_2_]_2_ in acetonitrile at 60 °C for 12 h afforded the Ir‐NHC decorated Ir‐NHC─COF (Figure 11e).

The well‐engineered porous COF‐NHC─CO_2_ showed remarkable activity and selectivity in the hydrosilylation of CO_2_ and *N*‐formylation of amines with CO_2_, efficiently producing methanol and formamides. Ir‐NHC─COF showed remarkable catalytic activity, achieving high turnover frequency (TOF) up to 17244 h^−1^ and turnover number (TON) up to 27015 (Figure 11f), outperforming many state‐of‐the‐art catalysts. The *N*‐heterocyclic carbene present in Ir‐NHC─COFs increased the electron density on the central metal Ir(III) cations, resulting in superior performance and recyclability in CO_2_ hydrogenation to formate, outperforming metal‐free NHC─COFs by enhancing CO_2_ activation and enabling a more comprehensive range of catalytic activities, including multi‐electron transfer. The metal center in these COFs stabilizes intermediates, increases efficiency, lowers energy barriers, and offers greater versatility in catalytic pathways. In addition, Ir‐NHC‐based COFs provide better control of selectivity and are more stable and durable under reaction conditions, offering the potential to bridge the gap between homogeneous and heterogeneous catalysis.

Compared to artificial photocatalysts, natural enzymes such as formate dehydrogenase (FDH), CO_2_ reductase, CO dehydrogenase, remodeled nitrogenase, and carbonic anhydrase offer green alternatives for efficient CO_2_ reduction with superior efficiency and selectivity. However, large‐scale production, high cost, non‐reusability, and poor stability under harsh conditions are major concerns regarding the use of enzymes. Under these circumstances, the simultaneous integration of enzymes and NADH regeneration photocatalysts within the matrix of porous materials has been attempted to prepare heterogeneous cascade biocatalysts. Although many porous materials have been used, COFs, with their periodic and tunable pore arrangement and functionalization have emerged as particularly suited solid supports for biocatalysts.

The preparation of an electron mediator and FDH co‐anchored olefin‐linked mesoporous NKCOF‐113 (FDH@RhCp*‐NKCOF‐113) has recently been achieved. A solvothermal reaction between 2,4,6‐tris(4‐formylphenyl)‐1,3,5‐triazine and 5,5′‐bis(cyanomethyl)‐2,2′‐bipyridine in the presence of Cs_2_CO_3_ and suitable solvents yielded the olefin‐linked NKCOF‐113, which was further treated with dichloro(pentamethylcyclopentadienyl)rhodium(III) dimer to achieve the decoration of (Cp*Rh(bpy)H_2_O) in the COF framework.^[^ ^119^ ^]^ The energy level diagrams of pristine NKCOF‐113 and metal‐coordinated RhCp*‐NKCOF‐113 showed that these materials can provide protons for the photoreduction of NAD^+^ to NADH. Among the different combinations, RhCp*‐NKCOF‐113^d^ was found to be effective for NADH photoregeneration, accumulating up to 80% yield (20 min), which was found to be 4 times higher than that of pristine NKCOF‐113. Photocatalytic CO_2_ reduction was attempted using FDH@RhCp*‐NKCOF‐113 under 1 atm CO_2_ with visible light (λ>420 nm) at room temperature. The photocatalyst powder dispersed in 100 mL of 5 mM NAD^+^ in 10 mM phosphate buffer (pH 7.0) with 5 wt% triethanolamine showed the generation of 420 µg formic acid after 2.5 h, which is almost the same as free FDH. Direct addition of free FDH to the catalyst system resulted in deactivation of FDH due to its low stability. Interestingly, FDH@RhCp*‐NKCOF‐113a shows no significant loss of CO_2_ reduction activity after five cycles, indicating excellent photostability and recyclability, successfully demonstrating the immobilization of enzymes and electronic mediator in the COF matrix to form an integrated photocatalytic system.

Similarly, Jin‐Ook Baeg showed the structural composition within the COF backbone strongly influences the rate of photocatalytic CO_2_ reduction. It has been shown that hydroxyl groups near the imine bond induce a push‐pull effect, which enhances the photocatalytic performance. To analyze this effect, four different COFs were synthesized by varying the number of hydroxyl groups under solvothermal conditions by reacting the tripodal amine 4,4′,4″‐(1,3,5‐triazine‐2,4,6‐triyl)tris(([1, 1′‐biphenyl]‐4‐amine)) [Ttba] with equal moles of 1,3,5‐triformylbenzene, 2,4,6‐triformylphloroglucinol, 2,4,6‐triformylphenol or 2,4,6‐triformylresorcinol, respectively (Figure 11g).^[^ ^61^ ^]^ COF‐1 without hydroxyl groups doesn't show any keto‐enol formation, COF‐2 has three hydroxyl groups leading to irreversible keto‐enol formation, while COF‐3 and COF‐4, with two and one hydroxyl group(s) respectively, show reversible keto‐enol tautomerization. As confirmed by UV‐vis diffuse reflectance spectra, COF‐1 and COF‐2 show limited visible light absorption, while COF‐3 and COF‐4 absorb a wide range of visible light. As a result, COF‐3 and COF‐4 efficiently regenerated NADH (73.8% and 94.6%), yielding 170.4 and 226.3 µmol of formic acid by CO_2_ photoreduction in 90 min (Figure 11h), compared to which COF‐1 and COF‐2 showed lower efficiency. It has been shown that a fully conjugated system, the most suitable band gap and band edge positions, and higher crystallinity enhance the photocatalytic activity of COF‐4 compared to other counterparts, demonstrating the role of band gap engineering through rational design and fine‐tuning of building blocks in CO_2_ reduction.

### 4.3 Photocatalytic Organic Transformations

Photocatalytic organic transformations are an environmentally friendly and sustainable strategy that can be used to synthesize a range of value‐added pharmaceutical and industrial products. Typical homogeneous transition metal oxide photocatalysts suffer from toxicity, selectivity, moisture instability, and poor recyclability. Due to the advantageous properties of tunable building blocks, long‐range periodicity, and high porosity, COFs have also been successfully used as heterogeneous photocatalysts for organic transformation into valuable chemicals. COFs have been demonstrated in a variety of organic transformations such as C─H activation, oxidation, reduction, cross‐coupling reactions, isomerization, addition, and cyclization upon irradiation with UV/visible light, which are summarized in the following sections:

#### 4.3.1 C─H Functionalization

**Figure 12:**
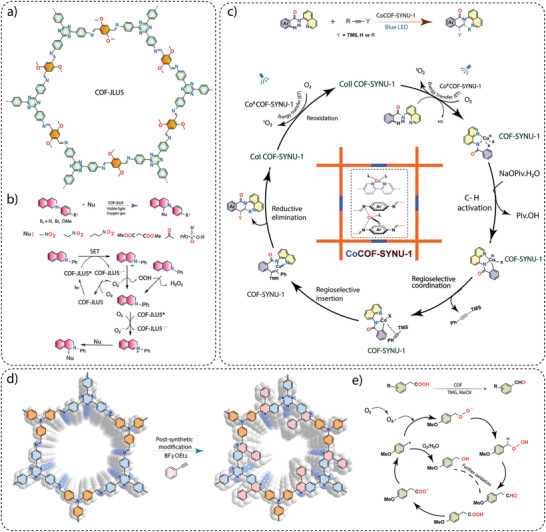
a) Scheme of the synthesis of COF‐JLU5. b) A plausible mechanism of aerobic C─H functionalization using COF‐JLU5. Reproduced with permission.^[^ ^122^ ^]^ Copyright 2017, Royal Society of Chemistry c) Scheme and proposed mechanism of C─H annulation of amides and alkynes promoted by CoCOF‐SYNU‐1. Reproduced with permission.^[^ ^125^ ^]^ Copyright 2022, Royal Society of Chemistry. d) Post‐synthetic modification strategy of triphenylamine incorporated COFs. e) A plausible mechanism of aerobic decarboxylation of aryl acetic acid using TFPA‐TAPT‐COF‐Q. Reproduced with permission.^[^ ^126^ ^]^ Copyright 2024, Springer Nature.

Activation of the less reactive and abundant C–H bond gives rise to many natural products, pharmaceuticals, agrochemicals, and materials with improved physico‐chemical properties and well‐defined structure‐activity relationships.^[^ ^120^, ^121^ ^]^ C–H activation, metalation and carbene insertion mostly require harsh acidic/basic reaction conditions, high temperatures, precise stoichiometric amounts and costly transition metal catalysts composed of Ru, Rh, and Pd. In 2017, Liu and co‐workers first used a layered two‐dimensional donor‐acceptor COF‐JLU5 as a heterogeneous photocatalyst for an aerobic cross‐dehydrogenative coupling (CDC) reaction (**Figure** **12**a).^[^ ^122^ ^]^ COF‐JLU5 was prepared solvothermally by Schiff‐base condensation between 1,3,5‐tris‐(4‐aminophenyl)triazine and 2,5‐dimethoxyterephthaldehyde. COF‐JLU5 embodied the donor‐acceptor combination that led to its considerable photocatalytic efficiency, enhanced by the π‐π conjugation and the planarity induced by the electron‐withdrawing triazine ring. On the other hand, the electron‐donating methoxy groups attributed to the chemical stability of the COF due to the interlayer and intralayer H‐bonding that stabilizes the COF layers.

The aerobic cross‐dehydrogenative coupling of *N*‐phenyl‐1,2,3,4‐tetrahydroisoquinoline (THIQ) with nitromethane was carried out in the presence of blue LEDs at 25 °C and oxygen using redox‐active COF‐JLU5, which exhibited an absorption band at 470 nm. COF‐JLU5 displayed a high yield (99%) with a fast conversion rate in the presence of methanol. It was observed that the superoxide radical anion abstracts a proton from the radical cationic form of the substrate, while the COF catalyst returns to the ground state. The proton abstracted form of the substrate undergoes electron loss followed by nucleophilic addition to give the desired product (Figure 12b). The recyclability and scalability experiments of the COF‐JLU5 photocatalyst showed reasonable conversions of 68% and 95%, respectively.

Cross dehydrogenative coupling reactions fall into the category of C─H functionalization, where the efficient formation of a C─C bond from two C─H bonds occurs without the need for pre‐activation. COFs with customizable building blocks can act as stable and highly reactive heterogeneous photocatalysts to carry out such organic transformations in a sustainable manner. In this regard, the cross‐dehydrogenative coupling reaction of *N*‐phenyl‐1,2,3,4‐THIQ with nitromethane was demonstrated by Wu and co‐workers using a hydrazone‐based COF (TFB‐COF).^[^ ^123^ ^]^ Hydrazone‐linked COFs are known for their dynamic nature of imine bond reversibility, hydrogen bonding through the amide group, hydrophilicity, and enhanced oxidative stability, making them ideal photocatalysts. In this work, hydrazone‐linked TFB‐COF was prepared by condensation of 2,5‐dimethoxyterephthalohydrazide (DMTH) with 1,3,5‐triformylbenzene (TFB) under solvothermal conditions. The cross‐dehydrogenative coupling reactions were carried out using nitromethane as the substrate in the presence of oxygen and a 45 W energy‐saving lamp as the energy source. A maximum yield of 87% was obtained with a catalyst loading of 30% and reusability of up to three cycles. The disturbed conjugation of the hydrazone bonds probably contributed to the comparatively lower conversion efficiency, which could be further increased by using substrates with electron‐donating groups in the para position as opposed to electron‐withdrawing groups. The cross‐dehydrogenative coupling reaction was also found to be applicable to the oxidative Mannich reaction between *N*‐phenyl THIQ with acetone and acetophenone with moderate yields.

Further, Chen and co‐workers used a donor‐acceptor type vinylene‐linked COF embedded with Ni to form C‐O bonds.^[^ ^124^ ^]^ Highly stable olefin‐linked sp^2^‐COF_dpy_‐Ni was synthesized via the Knoevenagel condensation reaction of 1,3,6,8‐tetrakis(4‐formyl phenyl) pyrene (TFPPy) and 2,2′‐([2,2′‐bipyridine]‐5,5′‐diyl) diacetonitrile (BPYDAN), following which Ni was incorporated at the bipyridine site by post‐synthetic modification to afford sp^2^c‐COF_dpy_‐Ni. The bipyridine moiety contributes to the chelation of the Ni active species and the prevention of Ni loss. The presence of extended π‐conjugation between the building blocks facilitates the transfer of photogenerated charge carriers to the metal catalytic site. The UV‐Vis diffuse reflectance spectra showed that the absorption edge for the donor‐acceptor‐based sp^2^c‐COF_dpy_‐Ni is extended to a broader absorption edge than that of sp^2^c‐COF_dpy_, reaching up to 800 nm due to the enhanced delocalization after Ni‐chelation. A narrower band gap at 1.86 eV was revealed for sp^2^c‐COF_dpy_‐Ni as determined from the Kubelka–Munk equation, confirming a possible visible solid light response. The photocatalytic experiments using sp^2^c‐COF_dpy_‐Ni confirmed that the etherification of 4‐bromobenzonitrile with methanol to the desired 4‐methoxybenzonitrile was obtained in a quantitative yield upon irradiation with a 6 W LEDs lamp (λ_max_ = 460–465 nm). Although the aryl chlorides were inert, the aryl bromides with para and meta substitution gave yields > 95%. The two‐dimensional Ni‐sp^2^_dpy_‐COF possessed high carrier mobility in the vertical direction, contributing to its moderately high performance as a heterogeneous photocatalyst. No apparent decrease in the yield after four repeated cycles was observed when Ni‐sp^2^_dpy_‐COF was recycled, demonstrating an environmentally friendly and sustainable pathway.

The combination of transition metal catalysis and photocatalysis provides a reassuring balance between effective chemical transformation and optimal use of light. Aiming to exploit this dual advantage and achieve chemo‐regio‐selectivity and sustainability, Yang and co‐workers developed a robust cobalt metal (Co)‐anchored COF, CoCOF‐SYNU‐1, for the C─H annulation of amides and alkynes under visible light irradiation, yielding a wide range of isoquinolin‐1(2H)‐one derivatives with high efficiency (Figure 12c).^[^ ^125^ ^]^ The synthesis of bipydine‐based COF (BPy‐COF) was achieved through the solvothermal reaction of 4,4′,4″,4‴‐(1,9‐dihydropyrene‐1,3,6,8‐tetrayl)tetraaniline and [2,2′‐bipyridine]‐5,5′‐dicarbaldehyde using acetic acid as a catalyst. To prepare a metal‐coordinated COF (CoCOF‐SYNU‐1), the BPy‐COF was refluxed with Co(OAc)_2_•4H_2_O in 2,2,2‐trifluoroethanol under an argon atmosphere. The chelated Co was found to be uniformly dispersed at 11.01 wt% and assists in the activation of the C─H bonds, while the photoactive COF framework facilitates the absorption of light. Under blue light irradiation, the photo‐excitons from the COF generate the singlet oxygen species. This is followed by the oxidation of Co(II)COF‐SYNU‐1 to give Co(III)COF‐SYNU‐1. The Co(III) species undergo coordination with benzamide and ligand exchange. The resulting intermediate promoted sequential coordination and migratory insertion with an alkyne, followed by reductive elimination to afford the desired product. Finally, Co(I)COF‐SYNU‐1 can be reoxidized to Co(II)COF‐SYNU‐1 using photogenerated singlet oxygen. Regardless of the electron donating or withdrawing groups, all substrates yielded more than 76% of the desired product, demonstrating the versatility of the photocatalyst. The activity of the Co(I) COF‐SYNU‐1 was maintained over six catalytic cycles, making it a reusable photocatalyst. This transition metal catalysis pushed the boundaries of visible light redox catalysis by effectively coordinating with the substrate and yielding a significant amount of C─H‐activated products.

In an effort to develop robust linkages beyond the most commonly used imines, Zhao and co‐workers recently demonstrated that imine‐linked COFs (TFPA‐TAPT‐COF) can be modified via a post‐synthetic strategy into more stable quinoline COFs (TFPA‐TAPT‐COF‐Q) (Figure 12d).^[^ ^126^ ^]^ Initially, tris(4‐formylphenyl)amine (TFPA) was chosen as the donor due to its nitrogen‐rich planar structure with enhanced π‐conjugation, which was reacted with the organic amine 1,3,5‐tris(4‐aminophenyl)triazine (TAPT) as the acceptor moiety to yield TFPA‐TAPT COF. The crystalline and porous COF was further modified into a quinoline‐linked TFPA‐TAPT‐COF‐Q using the Povarov reaction to create a donor‐π‐acceptor system with enhanced interlayer interaction. The crystalline, porous, thermally and chemically stable TFPA‐TAPT‐COF‐Q exhibited a wider absorption window of 250–600 nm and superior photochemical properties, surpassing the original imine‐linked COFs. In the presence of triphenylamine as the photoabsorber moiety, the more stable quinoline‐linked TFPA‐TAPT‐COF‐Q exhibited enhanced photocatalytic performance for the oxidative decarboxylation of arylacetic acid in the presence of 1,1,3,3‐tetramethylguanidine (TMG) as a cocatalyst, resulting in complete conversion after 9 h (Figure 12e). The TFPA‐TAPT‐COF‐Q was instrumental in the generation of superoxide radical anion (^•^O_2_^¯^), which mediated the photocatalytic decarboxylation reactions. The quinoline‐linked COFs exhibited effective conversion of benzylamine to benzaldehyde in the presence of acetonitrile while maintaining stability, good photocatalytic activity up to five cycles, and remarkable substrate tolerance.

#### 4.3.2 Oxidation and Reduction

**Figure 13:**
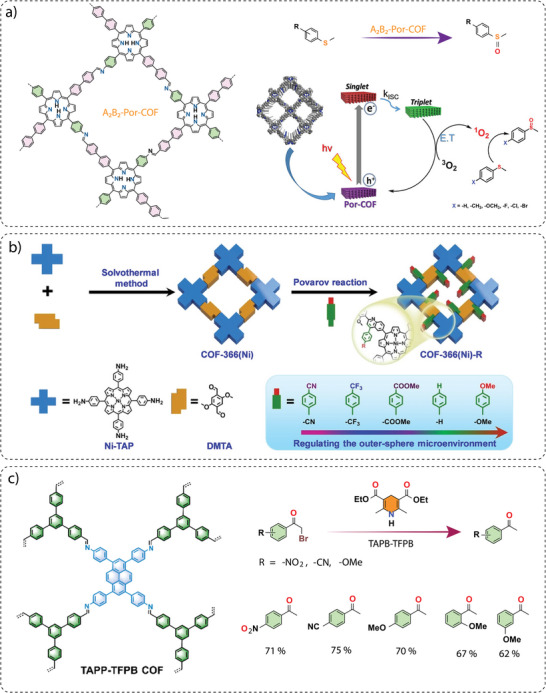
a) Schematic representation of the A_2_B_2_‐Por‐COF and the single electron transfer (SET) processes involved in the oxidation reaction of thioanisole. Reproduced with permission.^[^ ^128^ ^]^ Copyright 2019, American Chemical Society. b) Outer sphere microenvironment modulation of Ni sites in COF‐366(Ni)‐R (─R═, ─CN, ─CF_3_, ─COOMe, ─H, ─OMe) for photocatalytic oxidation of thioether. Reproduced with permission.^[^ ^129^ ^]^ Copyright 2024, Wiley‐VCH. c) Chemical structure of TAPP‐TFPB COF and scheme of photoreductive debromination catalyzed by TAPB‐TFPB COF. Reproduced with permission.^[^ ^130^ ^]^ Copyright 2024, American Chemical Society.

Oxidation and reduction are among the most important organic transformations, involving the formation of epoxides, acetaldehyde from double bonds, the conversion of alcohol to aldehyde, or the formation of sulfoxides from thioethers. However, these reactions suffer from low regioselectivity and chemoselectivity, as well as the difficulty of liberating waste products from oxidants such as K_2_Cr_2_O_7_ or KMnO_4_.^[^ ^127^ ^]^ In this regard, the porphyrin unit, with interesting photophysical and redox properties, together with the enhanced π‐conjugation, has been shown to transfer the energy extracted from the photosensitizing units to the molecular oxygen, generating the singlet oxygen species. Considering these advantages, Chen and co‐workers synthesized a bifunctional A_2_B_2_‐type porphyrin‐based COF with identical amino and formyl functionalities at the opposite meso positions (**Figure** **13**a).^[^ ^128^ ^]^ The crystalline, porous, and chemically stable COF was obtained by self‐condensation of A_2_B_2_‐Por(4′,4‴‐(10,20‐bis(4‐aminophenyl)porphyrin‐5,15‐diyl)bis‐(([1,1′‐biphenyl]‐4‐carbaldehyde)) under solvothermal conditions. The porphyrin‐based COF was used as a photocatalyst for the heterogeneous catalysis of thioanisoles to mono‐oxidized sulfoxides with 98% conversion efficiency, 99% selectivity, stability, and recyclability. The metal‐free porphyrin moieties provided the COF with light absorption capacity and rich photoelectric properties. These properties were critical for the generation of the much‐needed singlet oxygen species for the energy transfer pathways.

Singlet oxygen (^1^O_2_) is essential for photocatalytic oxidation reactions, but the selective generation of singlet oxygen species is a challenge. Although heavy atom‐based photosensitizer units and organic dyes have been used to generate singlet oxygen, these materials face poor selectivity, toxicity, and photobleaching. One effective strategy is to modify the coordination environment around the catalytic site. To address this issue, Jiang and co‐workers used a Ni‐porphyrin COF for outer‐sphere microenvironment modulation to generate singlet oxygen ^1^O_2_, a neutral species with a long lifetime and good oxidizing properties.^[^ ^129^ ^]^ The outer sphere microenvironment is influenced by the functionalities of the active sites, which alter the catalytic performance. A series of isoreticular COF‐366(Ni) with different functionalities have been prepared by the solvothermal method through condensation between 5,10,15,20‐tetrakis(4‐aminophenyl)porphinato] nickel (Ni‐TAP) and 2,5‐dimethoxyterephthaldehyde (DMTA) using 1,2‐dichlorobenzene (o‐DCB) and butanol mixture in the presence of aqueous acetic acid. The electron‐donating and withdrawing groups such as ‐CN, ‐CF_3_, ‐COOMe, ‐H, and ‐OMe were decorated on the pore walls via the Povarov reaction applying a post‐synthetic modification strategy. The photocatalytic reactions carried out using a violet light emitting diode (410‐420 nm) in acetonitrile and in the presence of oxygen showed that the electron‐donating groups have a better impact on catalytic activity, exhibiting a higher catalytic activity with a maximum yield of 98% for the oxidation of thioethers when the methoxy‐substituted COF was used (Figure 13b). The enhanced light‐harvesting and photoelectric properties resulting from the functionalization of the pore walls are responsible for the photocatalytic activity for the oxidation of thioanisoles.

COFs have been applied to various processes such as dehydrogenation, oxidative amine coupling, thioamide cyclization and dehalogenation, but the same COF that can catalyze both photocatalytic oxidation and photocatalytic reduction is still to be found. To address this, Verduzco and co‐workers demonstrated the synthesis of COFs consisting of either a tetra(biphenyl)ethene building block or a pyrene building block, termed TAPB‐ETTBC and TAPP‐TFPB, respectively (Figure 13c).^[^ ^130^ ^]^ TAPB‐ETTBC COF was prepared by the Schiff‐base reaction of a stoichiometric ratio of trigonal 1,3,5‐tris(4‐aminophenyl)benzene (TAPB) and square 4′,4‴,4‴″,4‴″‐(ethene‐1,1,2,2‐tetrayl)tetrakis([1,1′‐biphenyl]‐4‐carbaldehyde) (ETTBC), while TAPP‐TFPB COF was synthesized by the Schiff base reaction of trigonal 1,3,5‐tri(4‐formylphenyl)benzene (TFPB) and square 1,3,6,8‐tetrakis(4‐aminophenyl)pyrene (TAPP). These COFs were applied to the photocatalytic dehalogenation reactions for the degradation of hazardous pollutants as well as the generation of radical‐centered intermediates for the pharmaceutical industry. A moderate yield of 78% was achieved with the TAPP‐TFPB COF. The yield of the desired product remained unchanged with electron‐donating and electron‐withdrawing substituents. The chain‐growth radical polymerization of methyl methacrylate was also investigated using the same COF, where a yield of 70.3% monomer conversion was observed.

#### 4.3.3 Cross‐Coupling Reactions

**Figure 14:**
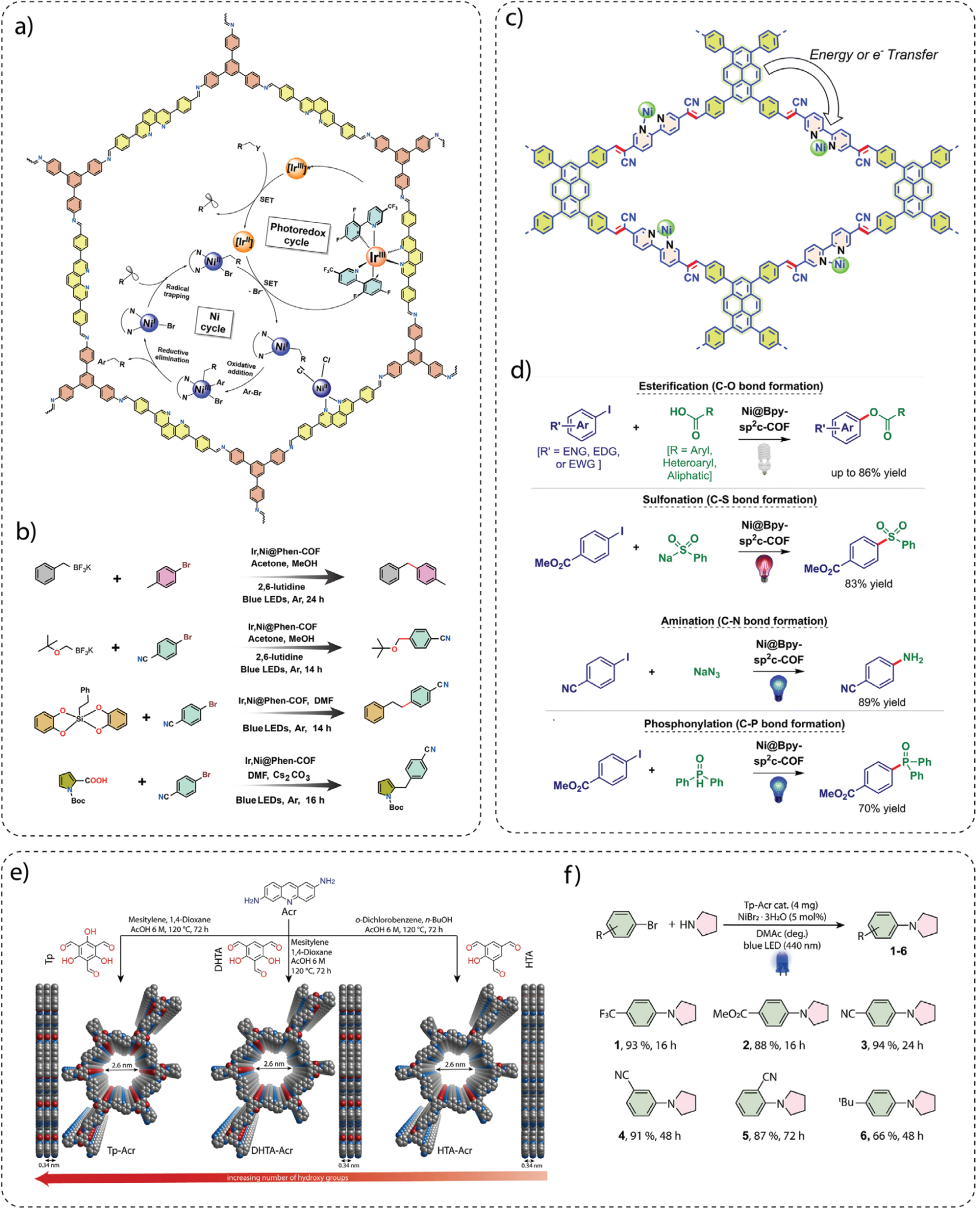
a) Synthesis of Ir‐ and Ni‐containing phenanthroline COF having two catalytic sites. b) C(sp^3^)–C(sp^2^) cross‐coupling reactions promoted by Ir,Ni@Phen‐COF. Reproduced with permission.^[^ ^131^ ^]^ Copyright 2021, American Chemical Society c) Single metal‐embedded Ni@Bpy‐sp^2^ c‐COF. d) Diverse photocatalytic C–X (X = B, C, N, O, P, S) cross‐coupling reactions using Ni@Bpy‐sp^2^‐c‐COF. Reproduced with permission.^[^ ^132^ ^]^ Copyright 2023, Royal Society of Chemistry. e) Scheme of the synthesis of Tp‐Acr, DHTA‐Acr and HTA‐Acr COFs. f) Scope of C‐N cross‐coupling reaction using Ni‐embedded acridine‐based COFs. Reproduced with permission.^[^ ^133^ ^]^ Copyright 2022, Wiley‐VCH.

Carbon‐carbon and carbon‐heteroatom cross‐coupling reactions are of great importance in the synthesis of novel compounds through bond activation and cleavage and are, therefore, in great demand in the organic synthesis of chemicals and pharmaceuticals. The limitations of homogeneous catalysis led Mas‐Ballesté and colleagues to investigate solar‐driven C─C coupling reactions under environmentally benign and mild conditions.^[^ ^131^ ^]^ The pristine Phen‐COF, consisting of a phenanthroline backbone, was prepared by a solvothermal reaction of a symmetrical building block containing a central phenanthroline unit with two aldehyde groups at its ends with tris(4‐aminophenyl)benzene. Post‐functionalization to immobilize two metals, Ir and Ni, was achieved using [dF(CF_3_)ppy)_2_‐Ir‐µ‐Cl]_2_ and NiCl_2_ as precursors, respectively. Due to the low band gap of 2.29 eV and the proximally located metal active sites, Ir, Ni@Phen COF resulted in high reactivity and selectivity in the cross‐coupling of aryl bromides and potassium benzyl/alkoxy trifluoroborate salts, organic silicates and N‐protected proline with a photocatalytic efficiency of 90% (**Figure** **14**a‐b).^[^ ^131^ ^]^ The proximal metal active sites provided synchronized yet distinct catalytic cycles for the cross‐coupling reactions while maintaining high activity and selectivity. The optimal ratio of Ni and Ir was found to be 2:1 for the activation of the substrate, which was supported by the maximum turnover number with considerable yield.

Vinylene‐linked COFs are characterized by their extended conjugation and remarkable chemical stability. With the aim of exploiting these properties, Maji and co‐workers prepared a Ni@Bpy‐sp^2^c COF that could effectively lead to high yields of C‐X (X = N, O, S, P) cross‐coupled products (Figure 14c).^[^ ^132^ ^]^ A vinylene‐linked crystalline and porous COF with bipyridine moieties (Bpy‐sp^2^c‐COF) was synthesized via the Knoevenagel condensation reaction between 1,3,6,8‐tetrakis(4‐formylphenyl)pyrene (TFPPy) and 5,5′ ‐diacetonitrile‐2,2′ ‐bipyridine (BPDAN). Further, Bpy‐sp^2^c‐COF was then treated with NiCl_2_ to obtain non‐toxic, single metal‐embedded, and π‐conjugated Ni@Bpy‐sp^2^c‐COF. When applied to cross‐coupling reactions, the percentage of conversion reached values up to 86% for C‐O coupling and 83% for C‐S coupled products in the presence of 2,6‐lutidine in acetonitrile under irradiation of a blue light emitting diode (427 nm). Product yields were slightly lower at 70% and 61% for C‐P and C─C coupled products, respectively, under the same conditions. The selective coupling of reactants with different steric and electronic properties was achieved by the synergistic photo‐redox properties of the Ni active sites anchored to the photosensitive COF backbone with appropriately aligned valence and conduction bands (Figure 14d).

Among the cross‐coupling reactions, C‐N cross‐coupling contributes to the synthesis of several important pharmaceuticals and drugs. Conventional C‐N coupling involves the use of toxic heavy metals, which pose a threat to the environment. To address this challenge, we have demonstrated heterogeneous and sustainable carbon‐heteroatom (C‐N) cross‐coupling using a series of acridine‐based COFs, such as Tp‐Acr, DHTA‐Acr, and HTA‐Acr, supported by a Ni metallophotocatalytic site, opening new horizons in the field of metallo‐photoredox catalysis (Figure 14e).^[^ ^133^ ^]^ The synthesis of Tp‐Acr, DHTA‐Acr and HTA‐Acr COFs was achieved by a solvothermal reaction of 2,6‐diaminoacridine (Acr) with 1,3,5‐triformylphloroglucinol (Tp), 2,4‐dihydroxybenzene‐1,3,5‐tricarbaldehyde (DHTA) and 2‐hydroxybenzene‐1,3,5‐tricarbaldehyde (HTA), respectively. A different ratio of *β*‐ketoenamine to imine in Tp‐Acr, DHTA‐Acr, and HTA‐Acr COFs was achieved by changing the number of hydroxy groups. To achieve the photocatalytic cross‐coupling reaction, NiBr_2_⋅3H_2_O (5 mol%) was added to the acridine‐based COFs. The acridine moieties endowed the COF photocatalyst with remarkable light‐harvesting properties, while the Ni center was essential for achieving charge transfer capabilities. When used for photocatalytic organic transformation, the fully *β*‐ketoenamine tautomerized Tp‐Acr COF with the maximum number of hydroxyl groups showed a higher yield (up to 91%) for the coupling reaction between 4‐bromobenzotrifluoride with pyrrolidine under blue LED light (440 nm) in 16 h, compared to DHTA‐Acr and HTA‐Acr COFs (Figure 14f). A higher surface area compared to the other COFs, together with the favorable electron/hole transfer kinetics afforded by a planar COF structure, are the factors responsible for the enhanced catalytic activity of the Tp‐Acr COF. TP‐Acr COFs/Ni also readily facilitated C‐N coupling products under the green light in conjunction with Ni(II) salt. Although acridine‐based COFs showed good recyclability, the semi‐heterogeneous nature of the system, using the COF as solid photosensitizer and the Ni‐catalyst in solution, required that the nickel catalyst had to be added in every new cycle.

**Figure 15:**
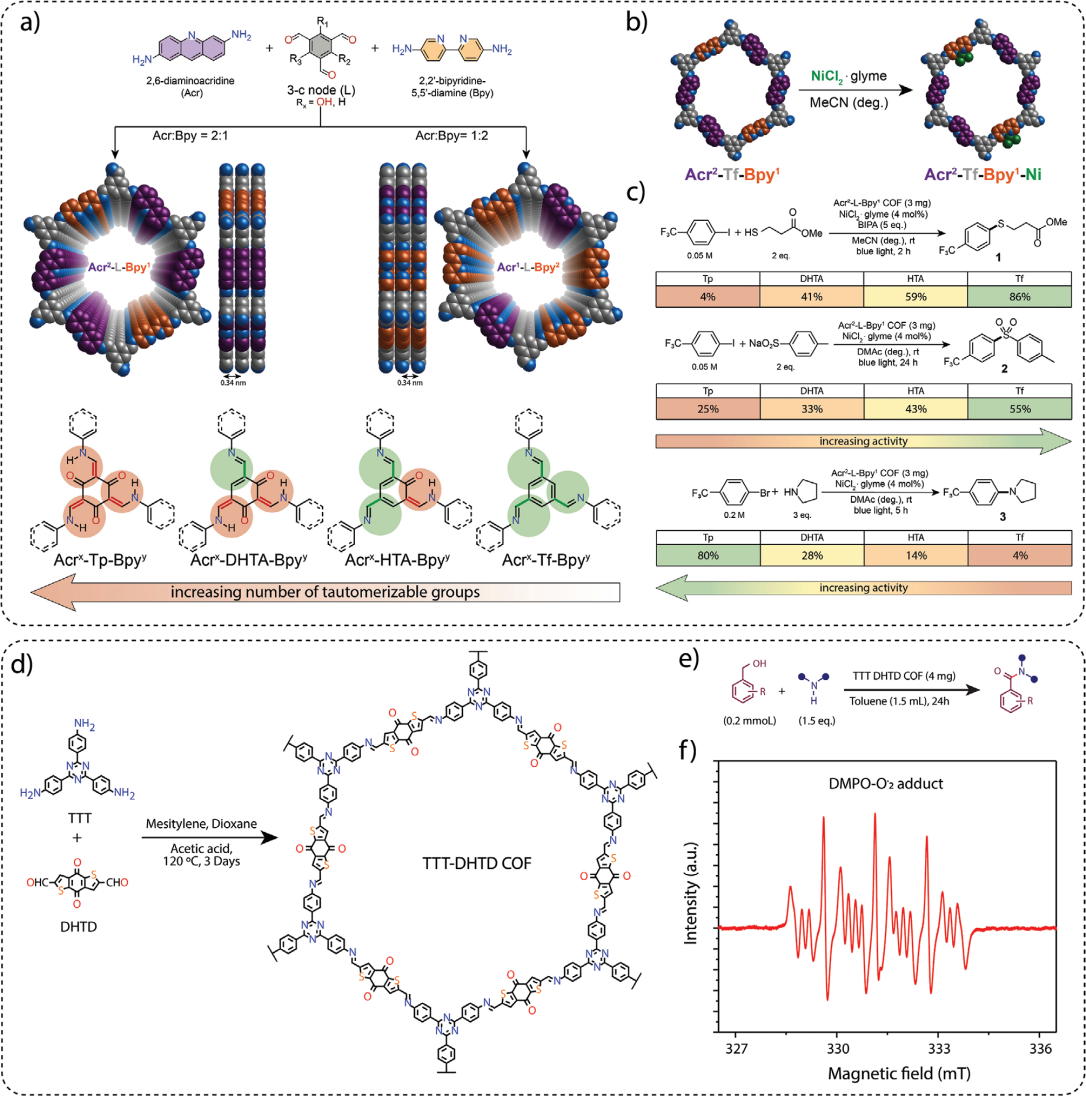
Scheme of the synthesis of multicomponent COFs with a variable number of hydroxyl groups (a) and Ni‐embedded multicomponent COFs (b). c) Photocatalytic performances in different cross‐coupling reactions using Ni‐embedded multicomponent COFs. Reproduced with permission.^[^ ^134^ ^]^ Copyright 2023, Wiley‐VCH. d) The scheme of the synthesis of TTT‐DMTD COF for the cross‐dehydrogenative coupling alcohol to amide. e) The model amidation reaction. f) Confirmation of the formation of DMPO‐^•^O_2_¯ adduct by EPR analysis of TTT‐DHTD. Reproduced with permission.^[^ ^137^ ^]^ Copyright 2024, Wiley‐VCH.

To overcome the concerns regarding the semi‐heterogeneous system, multicomponent COFs were synthesized combining the acridine photosensitizing unit, with a bipyridine ligand for immobilizing the Ni‐catalyst. This was achieved by a solvothermal reaction of 1,3,5‐benzenetrialdehyde derivatives with 2,6‐acridinediamine (Acr) and 2,2′‐bipyridine‐5,5′‐diamine (Bpy) in Acr/Bpy ratios of 2:1 and 1:2 (**Figure** **15**a).^[^ ^134^ ^]^ Subsequently, ex situ nickel coordination with Acr^2^‐Tf‐Bpy^1^ was achieved by refluxing a suspension of COF and NiCl_2_•glyme in acetonitrile (Figure 15b). The photocatalytic efficiencies of the Ni‐embedded COFs were analyzed for various photocatalytic carbon‐heteroatom cross‐coupling reactions over a range of substrates under blue LED. The C–O, C–N, and C–S cross‐coupling reactions are followed via energy transfer pathways, where it was observed that light‐mediated carbon‐heteroatom cross‐coupling could be achieved in good yields (Figure 15c). The interesting feature of the Ni‐embedded multicomponent and fully heterogenous COFs was the prevention of Ni‐black formation and leaching, which allowed selective cross‐coupling with different electronic and steric properties with high photocatalytic efficiency and reusability.

In a similar effort, Maji and co‐workers carried out the dual metalation with crystalline and porous TpBpy COF, synthesized by solvothermal reaction of 1,3,5‐triformylphloroglucinol (Tp) and a 5,5′‐diamino‐2,2′‐bipyridine (Bpy), to obtain a bimetallated hybrid material containing [Ir(ppy)_2_(CH_3_CN)_2_]PF_6_ [ppy: 2‐phenylpyridine] and NiCl_2_ by post‐synthetic modification.^[^ ^135^ ^]^ The metal anchoring within the COF framework was enabled by the chelating N‐atoms of the bipyridine units. The photocatalytic performance of the resulting Ni‐Ir@TpBpy COF was investigated over a variety of substrates for the C‐N cross‐coupling reaction between (hetero)aryl iodides and amines (aryl, heteroaryl, alkyl) under irradiation with visible light. The coupling reaction of iodobenzene with aniline using Ni‐Ir@TpBpy photocatalyst resulted in a remarkable yield of 89%. By using a chelated metal complex, the formation of Ni‐black was prevented and the yield was maintained up to 78% after ten photocatalytic cycles.

In many cases, metallated COF photocatalysts suffer from the likelihood of degradation under visible light. In addition, organometallic compounds such as organoboranes result in lower yields due to poor functional group tolerance. To address these concerns simultaneously, Banerjee and co‐workers demonstrated the coupling between 4‐methylquinolone and trimethylborane using *β*‐ketoenamine‐linked COFs (Tp‐Azo, Tp‐Dpp, and Tp‐Tab), which exhibited remarkable absorption in the visible region.^[^ ^136^ ^]^ Tp‐Azo, Tp‐Dpp and Tp‐Tab COFs were synthesized by the condensation reaction of 1,3,5‐triformylphloroglucinol (Tp) with diamine‐(4,4′‐azodianiline) (Azo), 2,8‐diamino(6‐phenylphenanthridine) (Dpp) and triamine‐[1,3,5‐tris(4‐aminophenyl)benzene (Tap), respectively, via the Schiff base reaction under optimized solvent conditions. Among these COFs, Tp‐azo, upon reaction with hydroxyquinoline, led to the generation of the sought‐after organoborane compounds with 96% conversion efficiency, broad substrate range, and good recyclability. In addition, C─H borylation reactions were carried out using persulfate as a sacrificial electron acceptor. The efficient photocatalysis combined with broad substrate compatibility could be attributed to the large surface area, pore width, and effective charge separation and migration throughout the COF matrix.

Amide bonds are ubiquitous in biologically active molecules such as proteins, but their synthesis from primary amines faces competition from the generation of imines. The direct generation of amides from alcohols using homogeneous systems is rather rare due to synthetic difficulties. As a result, the search for heterogeneous catalytic systems capable of sustainable amide synthesis is gaining research interest. Recently, some of us have developed a recyclable, sustainable and efficient dithiophene‐based COF photocatalyst for the red light‐assisted synthesis of amides directly from alcohols via cross‐dehydrogenative coupling.^[^ ^137^ ^]^ The TTT‐DHTD COF was constructed by a solvothermal reaction of 4,4′,4′′‐(1,3,5‐triazine‐2,4,6‐triyl)trianiline (TTT) with 4,8‐dioxo‐4,8‐dihydrobenzo[1,2‐b:4,5‐b']dithiophene‐2,6‐dicarbaldehyde (DHTD) (Figure 15d). The crystalline and porous TTT‐DHTD COF with high‐density dithiophenedione functional units exhibited light absorption over the entire visible range, a narrow band gap and suitable valence band positions, making it well suited for efficient exciton generation and providing an ideal platform for heterogeneous amide synthesis. Amide synthesis from alcohols and amines using TTT‐DHTD COF as photocatalyst under red light irradiation at room temperature showed 85% yield of the desired product with no significant decrease in catalytic activity after 10 cycles, with toluene as the most suitable solvent (Figure 15e). This can be attributed to the narrow band gap, appropriately located valence band position and highly reducible photochromic moiety. The dithiophene‐benzoquinone based motif provides a redox active site for carrier harvesting and facilitates the generation of superoxide radical anion (Figure 15f). The well‐aligned one‐dimensional channels also provide a fertile ground for dehydrogenation, eliminating the need for a cocatalyst – providing a viable transition metal‐free system for targeted solutions.

Oxidative coupling is another arena of photocatalyzed coupling reactions in which amines (usually two benzylic amines), one acting as an electrophile and the other as a nucleophile, are coupled to form imines under controlled conditions and without the need for dehydrating agents. Relying on the high chemical stability of the C═C bonds, Wang and colleagues investigated a novel two‐dimensional porphyrin‐based sp^2^‐carbon‐conjugated COF, named Por‐sp^2^c‐COF.^[^ ^138^ ^]^ The aerobic oxidation of amines to imines was promoted using a metal‐free, highly efficient heterogeneous photocatalyst. The inherent structure of Por‐sp^2^c‐COF has several advantages over the imine‐linked Por‐COF analog, including better conjugation and chemical stability – crucial properties for photocatalysis performance. In a similar effort, a combination of crystallinity and cooperative catalysis of TEMPO (2,2,6,6‐tetramethyl‐1‐piperidinyloxy) was carried out by Lang and co‐workers through the generation of Por‐sp^2^c‐COF, which was synthesized by the Knoevenagel condensation reaction of 5,10,15,20‐tetrakis(4‐benzaldehyde)porphyrin (p‐Por‐CHO) with 1,4‐phenylenediacetonitrile (PDAN) in an optimized ratio.^[^ ^139^ ^]^ The suitable size of TEMPO allowed it to be easily incorporated into the pores of the COF matrix, giving the system advantageous oxidizing and reducing properties. The Por‐sp^2^c‐COF showed better photocatalytic activities towards the oxidative coupling of amines than the Por‐COF. Only 2% TEMPO was sufficient to achieve 90% conversion, high selectivity and broad substrate compatibility. The substrate compatibility was extended to secondary amines and high turnover rates were obtained. The involvement of the superoxide radical anion in the plausible mechanistic pathway was confirmed using DMPO (5,5‐dimethyl‐1‐pyrroline‐*N*‐oxide) as a scavenger. The cooperative catalysis of COF and TEMPO was supported by effective electron‐hole separation and linker‐to‐linker charge transfer from the HOMO to the LUMO of the Por‐sp^2^C─COF, facilitated by the favorably positioned TEMPO.

### 4.4 Photocatalytic Hydrogen Peroxide (H_2_O_2_) Generation

Among the various valuable chemicals synthesized by photosynthesis, hydrogen peroxide (H_2_O_2_) has probably attracted the most attention in recent years. H_2_O_2_ finds diverse applications, ranging from a potent oxidizing agent to wastewater treatment, environmental remediation, healthcare, green fuel, and electronics.^[^ ^140^ ^]^ Compared to H_2_ as fuel, H_2_O_2_ has certain advantages such as easy storage and transportation and high solubility in water, making it a promising alternative energy source.^[^ ^141^ ^]^ The traditional anthraquinone oxidation (AQ) process for H_2_O_2_ production is increasingly being scrutinized for its inherent drawbacks, including the demand for a noble palladium catalyst, multiple operating steps, excessive energy consumption, the use of hazardous substrates, and the formation of large amounts of by‐products.^[^ ^142^ ^]^ Recognizing these limitations, researchers have focused on finding alternative, sustainable, and greener strategies for producing H_2_O_2_ to minimize the environmental impact. In this context, photosynthesis of H_2_O_2_ has emerged as a convincing alternative, offering the prospect of harnessing solar energy directly to drive chemical reactions, thereby avoiding the need for energy‐intensive and expensive materials. Among a variety of materials, well‐defined MOFs and COFs have received increasing research attention in photocatalytic H_2_O_2_ production,^[^ ^143^ ^]^ however, MOFs suffer from low ionic mobility and relatively low chemical stability as well as catalytic performance.^[^ ^45^, ^144^ ^]^ Hence, the following section summarizes the applications of COFs for photocatalytic H_2_O_2_ generation:

#### 4.4.1 Metal‐free COFs as Photocatalysts

**Figure 16:**
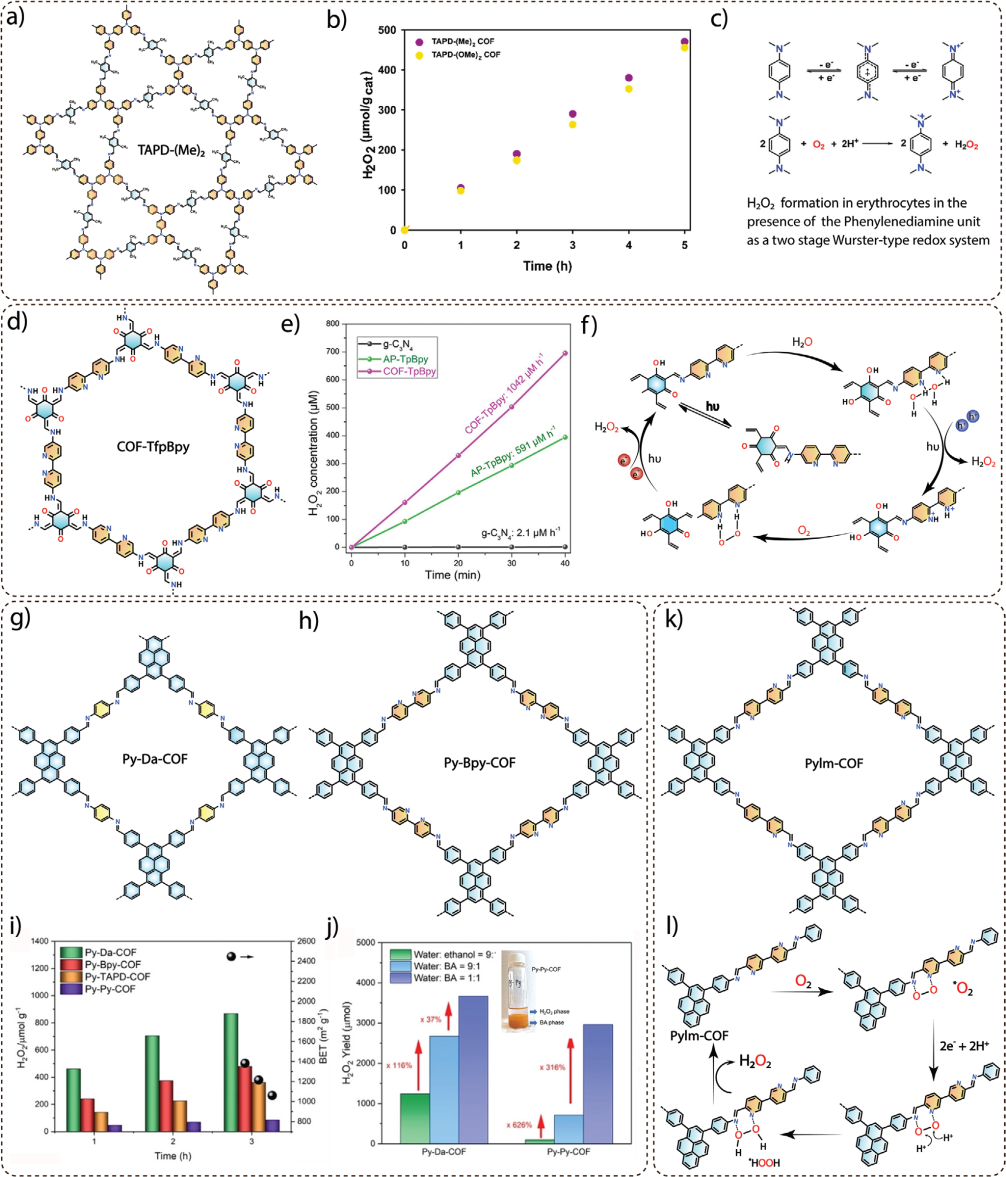
a) Structure of TAPD‐(Me)_2_ used for photocatalytic H_2_O_2_ generation. b) H_2_O_2_ generation using TAPD‐(Me)_2_ and TAPD‐(OMe)_2_ COFs. c) Possible H_2_O_2_ generation in erythrocytes using TMPD as a Wurster‐type redox system. Reproduced with permission.^[^ ^145^ ^]^ Copyright 2020, American Chemical Society. d–f) COF‐TfpBpy and its photocatalytic performance and possible mechanism for H_2_O_2_ generation. Reproduced with permission.^[^ ^146^ ^]^ Copyright 2022, Wiley‐VCH. g–j) Pyrene‐based Py‐Da‐COF and Py‐Bpy‐COFs and their photocatalytic H_2_O_2_ generation. Reproduced with permission.^[^ ^147^ ^]^ Copyright 2023, Wiley‐VCH. k) Pyrene‐based COF with Pyridyl‐Imine Structures used for H_2_O_2_ generation l) The proposed mechanism of H_2_O_2_ generation using PyIm‐COF via direct single‐step 2e¯ oxygen reduction pathway. Reproduced with permission.^[^ ^150^ ^]^ Copyright 2024, Wiley‐VCH.

In 2020, Van Der Voort and co‐workers first revealed the photocatalytic application of COFs in H_2_O_2_ generation using ethanol as a sacrificial agent.^[^ ^145^ ^]^ Two‐dimensional (2D) imine‐linked COFs, namely TAPD‐(Me)_2_ and TAPD‐(OMe)_2_ with Kagome (*kgm*) topology were synthesized by reacting electron‐rich *N,N,N′,N′*‐tetrakis(4‐aminophenyl)‐1,4‐phenylenediamine (TAPD) with 2,5‐dimethylbenzene‐1,4‐dicarboxaldehyde or 2,5‐dimethoxybenzene‐1,4‐dicarboxaldehyde, respectively (**Figure** **16**a). The resulting TAPD‐(Me)_2_ COF exhibited photocatalytic activity towards H_2_O_2_ generation with a production rate of 91–234 µmol h^−1^ g^−1^ from a water/ethanol mixture under visible light irradiation (Figure 16b). The redox TAPD moiety as a Wurster‐type system is essential in biological processes where it can scavenge molecular oxygen to form superoxide radical anion (^•^O_2_^¯^) to generate H_2_O_2_ (Figure 16c).

It has long been known that bipyridine acts as both a core reactive site and a proton acceptor unit due to the electron‐deficient nature of the N‐heteroatom. Considering these properties, Ma and co‐workers studied the potential application of a bipyridine‐based COF (denoted as COF‐TfpBpy) synthesized from 1,3,5‐triformylphloroglucinol (Tfp) and 2,2′‐bipyridine‐5,5′‐diamine (Bpy) for photocatalytic H_2_O_2_ generation (Figure 16d).^[^ ^146^ ^]^ The bipyridine‐incorporated COF (COF‐TfpBpy) exhibited a significantly higher H_2_O_2_ generation rate of 1042 µmol h^−1^ g^−1^ in pure water without using a sacrificial electron donor, which was significantly higher compared to the amorphous AP‐TfpBpy or non‐bipyridine based COF (COF‐TfyDaaq) (Figure 16e). The mechanism studies showed that the protonation of the N‐atom in the bipyridine moiety allowed the generation of H_2_O_2_ through single step 2e^¯^ redox reactions (Figure 16f). This work also demonstrated for the first time that the COF‐based photocatalyst can generate H_2_O_2_ from pure water and air without using any sacrificial reagent or stabilizer.

In parallel, pyrene as a strong donor moiety has received increasing attention in designing high‐performance COF‐based photocatalysts due to its extended π‐conjugation system, symmetrical tetraphenyl ring structure, and strong photoactivity. Taking this into account, Van Der Voort and co‐workers synthesized a set of four imine‐linked COFs with pyrene units, such as Py‐Da‐COF (Figure 16g), Py‐Bpy‐COF (Figure 16h), Py‐TAPD‐COF and Py‐Py‐COF) and studied their efficiency for photocatalytic H_2_O_2_ generation.^[^ ^147^ ^]^ These pyrene‐based COFs were synthesized by the Schiff‐base reaction between 1,3,6,8‐tetra(4‐formylphenyl)pyrene (Py‐CHO) with 1,4‐diaminobenzene (Da‐NH_2_), 2,2′‐bipyridine‐5,5′‐diamine (Bpy‐NH_2_), *N,N,N′,N′*‐tetrakis(4‐aminophenyl)‐1,4‐phenylenediamine (TAPD) or 4,4′,4″,4′′‐(pyrene‐1,3,6,8‐tetrayl)tetraaniline (Py‐NH_2_) to yield the respective Py‐Da‐COF, Py‐Bpy‐COF, Py‐TAPD‐COF, and Py‐Py‐COF, respectively. The photocatalytic H_2_O_2_ generation in one hour was found to be 461 µmol g^−1^ for benzene‐cored Py‐Da‐COF, 241 µmol g^−1^ for bipyridine‐incorporated Py‐Bpy‐COF, 142 µmol g^−1^ for Py‐TAPD‐COF containing phenylenediamine (TAPD), and 47 µmol g^−1^ for fully pyrene‐based Py‐Py‐COF in O_2_‐saturated water without any sacrificial agent (Figure 16i). The better photocatalytic activity of Py‐Da‐COF for H_2_O_2_ generation compared to the other three pyrene COFs was attributed to the higher surface area of Py‐Da‐COF. Despite having a higher number of pyrene units in close proximity in the Py‐Py‐COF framework, the lowest H_2_O_2_ generation rate was observed due to the significant decomposition of the H_2_O_2_ produced. To overcome this problem, a biphasic system of benzyl alcohol and water has been used, which allows the produced H_2_O_2_ to diffuse into the water layer, preventing the H_2_O_2_ decomposition by the catalyst (Figure 16j inset). Consequently, the photocatalytic performance of Py‐Py‐COF was increased by 6‐fold in the two‐phase system compared to that in the single‐phase water‐ethanol system (Figure 16j).

These works showed that COFs bearing pyrene or bipyridine moiety are beneficial for efficient H_2_O_2_ generation. However, due to the presence of imine nitrogen (C = N) in the bipyridine‐incorporated COFs, some O_2_ molecules tend to adsorb on the individual N sites, thus altering the reaction pathway of H_2_O_2_ generation from a one‐step 2e^−^ ORR (O_2_ + 2e^¯^ + 2H^+^ → H_2_O_2_) to a two‐step 2e^−^ ORR (O_2_ + e^¯^ → ^•^O_2_^¯^ + e^¯^ + 2H^+^ → H_2_O_2_).^[^ ^148^ ^]^ As a result, the direct two‐step 2e^¯^ ORR was significantly suppressed, resulting in reduced H_2_O_2_ production.^[^ ^149^ ^]^ To gain more insights into this process, Zhao and co‐workers recently rationally designed a set of three COFs, BD‐COF, Bpy‐COF and PyIm‐COF, with biphenyl, 2,2′‐bipyridine and 3,3′‐bipyridine units, respectively, for photocatalytic H_2_O_2_ generation.^[^ ^150^ ^]^ Interestingly, the PyIm‐COF bearing 3,3′‐bipyridine units exhibited a significantly higher H_2_O_2_ generation rate of 5850 µmol g^−1^ h^−1^ under visible light irradiation without sacrificial electron donors, which was almost twice as high as the pristine bipyridine COFs (3060 µmol g^−1^ h^−1^) and the BD‐COFs with biphenyl (670 µmol g^−1^ h^−1^). The unique architecture of the imine‐based PyIm‐COF offered pyridyl‐imine scaffolds with two adjacent N atoms between the bipyridine moiety and the imine linkage (Figure 16k,l). These findings indicated that the PyIm‐COF bearing pyridyl‐imine scaffolds effectively suppressed the two‐step 2e^¯^ ORR process and promoted the direct 2e^¯^ ORR pathway, leading to a higher yield of H_2_O_2_.

The robustness of the COFs is of paramount importance in photocatalysis as it ensures the structural integrity and catalytic activity of the COFs. However, most imine‐linked COFs have been observed to exhibit limited stability during photocatalysis, resulting in severe structural distortion, weak electron delocalization, and low charge separation. Several strategies have been employed to improve crystallinity, charge carrier transfer, and photostability of COFs for photosynthesis of H_2_O_2_.

##### a. Effect of Different Functional Groups

**Figure 17:**
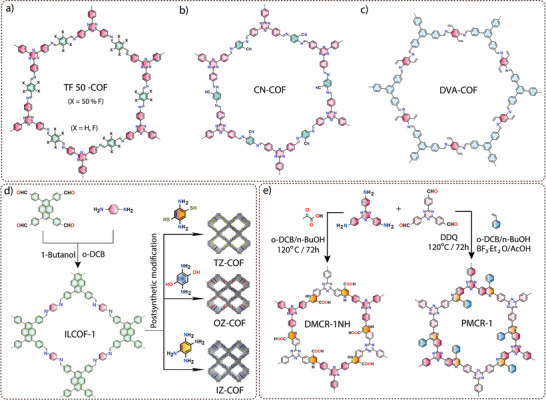
Influence of functionality in the COF backbone in enhancing the photocatalytic H_2_O_2_ generation. a) fluorinated‐COF^[^ ^151^ ^]^ b) Cyano‐containing COF^[^ ^152^ ^]^ and c) vinyl‐group anchored COF^[^ ^154^ ^]^ employed for photocatalytic H_2_O_2_ generation. d) Synthesis of azole‐based TZ‐COF, OZ‐COF and IZ‐COF via post‐synthetic modification. Reproduced with permission.^[^ ^156^ ^]^ Copyright 2023. Wiley‐VCH. e) Synthesis of DMCR‐1NH and PMCR‐1 COF via irreversible multicomponent one‐pot reaction. Reproduced with permission.^[^ ^159^ ^]^ Copyright 2023, American Chemical Society. Reproduced with permission.^[^ ^160^ ^]^ Copyright 2023. Wiley‐VCH.

To enhance the photocatalytic H_2_O_2_ generation performance, the functional groups present in the COF backbone play a crucial role. For example, Han and co‐workers incorporated an electron‐withdrawing group into the two‐dimensional imine‐linked triazine‐based COF by substituting fluorine (F) atoms on the peripheral aromatic unit of the terephthalaldehyde precursor.^[^ ^151^ ^]^ A series of three COFs such as H‐COF, TF‐COF, and TF_50_‐COF were prepared by reacting 4,4′,4″‐(1,3,5‐triazine‐2,4,6‐triyl)trianiline with terephthalaldehyde, 2,3,5,6‐tetrafluoroterephthalaldehyde, and an equimolar amount of both precursors, respectively. Owing to the combined result of structural and electronic properties, the partially fluorinated TF_50_‐COF (**Figure** **17**a) showed a relatively higher photocatalytic H_2_O_2_ rate of 1739 µmol g^−1^ h^−1^, compared to 1239 µmol g^−1^ h^−1^ for TF‐COF and 516 µmol g^−1^ h^−1^ for non‐fluorinated H‐COF. Furthermore, TF_50_‐COF was found to be selective and stable for photoreduction of O_2_ to H_2_O_2_ via a 2e^−^ oxygen reduction process, with an apparent quantum yield (AQY) of 5.1% and a solar‐to‐chemical conversion (SCC) efficiency of 0.17% at 400 nm, but with ethyl alcohol as the SED. Han and co‐workers also tried to improve the photocatalytic H_2_O_2_ generation performance by the decoration of the abundant cyano (‐C≡N) groups in the COF backbone, as the nitrile groups can induce the transfer and separation of photoinduced charges.^[^ ^152^ ^]^ The visible light absorption, charge separation, and transport efficiency were significantly improved and the photogenerated electrons were stabilized by introducing the –CN group on the 2D imine‐linked and triazine‐centred CN‐COF with a strong donor‐π‐acceptor structure (Figure 17b). Thereby, CN‐COF exhibited an enhanced intersystem crossing (ISC) efficiency for the selective formation of singlet oxygen (^1^O_2_), which facilitated the 2e^¯^ oxygen reduction process to form H_2_O_2_ with a high yield 2623 µmol h^−1^ g^−1^, which was significantly higher than without cyano‐containing H‐COF (1640 µmol h^−1^ g^−1^).

Later, Ni and co‐workers showed that the number of charge transfer channels between the electronic donor‐acceptor units of COFs can be controlled by varying the number of CN groups (*n* = 0, 1, 2) to biphenyl motifs.^[^ ^153^ ^]^ COF‐2CN with four charge transfer channels exhibited superior photocatalytic H_2_O_2_ generation compared to COF‐0CN with two charge transfer channels and COF‐1CN with three charge transfer channels. The vinyl group is also considered a suitable substituent to study the influence of the optoelectronic properties when decorated in the COF framework, as it is a non‐polar substituent with electron‐rich, π‐π conjugated structure and small volume, preserving the original accessibility of the internal pores of the material. Recently, Chen and co‐workers rationally designed a vinyl‐group anchored COF to investigate the effects of intralayer electron delocalization and interlayer π–π stacking on the photocatalytic performance for H_2_O_2_ generation (Figure 17c).^[^ ^154^ ^]^ The COF with vinyl groups (DVA‐COF) exhibited about tenfold higher H_2_O_2_ generation rate (845 µmol g^−1^ h^−1^), compared to the analogous PDA‐COF without vinyl functionalities.

##### b. Post‐Synthetic Modification in COFs

Thiophene is a well‐known electron‐rich heterocyclic molecule with intriguing electrical and optoelectronic properties. Taking advantage of the electron‐rich thiophene moiety, Van Der Voort and co‐workers synthesized an extended π‐conjugation system of imine‐linked COF (4PE‐N) from naphthalene‐2,6‐dicarbaldehyde (N) and 4,4′,4″,4‴‐(ethene‐1,1,2,2‐tetrayl)tetraaniline (4PE). The as synthesized imine‐linked 4PE‐N COF was further converted to the thiazole‐linked COF (4PE‐N‐S) via a simple post‐sulfurization process.^[^ ^155^ ^]^ In pure water, the photocatalytic H_2_O_2_ production rate of 4PE‐N‐S was found to be 1574 µmol g^−1^ h^−1^, which was approximately 5.8 higher than 4PE‐N. Considering the promising photocatalytic performance of the donor‐acceptor COFs, Wang and co‐workers demonstrated the generation of H_2_O_2_ by carefully tuning the microenvironment linkages in the COF frameworks bearing azoles with S, O or N heteroatoms.^[^ ^156^ ^]^ Azole linkages are often used as acceptors due to their electron‐deficient properties. In this work, pyrene‐based COFs, including thiazole‐linked TZ‐COF, oxazole‐linked OZ‐COF, and imidazole‐linked IZ‐COF, were synthesized from imine‐linked ILCOF‐1 by a post‐synthetic linker substitution strategy (Figure 17d). Thiazole‐linked TZ‐COF showed a comparatively higher H_2_O_2_ generation rate of 268 µmol h^−1^ g^−1^ than imidazole‐linked IZ‐COF (220 µmol h^−1^ g^−1^) and oxazole‐linked OZ‐COF (220 µmol h^−1^ g^−1^). The findings showed that the thiazole linkage resulted in a narrower bandgap, greater electron‐hole separation and stronger visible light absorption, leading to a preference for the formation of the ^•^O_2_^¯^ intermediate in the generation of H_2_O_2_ by the 2e^¯^ oxygen reduction process. To boost the photocatalytic activity of COFs for H_2_O_2_ generation, several synthetic strategies such as rapid sonochemical,^[^ ^157^ ^]^ liquid crystal (LC)‐directed,^[^ ^158^ ^]^ and irreversible one‐pot multicomponent reactions^[^ ^159^ ^]^ have also been employed. As example, we synthesized quinoline‐4‐carboxylic acid‐functionalized DMCR‐1NH COF^[^ ^159^ ^]^ and quinoline‐linked PMCR‐1 COF^[^ ^160^ ^]^ via irreversible multicomponent one‐pot Doebner reaction and Povarov reaction, respectively (Figure 17e). In pure water under air, H_2_O_2_ production was found to be 2264.5 µmol g^−1^ h^−1^ for DMCR‐1NH and it was shown that activity and photostability of the MCR‐COF in photocatalytic H_2_O_2_ production are superior to those of its imine‐based COF analogue. PMCR‐1 exhibited H_2_O_2_ generation rates of 1941 and 2265 µmol g^−1^ h^−1^ using ethanol and isopropyl alcohol as sacrificial agents, respectively, while an activity of 1294 µmol g^−1^ h^−1^ was observed in pure water, i.e., without addition of a sacrificial agent. Notably, 5500 µmol g^−1^ h^−1^ H_2_O_2_ was observed when benzyl alcohol was added, which was attributed to the strong π‐π interaction of aryl units of sacrificial agents and COF catalyst.

**Figure 18:**
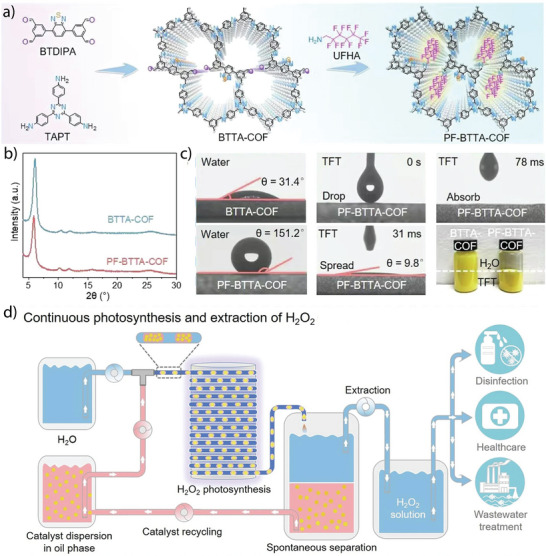
Continuous photosynthesis of H_2_O_2_ using a biphasic fluid system. a) Scheme of synthesis of the perfluoroalkyl‐modified PF‐BTTA‐COF. b) PXRD patterns of pristine BTTA‐COF and perfluoroalkyl‐modified PF‐BTTA‐COF. c) Water and oil (α,α,α‐trifluorotoluene, TFT) contact angle measurements of BTTA‐COF and PF BTTA‐COF, and the photograph of their dispersion in the H_2_O‐TFT biphasic system. d) Illustrative setup of a biphasic fluid system for continuous photosynthesis, separation, and extraction of H_2_O_2_. Reproduced with permission.^[^ ^161^ ^]^ Copyright 2024, Nature Springer.

Incorporating a superhydrophobic substrate into the COF backbone enhances H_2_O_2_ photosynthesis by improving water‐oxygen separation and minimizing recombination losses. This results in more efficient charge transfer, increased oxygen reduction reaction activity, and better overall catalytic performance in water‐splitting processes. Recently, Li and co‐workers incorporated a superhydrophobic substrate into the COFs via a post‐synthetic modification to explore a biphasic fluid system containing liquid water and oil phases − facilitating continuous photosynthesis, spontaneous separation and extraction of H_2_O_2_.^[^ ^161^ ^]^ An imine‐linked BTTA‐COF synthesized by solvothermal condensation reaction between 5,5′‐(benzo[c][1,2,5]thiadiazole‐4,7‐diyl)diisophthalaldehyde (BTDPA) and 2,4,6‐tris(4‐aminophenyl)‐1,3,5‐triazine (TAPT) was post‐synthetically grafted with perfluoroalkyl groups (1H,1H‐undecafluorohexylamine, UFHA) to obtain a superhydrophobic photocatalyst (PF‐BTTA‐COF) (**Figure** **18**a). The subsequent integration of perfluoroalkyl moieties into the COF did not disrupt the overall structural framework, retaining good crystallinity (Figure 18b). The surface hydrophobicity induced by the introduction of the perfluoroalkyl group was assessed using contact angle measurements (Figure 18c). The pristine BTTA‐COF exhibited a static water contact angle of 31.4°, while the perfluoroalkyl‐modified PF‐BTTA‐COF showed superhydrophobicity with a contact angle of 151.2°, allowing the COF material to be stably dispersed in the oil phase within a biphasic system of oil‐water. Taking this advantage, a biphasic flow photoreactor was demonstrated for continuous photosynthesis of H_2_O_2_ and extraction by generating a triple phase boundary, which improved O_2_ mass transfer (Figure 18d). As a result, a remarkable H_2_O_2_ generation rate of up to 968 µmol h^−1^ was achieved with tunable concentrations from 2.2 to 38.1 mM. The biphasic fluid system could be promising for the direct use of the produced H_2_O_2_ in practical applications, especially medical disinfectant and wastewater treatment.

##### c. Impact of the Polar Groups in the COF Backbone

**Figure 19:**
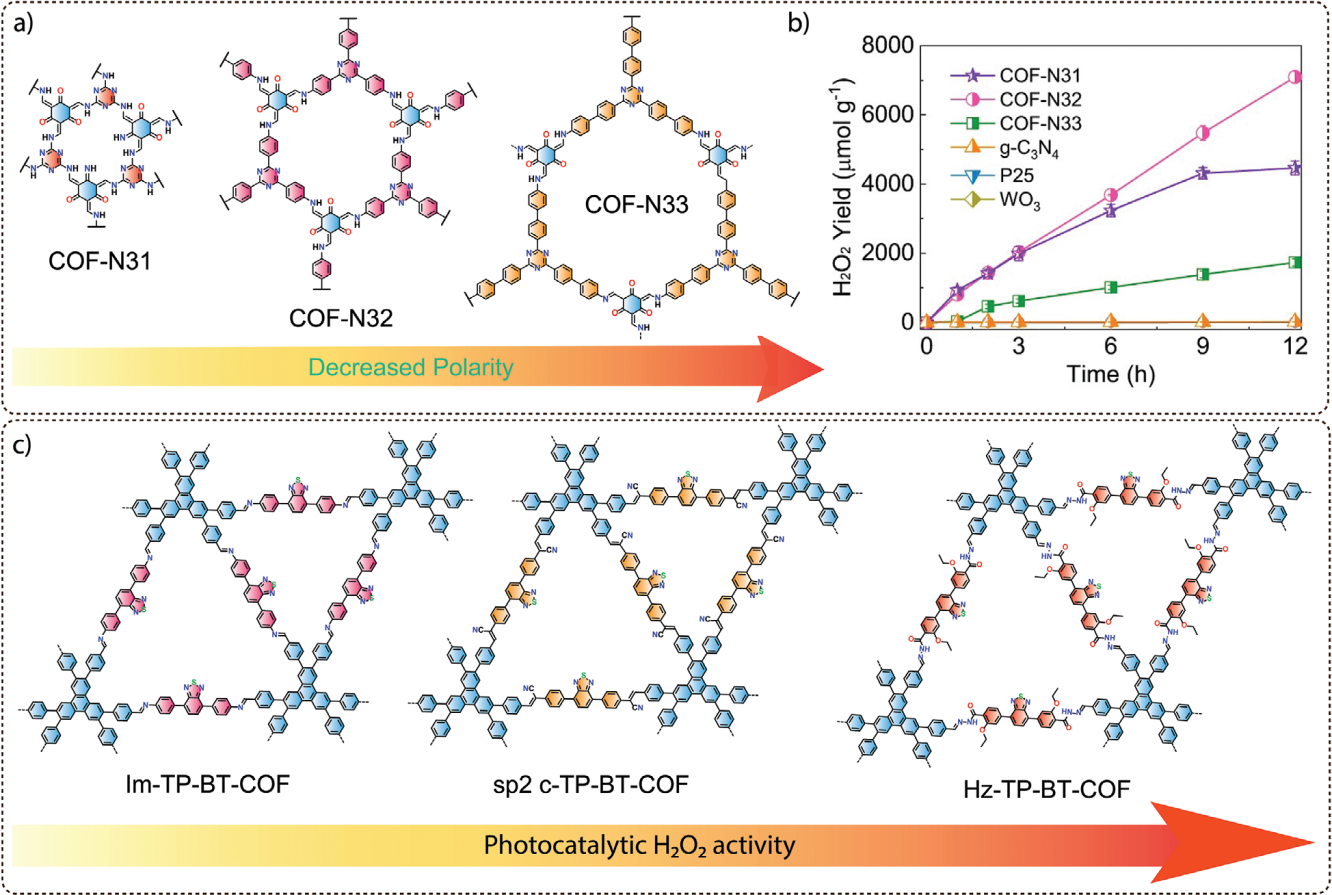
Influence of intramolecular polarity and linkages of COFs in photocatalytic H_2_O_2_ generation. a) Synthesis of donor‐acceptor COF‐N31, COF‐N32, and COF‐N33 COFs with different intramolecular polarity, and b) their photocatalytic activity for H_2_O_2_ generation. Reproduced with permission.^[^ ^46^ ^]^ Copyright 2023, Springer Nature. c) Imine‐linked Im‐TP‐BT‐COF, vinylene‐linked sp^2^c‐TP‐BT‐COF, and hydrazine‐linked Hz‐TP‐BT‐COF are used for H_2_O_2_ generation from pure water.^[^ ^162^ ^]^

The intramolecular polarity of organic building blocks has a significant role in water splitting, particularly in the water oxidation process. The critical role of triazine *N*‐sites with optimal N*2p* states in the selective oxidation of H_2_O into H_2_O_2_ was demonstrated by N, and coworkers.^[^ ^46^ ^]^ To evaluate the effect of intramolecular polarity, a set of three donor‐acceptor COFs such as COF‐N31, COF‐N32 and COF‐N33 were synthesized from triazine‐cored triamines: 1,3,5‐triazine‐2,4,6‐triamine, 4,4′,4′“‐(1,3,5‐triazine‐2,4,6‐triyl)‐tri, aniline and 4,4′,4′”‐(1,3,5‐triazine‐2,4,6‐triyl)tris(([1,1′‐biphenyl]‐4‐amine)), respectively (**Figure** **19**a). Photocatalytic H_2_O_2_ rates of 442 µmol g^−1^ h^−1^, 605 µmol g^−1^ h^−1^ and 155 µmol g^−1^ h^−1^ were observed with COF‐N31, COF‐N32 and COF‐N33, respectively without using sacrificial agent (Figure 19b). This study suggests that COF‐N32 with a moderate intramolecular polarity showed greater photocatalytic performance compared to COF‐N31, with a stronger or COF‐N33 with a weaker intramolecular polarity. The strong intramolecular polarity of COF‐N31 hindered π‐conjugation and promoted rapid exciton dissociation, resulting in reduced photocatalytic activity to generation H_2_O_2_. The weak polarity in COF‐N33, on the other hand, helps the π‐conjugation effect in some way.

Recently, Jiang and co‐workers have demonstrated the effect of the linkages involving COFs on photocatalytic H_2_O_2_ generation.^[^ ^162^ ^]^ To evaluate the non‐conjugated hydrazone linked Hz‐TP‐BT‐COF, partially π‐conjugated imine linked Im‐TP‐BT‐COF and the fully π‐conjugated vinylene linked sp^2^c‐TP‐BT‐COF were used for H_2_O_2_ generation from pure water (Figure 19c). To achieve efficient carrier generation and ample catalytic sites, electron‐rich hexaphenyl‐substituted triphenylene as donor and electron‐deficient benzothiadiazole as acceptor units were incorporated into the COF backbone. Owing to the dense donor‐acceptor lattices sites for water oxidation and oxygen reduction, hydrazone‐linked Hz‐TP‐BT‐COF exhibited a maximum H_2_O_2_ generation rate of 5.7 mmol g^−1^ h^−1^, compared to the imine‐linked Im‐TP‐BT‐COF (0.6 mmol g^−1^ h^−1^) and vinylene‐linked sp^2^c‐TP‐BT‐COF (2.3 mmol g^−1^ h^−1^). Moreover, Hz‐TP‐BT‐COF showed a high apparent quantum yield (AQY) of 17.5% and a turnover frequency of 4.2 h^−1^ at 420 nm without the use of metals or sacrificial donors. This study provides an idea for the design of novel COFs with a reticulated π‐architecture, which can exhibit efficient transport and harvesting of photogenerated charge and well‐defined hydrophilic docking sites for water and oxygen, leading to accelerated water oxidation and oxygen reduction process to efficiently generate H_2_O_2_.

#### 4.4.2 Covalent Triazine Frameworks as Photocatalysts

**Figure 20:**
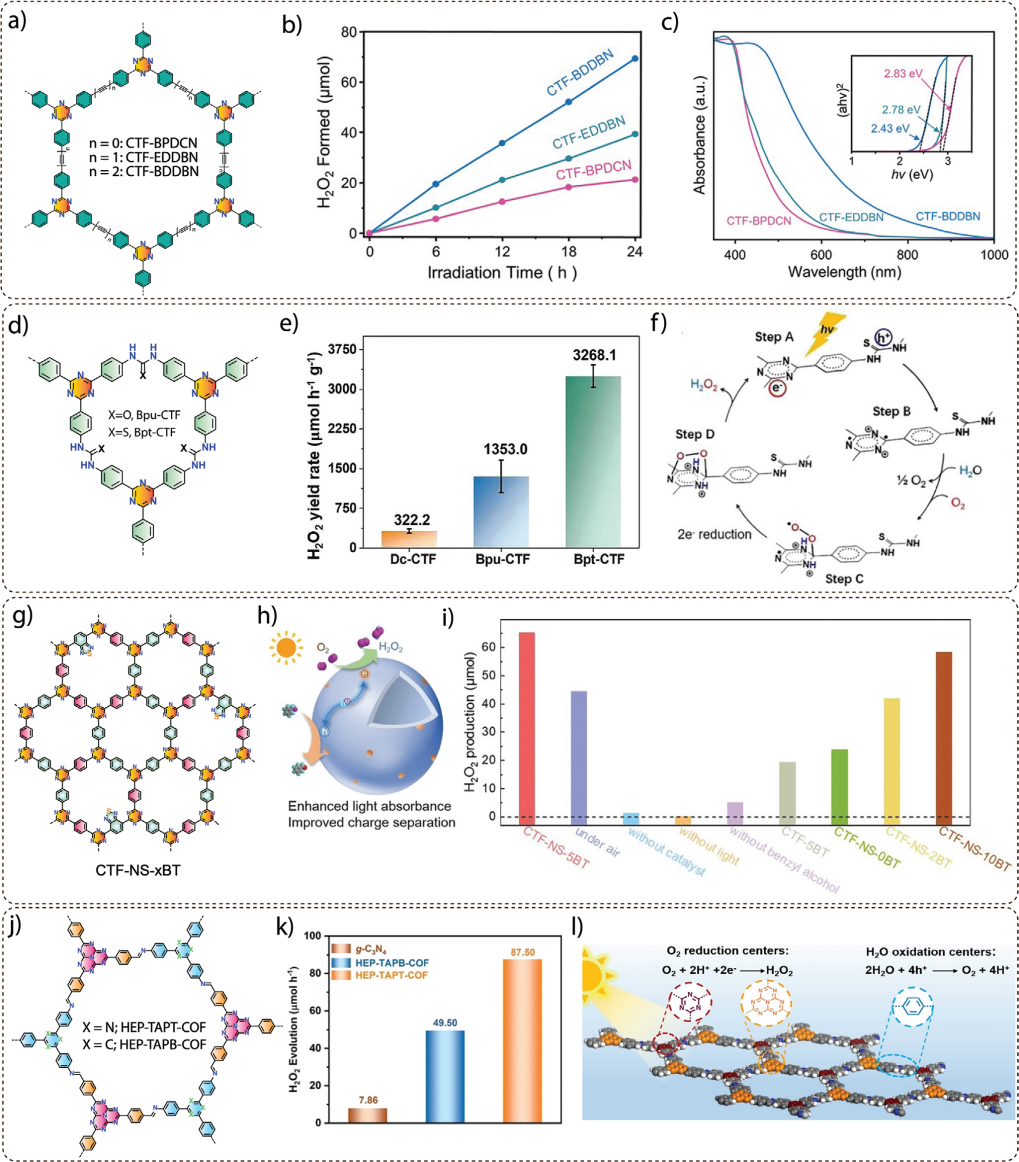
Synthesis and applications of CTFs for photocatalytic H_2_O_2_ generation. a) The general framework of CTFs containing acetylene and diacetylene units, namely CTF‐EDDBN, CTF‐BDDBN, and CTF‐BPDCN. b) Photocatalytic H_2_O_2_ generation using CTF‐EDDBN, CTF‐BDDBN and CTF‐BPDCN. c) UV‐Vis spectra with the corresponding Tauc plots of exfoliated CTFs. Reproduced with permission.^[^ ^163^ ^]^ Copyright 2020. Wiley‐VCH d,e) Rational design of (thio)urea‐functionalized CTFs (Bpt‐CTF and Bpu‐CTF) for photocatalytic H_2_O_2_ generation. f) Mechanism for the photocatalytic generation of H_2_O_2_ on Bpt‐CTF. Reproduced with permission.^[^ ^164^ ^]^ Copyright 2022. Wiley‐VCH g,h) Functionalized CTF‐NS‐xBT nanoshells and the photosynthesis reaction mechanism for H_2_O_2_ production. i) Photocatalytic H_2_O_2_ generation by CTF‐NS nanoshells under different conditions. Reproduced with permission.^[^ ^165^ ^]^ Copyright 2021. Wiley‐VCH. (j–l) Crystalline s‐heptazine‐based COFs, such as HEP‐TAPT‐COF, HEP‐TAPB‐COF, and their photocatalytic performances for H_2_O_2_ generation via oxygen reduction and water oxidation. Reproduced with permission.^[^ ^166^ ^]^ Copyright 2023. Wiley‐VCH.

Owing to the extended conjugated structure in two dimensions, high nitrogen content, thermal/chemical stability and light absorption in the visible region, the potential application of covalent triazine frameworks (CTFs) for photocatalytic hydrogen peroxide production has also been explored. Xu and co‐workers developed unique CTF‐based photocatalysts bearing acetylene and diacetylene units, namely, CTF‐EDDBN and CTF‐BDDBN for producing H_2_O_2_ (**Figure** **20**a).^[^ ^163^ ^]^ This study suggested that the introduction of acetylene or diacetylene units into CTF backbones improved the photocatalytic H_2_O_2_ production with the rate of 39 and 70 µmol g^−1^ h^−1^ for CTF‐EDDBN and CTF‐BDDBN, respectively, as compared to that of CTF‐BPDCN without bearing acetylene and diacetylene moieties (Figure 20b). This improvement comes from the carbon‐carbon triple bonds that can enhance the coplanar conformation in CTFs, which promotes charge separations in the conjugated structures. Additionally, CTF‐BDDBN nanosheets showed a broader light absorption in the visible region with a narrow band gap of 2.43 eV (Figure 20c). Nevertheless, the photocatalytic performances of CTF‐BDDBN toward H_2_O_2_ production were found to be very low due to the fast exciton recombination, which could be caused by the amorphous nature of the CTF‐based photocatalysts.

Further, Han and co‐workers designed (thio)urea‐functionalized CTFs (symbolized as Bpt‐CTF and Bpu‐CTF), and successfully prepared them via trimerization reactions of 1,3‐bis(4‐cyanophenyl) thiourea (Bpt) and 1,3‐bis(4‐cyanophenyl)urea (Bpu) precursors, respectively (Figure 20d).^[^ ^164^ ^]^ The thiourea‐functionalized Bpt‐CTF showed a remarkable enhancement in the photocatalytic H_2_O_2_ production via 2e^‐^ ORR pathway with a rate up to 3268 µmol h^−1^ g^−1^, and an excellent apparent quantum yield of 8.6% without using any sacrificial agent or cocatalyst (Figure 20e‐f). The photocatalytic H_2_O_2_ generation rate of Bpt‐CTF was significantly greater than unfunctionalized Dc‐CTF (322.2 µmol h^−1^ g^−1^) and urea‐functionalized Bpu‐CTF (1353 µmol h^−1^ g^−1^). To tune the optoelectronic properties and improve the photocatalytic performances, Li and co‐workers fabricated CTF nanoshells via a co‐monomer doping method by incorporating electron‐deficient benzothiadiazole (BT) units into the conjugated networks of CTFs (Figure 20g).^[^ ^165^ ^]^ The CTF‐NS‐5BT (with BT content of 5 mol%) showed a substantial H_2_O_2_ generation rate of 1630 µmol g^−1^ h^−1^ in the aqueous O_2_‐saturated solution under visible light irradiation. The H_2_O_2_ production rate of CTF‐NS‐5BT was about 3‐3.3 folds higher than that of CTF‐NS‐0BT and nonhollow CTF‐5BT, respectively (Figure 20h‐i). The photocatalytic H_2_O_2_ generation activities of CTF‐NS‐BT with nanoshell morphology were subsequently enhanced due to the incorporation of strong electron‐withdrawing BT units in the CTFs, improving visible‐light absorption and electronic delocalization along the frameworks. Nevertheless, all these CTF‐based photocatalysts were amorphous in nature without long‐range ordered structures.

Recently, Chen and co‐workers demonstrated that crystalline *s*‐heptazine‐based COFs with separated redox centers (HEP‐TAPT‐COF and HEP‐TAPB‐COF) can generate H_2_O_2_ from oxygen and pure water via the solar‐driven direct 2e^−^ reaction pathway without generating reactive radicals (Figure 20j).^[^ ^166^ ^]^ HEP‐TAPT‐COF and HEP‐TAPB‐COF were synthesized from *s*‐heptazine based precursor (HEP‐OAc) and 1,3,5‐tris(4‐aminophenyl)triazine (TAPT) or 1,3,5‐tris(4‐aminophenyl)benzene (TAPB), respectively via a solvothermal condensation reaction. HEP‐TAPT‐COF with triazine core showed a maximum H_2_O_2_ generation rate of 1750 µmol h^−1^ g^−1^, more significant than that of HEP‐TAPB‐COF with phenyl unit (990 µmol h^−1^ g^−1^) and *g*‐C_3_N_4_ (Figure 20k). The superior photocatalytic H_2_O_2_ efficiency of HEP‐TAPT‐COF was attributed to integrating dual oxygen reduction active centers of *s*‐heptazine and triazine units in the HEP‐TAPT‐COF framework (Figure 20l). Therefore, considering the high photocatalytic H_2_O_2_ performances of *s*‐heptazine‐based COFs, it can be concluded that the development of high nitrogen‐containing crystalline COFs bearing s‐heptazine and triazine moieties is highly sought after, which could be useful for the efficient photocatalytic production of H_2_O_2_ from water under solar light in the near future.

#### 4.4.3 Metallated COFs as Photocatalysts

The H_2_O_2_ generation activity of hybrid COF‐based materials has also been investigated by incorporating various conductive or photoactive metal precursors into the COF framework. Gu and co‐workers demonstrated that a three‐dimensional titanium‐based COF(TiCOF‐spn) with *spn* topology could generate H_2_O_2_ up to 489 µmol g^−1^ h^−1^ due to the combined properties of crystalline porous framework, triazine unit and photoactive titanium‐metal core.^[^ ^167^ ^]^ Similarly, Jiang and coworkers studied the photocatalytic activity of two‐dimensional crystalline piperazine‐linked hybrid COFs, namely, CoPc‐DAB‐COF and CoPc‐BTM‐COF, which exhibited a reasonably good H_2_O_2_ generation rate of 1851 and 2096  µmol h^−1^ g^−1^, respectively.^[^ ^168^ ^]^ Metal‐isolated clusters (MICs) often suffer from poor stability due to increased surface free energy during the catalytic reaction.^[^ ^169^ ^]^ To stabilize metal‐isolated clusters as photocatalysts, Guo and co‐workers demonstrated a new strategy of fluorinated COFs to strongly confine the Pd metal‐isolated clusters through strong metal‐support interaction to significantly enhance the photocatalytic stability and activity in the H_2_O_2_ generation process.^[^ ^170^ ^]^ The fluorinated TAPT‐TFPA COFs@Pd ICs showed an excellent H_2_O_2_ generation rate of 2143 µmol h^−1^ g^−1^ with high photocatalytic stability over 100 h, which was about 1.9 times greater than that of TAPT‐PBA COFs@Pd ICs with the same Pd content (1140 µmol h^−1^ g^−1^). The improved activity and photostability of TAPT‐TFPA COFs@Pd ICs were attributed to the introduction of strong electronegative fluorine atoms into the nanosized confinement region of the COF skeleton and the enhancement of the metal‐support interaction between Pd ICs and COFs, resulting in the lowering of the *d*‐band center (ε_d_).

### 4.5 Nitrogen (N_2_) Reduction

Ammonia (NH_3_) is an essential compound for life on earth and serves as a basic building block for many applications as it is widely used in fertilizers, including urea, ammonium nitrate, ammonium sulfate, and ammonium bicarbonate.^[^ ^171^, ^172^ ^]^ In addition, ammonia holds great promise as a clean energy source. Naturally, ammonia is synthesized by nitrogen fixation − a process facilitated by the enzyme nitrogenase found in certain bacteria. The first industrial production of ammonia from nitrogen took place in 1913 using the Haber‐Bosch (HB) process, which involves the reaction of hydrogen and nitrogen at high temperatures (573‐773 K) and pressures (100‐200 atm) and is responsible for ≈1.5% of annual global CO_2_ emissions.^[^ ^173^, ^174^ ^]^ Therefore, the development of more economical and environmentally friendly nitrogen reduction methods has long been a major challenge for chemists. In this context, photocatalytic nitrogen reduction reaction (NRR) shows significant potential for the production of ammonia using clean and inexhaustible sunlight. Despite extensive research on traditional photocatalysts such as metal oxides, sulfides, bismuth oxyhalides, and *g*‐C_3_N_4_ for nitrogen fixation, these materials face significant challenges in achieving efficient ammonia NH_3_ production. COFs have also rarely been used as photocatalysts for nitrogen reduction due to the high activation energy required for nitrogen reduction.

**Figure 21:**
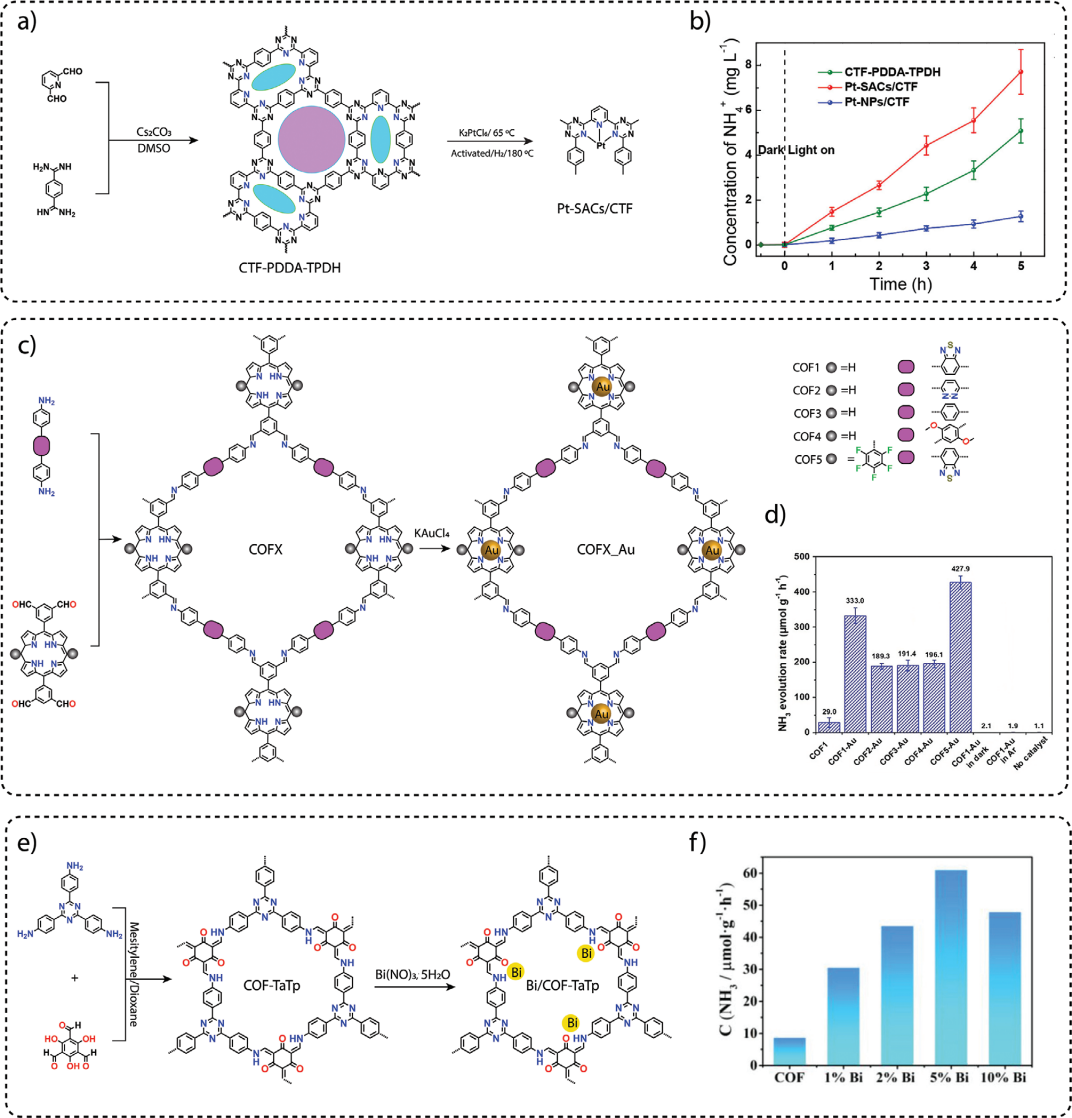
a) Synthesis of Pt‐SACs/CTF using CT‐PDDA‐TPDH. b) NH_4_^+^ production over time using CT‐PDDA‐TPDH, Pt‐SACs/CTF, and Pt‐NPs/CTF. Reproduced with permission.^[^ ^175^ ^]^ Copyright 2020, American Chemical Society. c) Synthesis of Au incorporated COFX_Au COF. d) NH_3_ evolution rate for various Au‐based COFX. Reproduced with permission.^[^ ^176^ ^]^ Copyright 2023, American Chemical Society. e) Synthesis of Bi incorporated Bi/COF‐TaTp. f) The performance of NH_3_ production is varying concentrations of Bi‐incorporated COF. Reproduced with permission.^[^ ^177^ ^]^ Copyright 2023, Wiley‐VCH.

To address this challenge, the synthesis of a single‐atom platinum (Pt) catalyst anchored to the N_3_ sites of stable and ultrathin CTF nanosheets (Pt‐SACs/CTF) was demonstrated (**Figure** **21**a).^[^ ^175^ ^]^ To explore photocatalytic N_2_ fixation, experiments were performed in N_2_‐saturated water under visible light (420 nm < λ < 780 nm) without any sacrificial agent, which showed a low ammonia production rate of 171.40 µmol g^−1^ h^−1^ (Figure 21b). Zhao and co‐workers synthesized a series of isostructural Au‐immobilised porphyrin‐based COFs (COFX‐ Au, X = 1–5) with different functional groups at the distal (benzothiadiazole, pyrazine, benzene or dimethoxybenzene) and proximal (hydrogen or pentafluorobenzene) positions of the porphyrin units (Figure 21c).^[^ ^176^ ^]^ In these COFs, the photogenerated electrons are transported to the Au sites, facilitating the reduction of nitrogen to produce ammonia. Notably, upon light irradiation, COF1‐Au and COF5‐Au decorated with strong electron‐withdrawing groups showed higher ammonia production rates of 333.0 ± 22.4 and 427.9 ± 18.7 µmol g^−1^ h^−1^, respectively, outperforming COF2‐Au, COF3‐Au, and COF4‐Au (Figure 21d). In contrast, metal‐free COF1 exhibited poor activity with an ammonia evolution rate of 29 ± 13.6 µmol g^−1^ h^−1^, highlighting the importance of Au as the active catalyst center.

The main challenges associated with the activation of nitrogen are the simultaneous water oxidation and competitive hydrogen evolution reactions. To address this challenge, suppression of the hydrogen evolution reaction has been demonstrated to significantly enhance photocatalytic ammonia production using COF as a photocatalyst. The synthesis of Bi/COF‐TaTp was achieved by immobilizing bismuth (Bi) in the imine‐linked COF‐TaTp through N‐Bi‐O coordination (Figure 21e).^[^ ^177^ ^]^ The incorporation of 5% bismuth into COF‐TaTp resulted in a significant enhancement of the photocatalytic ammonia production, reaching a rate of 61 µmol g^−1^ h^−1^, which was 4.7 times higher than that of the pristine COF‐TaTp (Figure 21f). DFT calculations revealed that the integrated bismuth species significantly enhanced carrier transfer and nitrogen activation via a donation‐back‐donation mechanism. At the same time, bismuth effectively suppressed the hydrogen evolution reaction, as evidenced by a higher hydrogen binding free energy (ΔGH*) of 2.62 eV for Bi/COF‐TaTp compared to 2.31 eV for the pristine COF‐TaTp.

### 4.6 Co‐enzyme Regeneration

Photocatalytic coenzyme regeneration is an important task because it enables the continuous use of enzymes in various biochemical processes in a more sustainable and cost‐effective manner. Enzymes often lose activity after catalyzing reactions and photocatalytic enzyme regeneration reduces the need for frequent replacement and maintains enzyme activity for more extended periods of time, improving the efficiency of industrial processes, including biofuel production, pharmaceutical synthesis, and environmental remediation. It also supports green chemistry initiatives by minimizing waste and energy consumption. Although semiconductor materials such as TiO_2_, MOFs and carbon‐based materials such as graphene have been used for photocatalytic coenzyme regeneration, poor stability under reaction conditions, limited light absorption range and potential enzyme deactivation due to photocatalyst‐enzyme interactions are the main limitations for their use.

**Figure 22:**
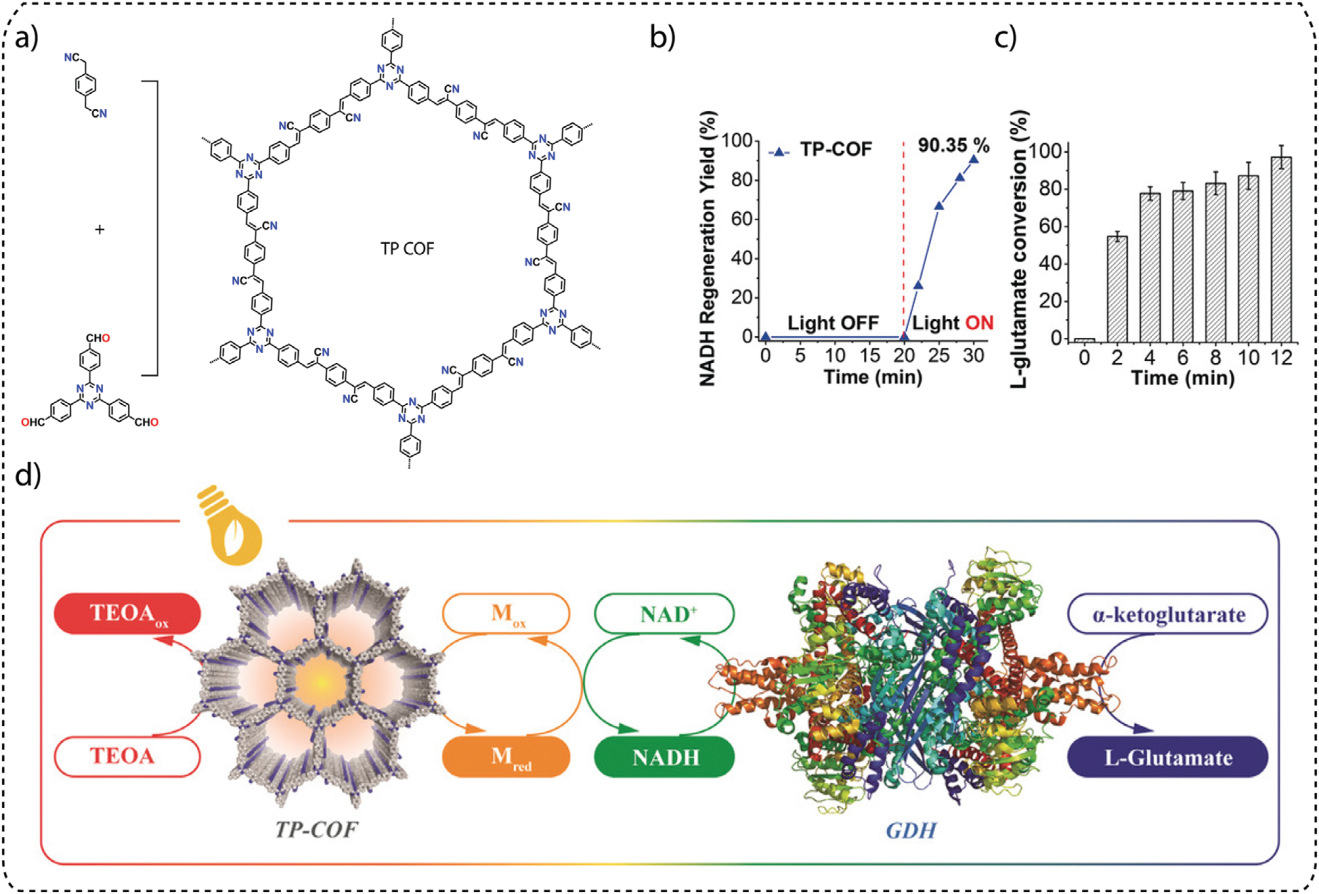
a) Synthesis of cyanovinylene‐linked TP COF. b) Photocatalytic NADH regeneration in the presence of light and dark conditions. c) L‐glutamate formation over time using NADH. d) Schematic representation of the artificial PSI‐induced regeneration of the coenzyme and the photoenzymatic synthesis of the l‐glutamate by l‐glutamate dehydrogenase (GDH). Reproduced with permission.^[^ ^178^ ^]^ Copyright 2019, Wiley‐VCH.

The nature of biocatalytic processes requires precise control of the reaction environment, which is also a challenge for the application of COFs in this field. Recently, considering the robustness of fully sp^2^‐carbon conjugated systems, Zhao and co‐workers explored a two‐dimensional cyanovinylene‐linked TP‐COF as an artificial photosystem I for NADH coenzyme regeneration (**Figure** **22**a).^[^ ^178^ ^]^ The NADH regeneration efficiency using TP‐COF was found to be 90.4% within 10 minutes in the presence of TEOA as a sacrificial electron donor at a concentration of 1 mg/mL of TP‐COF catalyst (Figure 22b). The resulting TP‐COF showed a significantly improved coenzyme regeneration efficiency for the conversion of *α*‐ketoglutarate to *L*‐glutamate, up to 97% yield within 12 minutes (Figure 22c). The excellent photocatalytic activity could be attributed to the highly ordered π‐conjugated framework and the suitable band gap and energy level of TP‐COF, indicating a valuable potential as an excellent artificial photosystem I for both coenzyme regeneration and various coenzyme‐assisted redox enzyme reactions for complex compound synthesis (Figure 22d).

### 4.7 Photocatalytic Uranium Extraction

**Figure 23:**
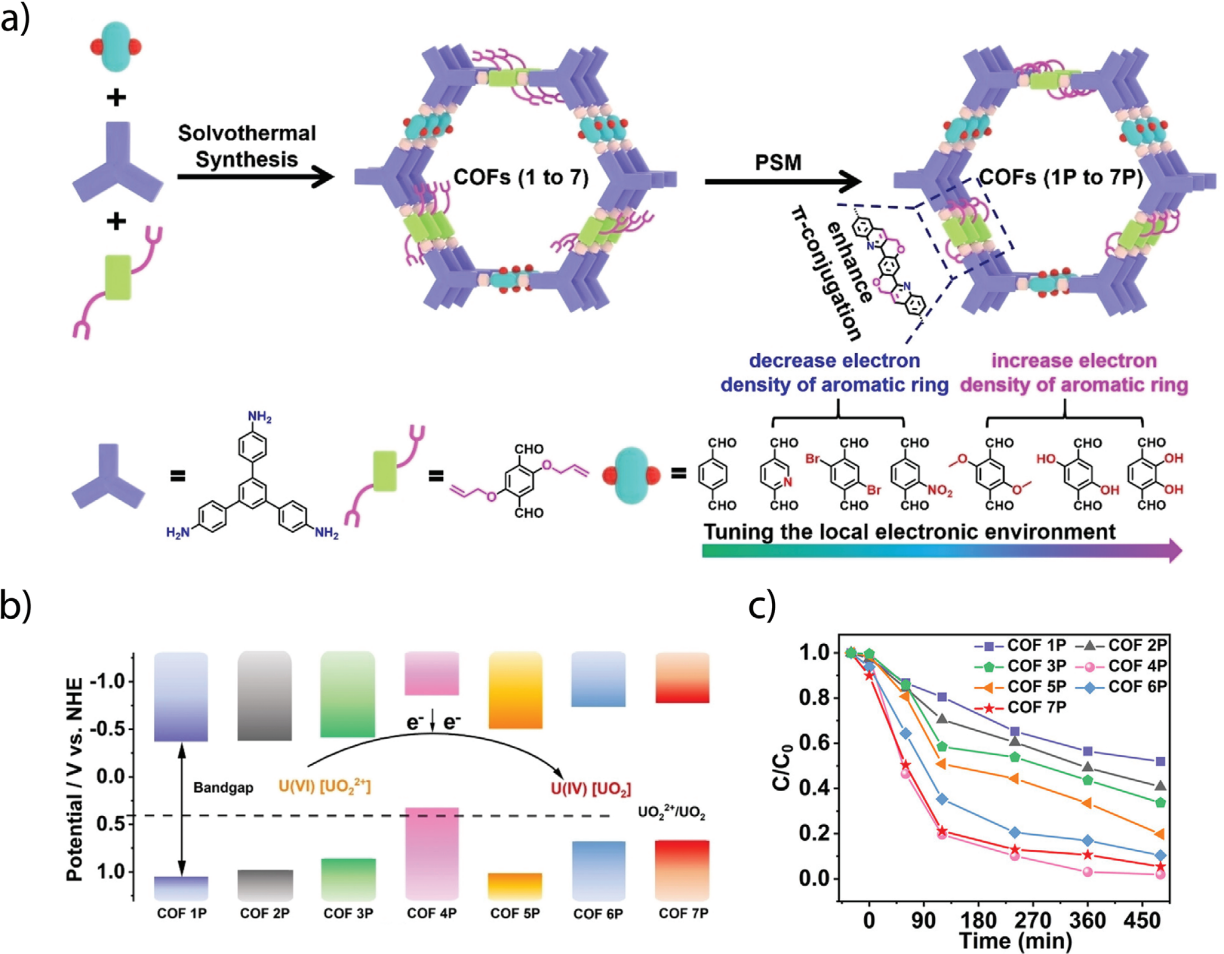
a) Scheme of synthesis of pristine COFs (1 to 7) and their post‐synthetically modified COF counterparts (1P to 7P). b) Bandgap and band position of post‐synthetic modified COFs (1P to 7P). c) Uranium extraction efficiency of post‐synthetic modified COFs (1P to 7P). Reproduced with permission.^[^ ^179^ ^]^ Copyright 2023. Wiley‐VCH.

Photocatalytic uranium extraction offers an energy‐efficient and environmentally friendly method for recovering uranium from seawater or contaminated water sources. It involves the photocatalytic reduction of U(VI) to U(IV) to enhance uranium adsorption in COFs, because U(IV) forms stronger and more stable coordination complexes with possible functional moieties within the COF, thereby improving their binding affinity for uranium in seawater. Photocatalytic uranium extraction therefore uses sunlight to drive the extraction process, minimizing chemical waste, while selective photocatalysts increase the efficiency and specificity of uranium capture, making it a promising approach for sustainable uranium recovery. In recent developments, Ma and co‐workers investigated donor‐acceptor COFs by modifying the local charge distribution to achieve light‐assisted uranium extraction. In these regards, isoreticular COFs were synthesized, designated as COF 1 to COF 7, via the imine condensation of 1,3,5‐tris(4‐aminophenyl)benzene, 2,5‐bis(allyloxy)terephthalaldehyde, and various 1,4‐phthalaldehyde analogues. Following this, a post‐synthetic conversion of the synthesized imine COFs to chromenoquinoline‐based COFs, referred to as COFs 1P to 7P, was accomplished through Povarov reactions under solvothermal conditions (**Figure** **23**a).^[^ ^179^ ^]^

The photocatalytic uranium extraction experiments using seawater showed that several post‐synthetically modified COFs (COF 4P, COF 6P, and COF 7P) exhibit notable efficiency as photocatalysts for uranium extraction without the need for sacrificial reagents. In particular, COF 4P achieved rapid kinetics with a uranium uptake efficiency of 8.02 mg g^−1^ d^−1^ in natural seawater (Figure 23b). Further experimental and theoretical observations revealed that the electron‐withdrawing groups (in the order of decreasing ability: nitro > bromine > pyridine N) and electron‐donating groups (in the order of increasing ability: o‐dihydroxy ≈ p‐dihydroxy > methoxyl) anchored on the aromatic rings of the linkers in the COFs resulted in a non‐uniform distribution of electron density. This configuration effectively enhanced the intramolecular interactions between electron donors and acceptors within the pores, thereby improving the optoelectronic properties of the COFs and facilitating charge carrier separation (Figure 23c). In a similar study, the quaternary COF, COF 2‐Ru‐AO, exhibited a significant uranium uptake capacity of 2.45 mg g^−1^ day^−1^ in seawater.^[^ ^180^ ^]^ However, the mechanisms and photogenerated products associated with these reactions have not been thoroughly investigated. Therefore, these topics are not discussed in detail in this review.

COFs are also known for several other photocatalytic applications such as dye degradation and reactive oxygen species (ROS) generation.^[^ ^181^, ^182^, ^183^, ^184^, ^185^ ^]^ However, the exact mechanisms and photogenerated products are not well understood as these reactions often involve complex pathways rather than direct electron transfer. These reactions can lead to a variety of intermediate products, making it difficult to pinpoint a clear mechanistic pathway. Additionally, dye degradation is more of a photochemical process rather than true photocatalysis, as it doesn't necessarily involve the selective conversion of substrates into value‐added products, which is a key characteristic of photocatalysis. Hence, we have not discussed dye degradation much in this review.

## 5 Conclusions and Perspectives

In addition to their role in gas storage and separation, environmental remediation, energy storage, and sensing, COFs are attracting considerable attention in heterogeneous photocatalysis due to their exceptional properties such as high crystallinity, persistent porosity, π‐conjugated systems, tuneable band structures, and adaptable molecular architectures.^[^ ^186^, ^187^, ^188^, ^189^ ^]^ The well‐ordered π‐conjugated structures of COFs facilitate efficient light absorption, enhancing charge carrier separation and transport while reducing hole‐electron recombination. In addition, the tuneable molecular structure allows precise tailoring of active sites and optoelectronic properties, enhancing interactions with substrates or co‐catalysts and promoting surface reactions.

Due to these advantages, COFs have been extensively investigated over the last decade for various photocatalytic applications, including hydrogen evolution via water splitting, hydrogen peroxide generation, carbon dioxide reduction, nitrogen reduction, and organic transformations. This review summarizes the progress made in COF‐based photocatalysis, focusing on their intrinsic properties, the basic principles of photocatalytic hydrogen production, hydrogen peroxide generation, organic transformations, and carbon dioxide and nitrogen reduction. In addition, potential strategies to enhance the photocatalytic performance of COFs are highlighted, including methods for COF synthesis, molecular structure regulation, linkage tailoring, and cocatalyst loading.

Despite the considerable progress achieved with COF photocatalysts, these materials are still in the early stages of development, particularly regarding commercial and industrial applications. The following section addresses the key issues, current challenges, and future prospects of COFs in photocatalysis:

i)
**Toward real‐life application** **s**: At present, photocatalytic hydrogen evolution using COF is limited by its low generation rate and poor apparent quantum yield in the visible light region. Considering the quantities of products such as hydrogen, ethylene, or ammonia that our economy requires, hundreds of millions of tonnes annually, the reported conversion rates of photocatalysis to date seem too low to achieve commercial or industrial feasibility. However, very many smaller, decentralized, and easier‐to‐handle photocatalytic production units could be a future way to meet our demands, especially when new political situations or economic factors increase the pressure to identify alternative procurement routes for these raw materials. This situation has actually already materialized, and the need to establish new sustainable production methods for chemicals and fuels will certainly increase rather than decrease in the future. Besides these commodity chemicals/fuels, it is highly probably that photocatalytic production might be the first commercially available for niche applications, e.g. the production of chemicals in remote areas, such as H_2_O_2_ production for disinfection/medical purposes.

**Figure 24:**
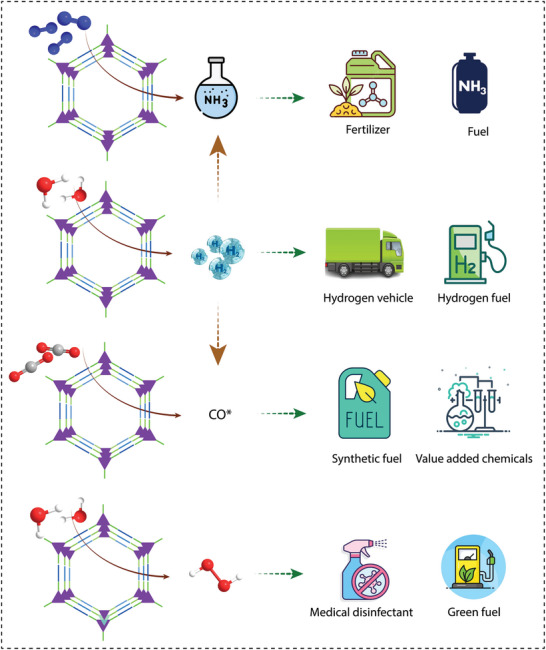
Direct use of the photogenerated products using COF‐based materials for commercially essential reactions.

Conversion rates can probably be solved by further improving the structure of COFs for effective charge carrier generation, efficient electron‐hole separation, and migration. However, another challenge is that most of the current reports on photocatalytic hydrogen evolution, CO_2_ reduction, and H_2_O_2_ generation require sacrificial reagents. Therefore, water oxidation as a second half‐reaction must become much more of a focus of research. To avoid the use of sacrificial reagents in photocatalytic reactions, spatially controlled deposition of cocatalyst combinations on single component photocatalysts or heterojunction and Z‐scheme photocatalytic systems, respectively, should be given greater attention. In addition, the selectivity in CO_2_ reduction and organic conversion to the target single product is still unsatisfactory. Finally, most COF photocatalysts reported needing expensive metal cocatalysts. Therefore, there is an urgent need to develop noble metal‐free COF‐based photocatalysts or to explore low‐cost cocatalysts derived from earth‐abundant materials.

ii)
**Improvement of photochemical stability**: An essential requirement for the reuse of COFs in practical photocatalytic reactions is their high photochemical stability. In recent years, considerable effort has been devoted to the diversity of COFs, including building blocks, linkages, synthetic methods and photocatalytic functionalities. Although few sets of COFs are quite stable, there is still room for further improvement. Further research to improve the stability of COFs is highly desirable to ensure long‐term stability under harsh conditions.
iii)
**Estimation of mechanistic studies**: It is worth noting that the detailed mechanisms for the photocatalytic reactions are still unclear due to the complexity and heterogeneous nature of the photocatalytic process in COFs. Therefore, further studies and deeper insights should be initiated by monitoring the product formation process and studying the theoretical calculations to predict the intermediates during COF photocatalysis, which can provide direct evidence for the photocatalytic mechanisms.
iv)
**Large‐scale synthesis of COFs**: Despite ongoing efforts to synthesize COFs, conventional techniques such as solvothermal, ionothermal, and mechanochemical grinding have remained limited to laboratory‐scale synthesis. Thus, for the synthesis of crystalline and porous COFs on a large scale, potential efforts and strategies are required to optimize kinetic and thermodynamic parameters. For the synthesis of COFs on an industrial scale, continuous flow or flux synthesis without or minimal solvent addition may be useful, extending their applications from the laboratory to industry.^[^ ^190^, ^191^, ^192^ ^]^ In an effort to prepare the COFs in bulk scale, Van dDer Voort and co‐workers have recently shown that a wide variety of imine‐linked COFs with high crystallinity can be synthesized in a single batch of over ten grams using green and inexpensive aqueous alcohol solutions (e.g., *n*‐butanol) as solvents and acetic acid as a catalyst at 70 °C for 16 h — demonstrating the possibilities of COF synthesis on a large scale.^[^ ^193^ ^]^
v)
**Processing and shaping of COF photocatalysts**: Most photocatalytic experiments and tests on a laboratory scale are performed using dispersions of powder photocatalysts. While this is quite practical on a lab scale, it is hardly applicable on larger scales, where rather films, membranes, granules, or other morphologies of photocatalysts would be applied. While most solvothermal synthesis methods yield COFs as non‐processable powders, recent years have seen the development of new synthetic techniques to process COFs in various shapes. In this regard, it would be certainly useful to investigate not only the influence of the chemical structure and topology of COFs on their photocatalytic performance but also their macrostructure and morphology.
vi)
**Direct use of the produced products**: Finally, the in‐situ use of photogenerated products can be a significant advantage, avoiding extraction and purification steps (**Figure** **24**). For example, the ammonia (NH_3_) produced can be used directly in fertilizer synthesis, while hydrogen and hydrogen peroxide can be used in fuel cells to generate energy. Furthermore, hydrogen peroxide produced can be used directly for disinfection and wastewater treatment, especially in remote areas. This strategy will help to synthesize commercially essential products, opening up new avenues for the development of COFs for practical applications in the future.
vii)
**Utilization of sunlight in photocatalytic experiments**: Basically, photocatalytic reactions require light sources of different wavelengths depending on the bandgap of the photo‐absorber or photosensitizer. Although photocatalytic experiments in the laboratory are performed with artificial light of various wavelengths, however, for their practical applications, sunlight will be the ideal sustainable source since solar light is composed of light (<400 nm, 4.6%), visible light (400–700 nm, 47.7%) and remaining near‐infrared light. To effectively use sunlight for photocatalysis, it is essential to design materials that can harvest maximum light. However, different photocatalytic reactions require specific potentials or bandgaps; for instance, photocatalytic overall water splitting requires 1.23 eV, so ideally, the photocatalyst can use light until ≈1000 nm. The bandgap of COFs can be tuned by easy integration of photosensitizing units and controlling the extended π‐conjugation of COF photocatalysts.

A perhaps even more critical point in the utilization of sunlight for photocatalysis is often forgotten. The average solar radiation on earth at sea level is ≈1000 W m^−2^. With an reaction enthalpy for water splitting of ≈285 kJ/mol, which corresponds to an energy content of ≈40 kWh in 1 kg of gaseoushydrogen, we would therefore need at least an area of 40 m^2^ to provide the energy required to produce 1 kg of hydrogen in 1 h. And this is only for the case for a solar‐to‐chemical‐conversion efficiency of 100%, which is far from realistic. For the quantities of hydrogen that mankind currently needs and will need in the future for a sustainable transformation of industry, this space requirement is an enormous challenge. The figures for CO2RR or ammonia production will not differ too much from these quantities. In addition to catalyst design, it will therefore be necessary to take pragmatic approaches, such as developing new photocatalytic reactors probably using mirrors to concentrate sunlight, a concept which is already considered in photovoltaics, e.g., in concentrator modules. It might be therefore advisable to test new photocatalysts in the laboratory not only with light intensity from one sun, but also at much higher intensities of several suns, to finally judge if a photocatalyst can make it to real applications.

In summary, the use of COFs in heterogeneous photocatalysis is a highly relevant and dynamic field of research, which still raises many attractive but also challenging questions. If these can be solved, COFs could make a significant contribution to a future sustainable energy supply for our society.

## Conflict of Interest

The authors declare no conflict of interest.
